# Elaboration of the Effective Multi-Target Therapeutic Platform for the Treatment of Alzheimer’s Disease Based on Novel Monoterpene-Derived Hydroxamic Acids

**DOI:** 10.3390/ijms24119743

**Published:** 2023-06-04

**Authors:** Yulia Aleksandrova, Aldar Munkuev, Evgenii Mozhaitsev, Evgenii Suslov, Dmitry Tsypyshev, Kirill Chaprov, Roman Begunov, Konstantin Volcho, Nariman Salakhutdinov, Margarita Neganova

**Affiliations:** 1 Institute of Physiologically Active Compounds at Federal Research Center of Problems of Chemical Physics and Medicinal Chemistry, Russian Academy of Sciences, Severnij Pr. 1, Chernogolovka 142432, Russia; 2 Department of Medicinal Chemistry, N. N. Vorozhtsov Novosibirsk Institute of Organic Chemistry, Siberian Branch, Russian Academy of Sciences, Lavrentiev Ave., 9, Novosibirsk 630090, Russia; 3 Biology and Ecology Faculty of P. G. Demidov Yaroslavl State University, Matrosova Ave., 9, Yaroslavl 150003, Russia

**Keywords:** hydroxamic acids, Alzheimer’s disease, transgenic mice model, molecular target, docking study, histone deacetylase 6, radical scavenging, amyloid β-protein, learning and memory

## Abstract

Novel monoterpene-based hydroxamic acids of two structural types were synthesized for the first time. The first type consisted of compounds with a hydroxamate group directly bound to acyclic, monocyclic and bicyclic monoterpene scaffolds. The second type included hydroxamic acids connected with the monoterpene moiety through aliphatic (hexa/heptamethylene) or aromatic linkers. An in vitro analysis of biological activity demonstrated that some of these molecules had powerful HDAC6 inhibitory activity, with the presence of a linker area in the structure of compounds playing a key role. In particular, it was found that hydroxamic acids containing a hexa- and heptamethylene linker and (-)-perill fragment in the Cap group exhibit excellent inhibitory activity against HDAC6 with IC_50_ in the submicromolar range from 0.56 ± 0.01 µM to 0.74 ± 0.02 µM. The results of the study of antiradical activity demonstrated the presence of moderate ability for some hydroxamic acids to scavenge 2,2-diphenyl-1-picrylhydrazyl (DPPH) and 2ROO^•^ radicals. The correlation coefficient between the DPPH radical scavenging activity and oxygen radical absorbance capacity (ORAC) value was R^2^ = 0.8400. In addition, compounds with an aromatic linker based on para-substituted cinnamic acids, having a monocyclic para-menthene skeleton as a Cap group, **35a**, **38a**, **35b** and **38b**, demonstrated a significant ability to suppress the aggregation of the pathological β-amyloid peptide 1-42. The **35a** lead compound with a promising profile of biological activity, discovered in the in vitro experiments, demonstrated neuroprotective effects on in vivo models of Alzheimer’s disease using 5xFAD transgenic mice. Together, the results obtained demonstrate a potential strategy for the use of monoterpene-derived hydroxamic acids for treatment of various aspects of Alzheimer’s disease.

## 1. Introduction

Alzheimer’s disease is the most common neurodegenerative disease that causes late dementia in elderly people [1,2,3]. Being a complex, multifactorial and inevitably progressive pathology [2,4,5], Alzheimer’s disease is associated with a high level of morbidity and mortality [6,7,8]. It causes significant financial costs to the government and families of patients with this disease for treatment and patient care [9,10,11].

Due to the fact that over the past two decades, only one drug, Aducanumab, has passed the FDA approval procedure for use as an anti-Alzheimer’s pharmacological agent [12,13,14,15,16,17,18], and the statistics of mortality from Alzheimer’s disease has increased by almost 50% since 2000 [19], the attention of researchers around the world is focused on serious efforts in the discovery and development of new therapeutic agents for the treatment of this disease.

**Figure 1:**
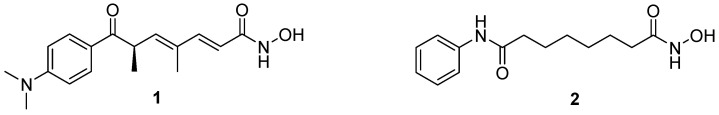
Chemical structures of representatives of the class of hydroxamic acids with neuroprotective propertie—trichostatin A (**1**) and vorinostat (**2**).

In recent years, the scientific community has been steadily gaining momentum in the strategy of repurposing existing medicines in order to expand the therapeutic spectrum of action by discovering new indications for use [20,21,22,23,24,25]. One of these drugs is the well-known antitumor and antifungal agent trichostatin A (**1**), a representative of the hydroxamic acid class (Figure 1). It demonstrated neuroprotective effects in a number of experimental studies. It was found that trichostatin A has the ability to reduce neuroblastoma SH-SY5Y cell damage induced by a pathological β-amyloid peptide due to its antioxidant properties and normalization of Nrf2 signaling [26]. In experiments on transgenic animals, trichostatin A increases the levels of the gelsolin protein responsible for the clearance of Aß in blood samples, as well as in the brain of mice [27,28]. Su et al. demonstrated that the chronic administration of trichostatin A has a positive effect on the short-term episodic and long-term spatial memory of APP/PS1 mice modelling Alzheimer’s disease [29]. The therapeutic potential in various diseases affecting the brain has been convincingly proven for Vorinostat (**2**), the FDA-approved antitumor agent against T-cell lymphoma (Figure 1) [30]. In particular, the positive properties of the use of this hydroxamic acid were noted in the treatment of Alzheimer’s disease [30,31,32], Parkinson’s disease [33], Huntington’s chorea [34,35] and other degenerative conditions.

Such examples led to the activity of a large number of research groups in the direction of creating new promising compounds based on hydroxamic acid with an expanded profile of neuroprotective activity. In particular, in our study, we report the synthesis and biological activity of novel monoterpene-derived hydroxamic acids, developed as potential neuroprotective agents with a multi-target type of action by including various pharmacophore fragments with an already known bioactivity in the molecule.

The use in our work of the approach of adding monoterpenoid derivatives to the structure of hydroxamic acids can help to make an unconditional contribution to the strengthening of neuroprotective properties of compounds. For example, limonene has been demonstrated to inhibit neurotoxicity caused by the pathological form of β-amyloid 1-42 in a Drosophila model [36], primary cortical neurons [37] and rats [38]. In addition, a significant number of studies indicate the presence of antioxidant properties of this monoterpene [39], due to a decrease in the content of malondialdehyde and an increase in the levels of enzymes of its own antioxidant system-superoxide dismutase, catalase and glutathione [40]. All this testifies to the expediency and prospects of using the above-described approach in our study.

Thus, herein we report the development, synthesis and biological evaluation of the neuroprotective properties of novel hydroxamic acids combining monoterpene fragments and a hydroxamate function, both directly and through linkers of various natures.

## 2. Results

### 2.1. Chemistry

**Scheme 1:**
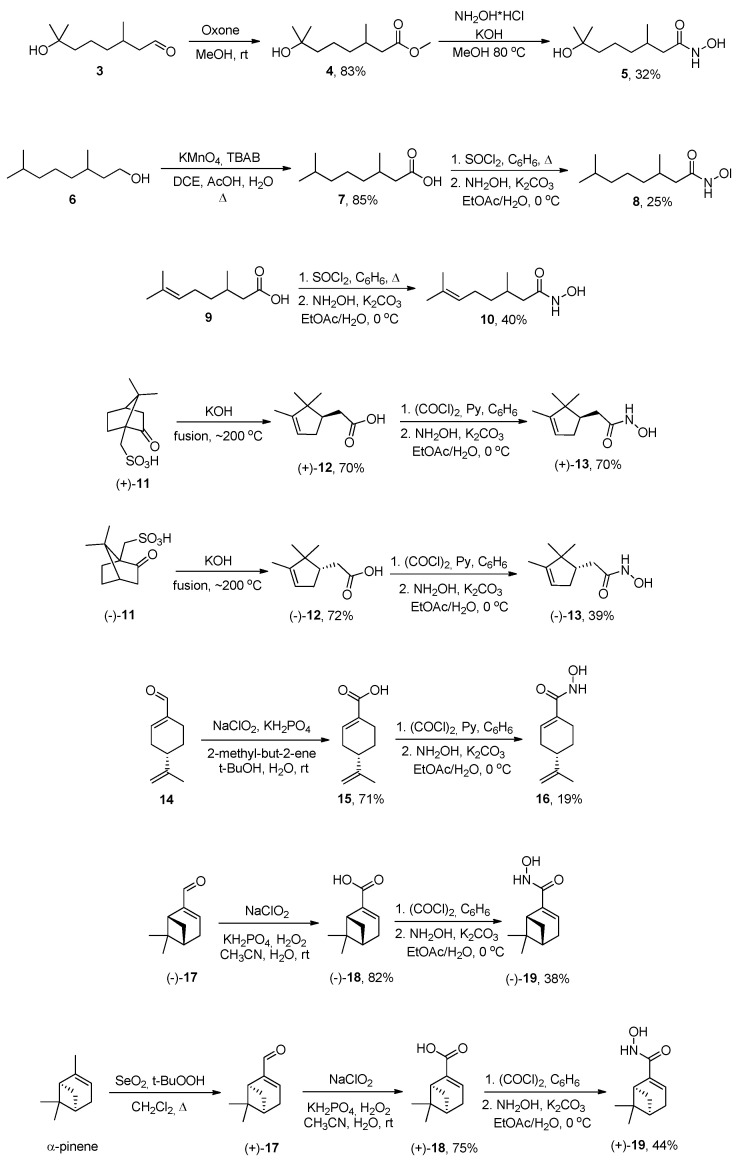
Synthesis of target compounds **5**, **8**, **10**, **13**, **16** and **19**.

First, we decided to obtain compounds with the hydroxamate group attached directly to the monoterpene moiety (Scheme 1). To achieve that, 7-hydroxycitronellal **3** was transformed into ester **4** using oxone in methanol, which then reacted with hydroxylamine by heating the sealed vessel containing a methanol solution of methyl ester **4**, hydroxylamine hydrochloride and KOH to provide hydroxamic acid. **5**. 3,7-Dimethyloctane-1-ol **6** was oxidized with the corresponding carboxylic acid **7** by potassium permanganate in a CH_2_Cl_2_/AcOH/water system in the presence of tetrabutylammonium bromide as a phase transfer catalyst. The synthesis of N-hydroxy-carboxamide **8** was carried out by the successive activation of the carboxylic group with thionyl chloride and treatment with hydroxylamine in a H_2_O/EtOAc mixture containing potassium carbonate at 0 °C. Target hydroxamic acids **10**, **13**, **16** and **19** were prepared from the corresponding carboxylic acids in a similar manner, using (COCl)_2_ as the chlorinating agent instead of SOCl_2_. The synthesis of (+)-campholenic and (-)-campholenic acids **12** was performed by the fusion of camphorsulfonic acids **11** with KOH [41]. Conjugated acids **15** and **18** were prepared by the oxidation of aldehydes **14** and **17** with NaClO_2_ in the buffer system (Pinnick oxidation) according to the method [42].

**Scheme 2:**
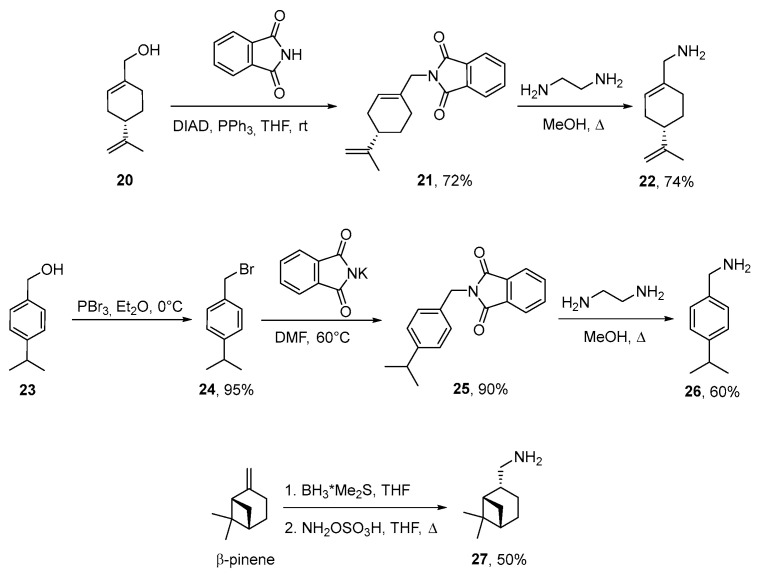
Synthesis of monoterpene amines **22**, **26** and **27**.

To obtain compound **21**, the Mitsunobu reaction was carried out between perillyl alcohol **20** and phthalimide in the presence of diisopropyl azodicarboxylate (DIAD) and triphenylphosphine (PPh_3_) (Scheme 2). The synthesis of phthalimide derivative **25** was commenced from cumyl alcohol **23** using a two-step procedure involving the formation of a C-Br bond (compound **24**) followed by the reaction with potassium phthalimide in DMF. The subsequent treatment of phthalimides **21** and **25** with ethylenediamine in methanol gave monoterpene amines **22** and **26** (Scheme 2). Myrtanyl amine **27** was synthesized by a reaction of β-pinene with borane dimethylsulfide followed by treatment with hydroxylamine-O-sulfonic acid (Scheme 2).

**Scheme 3:**
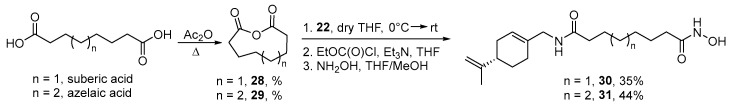
Synthesis of hydroxamic acids **30** and **31**.

In order to synthesize hydroxamic acids with aliphatic linkers, anhydrides **28** and **29** were prepared by refluxing a solution of suberic or azelaic acid in acetic anhydride (Scheme 3). The subsequent treatment of compounds **28** and **29** with amine **22** followed by ethyl chloroformate and NH_2_OH led to the formation of hydroxamic acids **30** and **31**.

**Scheme 4:**
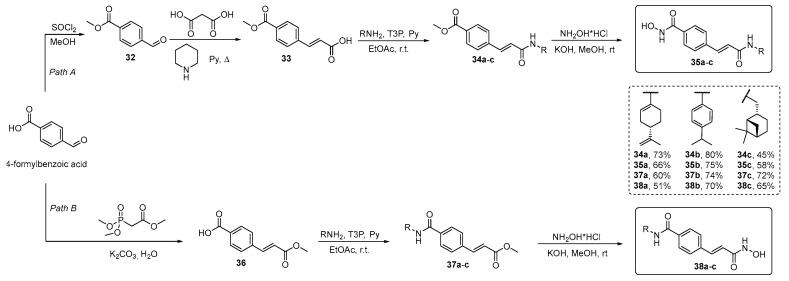
Synthesis of substances **35a**–**c**> and **38a**–**c**>.

For the synthesis of target compounds bearing aromatic linkers, 4-formylbenzoic acid was used as a starting compound. To obtain target hydroxamic acids **35a**–**c**> (path A), 4-formylbenzoic acid was converted into the corresponding ester **32**. Cinnamic acid **33** was synthesized by a reaction of compound **32** with malonic acid in the presence of pyridine and piperidine. Amide bond formation was performed utilizing n-propanephosphonic acid anhydride (T3P) as a coupling reagent to obtain derivatives **34a**–**c**>, which were then treated with NH_2_OH in MeOH to form target hydroxamic acids **35a**–**c**> (Scheme 4).

To execute path B, the Horner–Wadsworth–Emmons reaction of 4-formylbenzoic acid with trimethyl phosphonoacetate was carried out to synthesize compound **36**. The next synthetic sequence included amide coupling to form amides **37a**–**c**> followed by NH_2_OH treatment, which resulted in target compounds **38a**–**c**> (Scheme 4).

All synthesized compounds were evaluated for the presence of neuroprotective activity on the effects on key links in the pathogenesis of Alzheimer’s disease.

### 2.2. Inhibition of HDAC6 Activity: In Vitro and Molecular Docking

First, we analyzed the HDAC6-inhibitory ability of synthesized hydroxamic acids using a commercially available kit. In the last decade, there has been a genuine interest in the study of the HDAC6-inhibitory activity of potential therapeutic agents for the treatment of Alzheimer’s disease. This is due to an increase in the expression of the 6th isoform of histone deacetylase found in the brain tissues of patients with this disorder, which correlates with neuronal death and cognitive dysfunction. In this regard, an urgent strategy in the creation of anti-Alzheimer’s drugs is the search for compounds capable of controlling the HDAC6 activity.

The change in HDAC6 activity was determined by a method based on measuring the kinetics of substrate deacetylation in the enzyme presence. A well-known specific HDAC inhibitor, trichostatin A, was used as a positive control to confirm the operation of the model system, for which the IC_50_ HDAC6 inhibitory effect was in the nanomolar range (12.29 ± 1.72 nM). This is consistent with the data already known for trichostatin A that is presented in [43,44].

Table 1 shows the values of the half maximum inhibitory concentration (IC_50_) of HDAC6-inhibitory activity. The lowest IC_50_ values were found for compounds **30** and **31** with a hexa-and heptamethylene linker motif and (-)-perill fragment in the Cap group, which were 0.56 ± 0.01 µM and 0.74 ± 0.02 µM, respectively. Compounds with an aromatic linker based on para-substituted cinnamic acids also had a high HDAC6-inhibiting ability. Thus, the IC_50_ values for hydroxamic acids containing Cap groups belonging to the bicyclic pinane backbone—**35c** and **38c**, and the monocyclic paramentane backbone—**35a**, **35b**, **38b** and **38a**, varied from 3.85 ± 0.24 µM to 8.23 ± 0.40 µM.

In turn, hydroxamic acids **5**, (-)-**19**, **10**, **16**, (+)-**19**, **8**, (+)-**13** and (-)-**13** demonstrated no significant ability to inhibit the enzymatic activity of HDAC6, which is obviously due to the absence of a linker in the structure of the molecules.

Due to the fact that hydroxamic acids **31**, **30**, **35a**, **38b**, **38a**, **35b**, **35c** and **38c** demonstrated the most promising profile of HDAC6 inhibition, which is interesting for expanding the understanding of the interaction of compounds with this protein, we performed the docking procedure (Table 2). Taking into account the spectrum of action of the catalytic domain 2 [45] and the literature data on in silico procedures [46], the structure of the HDAC6 complex and trichostatin A bound to a protein in the second catalytic domain [47] was used for docking procedures.

For all hydroxamic acids included in the docking procedure, the docking position is reproduced similarly to the reference trichostatin A, where the hydroxamate group is oriented by the zinc atom.

The position of the cis-pinane hydrophobic cap group in the active center for the corresponding compounds (**35c**, **38c**) differs significantly from the position of the cap group of (-)-perillic and cumylic types (**30**, **31**, **35a**, **35b**, **38a** and **38b**). The (-)-perillic and cumylic cap groups in the docking positions of the corresponding compounds are located within the same protein fold (between the fragment from ASN494 to PRO501 and the fragment from ASP567 to CYT572), while the bulky cis-pinane fragment occupies different positions (in **35c**—in the pocket between the loop from THR678 to GLY683 and the loop from ASP567 to ILE569; in **38c**—on a loop from ASP497 to PRO501). This results in a difference in the geometries of the reproduced poses and a difference in the interactions between the protein and the ligand.

A significant number of hydrogen bonds were reproduced for compounds with flexible linkers (**30** and **31**) and for compounds with the “hydroxamate-phenyl-ethylene” linker (**35a**–**35c**). Compounds **30**, **31**, **35a** and **35b** interact with three of the five amino acids from the list HIE500, SER568, HIS610, GLY619 and TYR782; compound **35c** forms hydrogen bonds with two of the five amino acids-SER568 and GLY619. Compounds with the linker “hydroxamate-phenyl-ethylene” (**35a**–**35c**) form *π*-*π* stacking interactions with two of the three amino acids from the list HIE500, PHE620, HIE651 and PHE680. Compounds with the “hydroxamate-ethylene-phenyl” linker (**38a**–**38c**) participate in fewer interactions by hydrogen bonds and *π*-*π* stacking: they are characterized by single hydrogen bonds with SER568, GLY619 and TRY782, respectively, and *π*-*π* stacking interactions are observed only for **38b** and **38a**, and with the amino acid PHE680 for both compounds.

**Figure 2:**
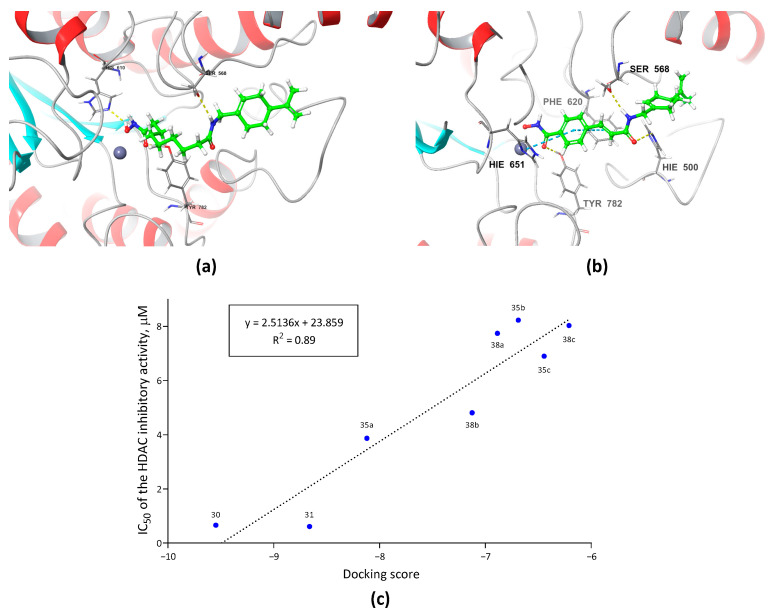
Docking poses of **31** (**a**) and **35a** (**b**) on the crystal structure of the HDAC6 isoenzyme (PDB: 5EDU). The zinc atom is shown as a grey sphere. Nitrogen and oxygen atoms are colored in blue and red, respectively. Hydrogen bonds are shown in yellow dotted lines, *π*-*π* stacking interactions are shown in light blue dotted lines. (**c**) The correlation between the calculated docking scores and the experimentally obtained values of IC_50_ HDAC6 inhibitory effect.

Figure 2a displays the possible docking positions of hydroxamic acid **31** with HDAC6, where the hydroxamate group and the linker region establish effective interactions with HDAC6. In turn, for the cap group representing the (-)-perillyl fragment, an interaction with loops around the ligand-binding pocket is observed.

Figure 2 shows the possible docking positions of hydroxamic acids **31** and **35a** with HDAC6, where the hydroxamate group and the linker region establish effective interactions with HDAC6. In turn, for the cap group representing the (-)-perillyl fragment, an interaction with loops around the ligand-binding pocket is observed.

A correlation graph linking the values obtained from the docking (Docking Score) and the experimentally obtained values of IC_50_ for compounds **31**, **30**, **35a**, **38b**, **38a**, **35b**, **35c** and **38c** is shown in Figure 2. Taken together, our results demonstrate a good correlation with a value of R^2^ = 0.89.

### 2.3. Determination of Antioxidant Activity through Radical Measurement

At the next stage, we investigated the antiradical ability of synthesized hydroxamic acids using two tests—the DPPH and ORAC experiments.

DPPH is a stable chromogen radical, which is widely used to assess the antioxidant activity of biological objects, in particular when searching for potential therapeutic agents used to maintain the redox balance in the body and treat pathologies associated with the action of free radicals [49,50]. In turn, the oxygen radical absorbance capacity (ORAC) analysis is based on the removal by potential antioxidants of peroxyl radicals generated by AAPH (2,2’-axobis-2-methyl-propanimidamide, dihydrochloride), preventing the degradation of the fluorescein probe.

**Figure 3:**
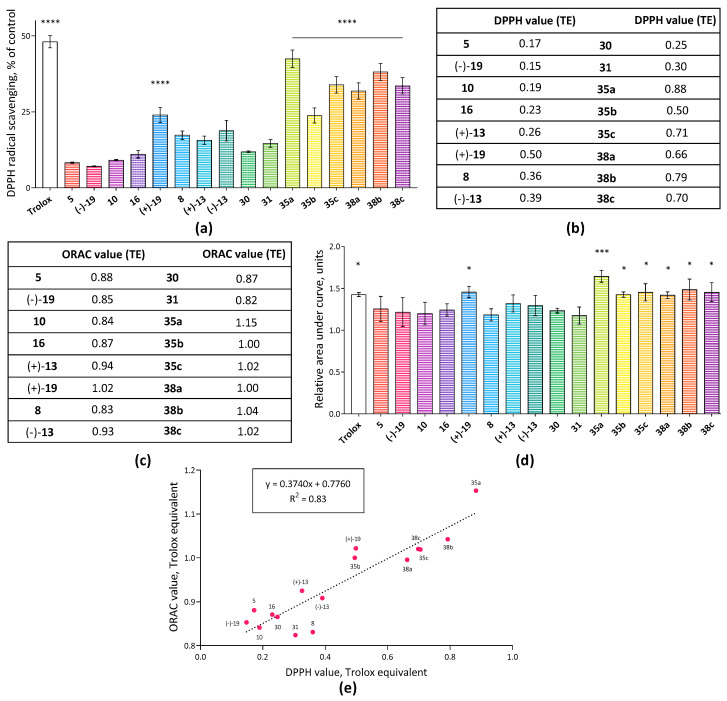
Antioxidant potential of novel monoterpene-derived hydroxamic acids: (**a**) DPPH radical scavenging activity. (**b**) DPPH values expressed as Trolox equivalent (TE). (**c**) ORAC values expressed as TE. (**d**) Oxygen radical absorbance capacity. (**e**) The correlation between DPPH and ORAC values. The concentration of the studied compounds and Trolox was 100 µM, DPPH—100 µM and AAPH—12 mM. To assess statistical significance, one-way ANOVA and Dunnett multiple comparison tests were used, where **** *p* < 0.0001, *** *p* < 0.001 and * *p* < 0.05 vs. Control.

Figure 3 shows the results of the study of the antiradical activity of hydroxamic acids. Moderate DPPH radical scavenging activity was found for hydroxamic acids (+)-**19**, **35a**, **35b**, **35c**, **38a**, **38b** and **38c**. For these compounds, the percentage of antiradical activity varied from 23.78 ± 2.49% to 42.44 ± 2.88%.

The results of the ORAC analysis also confirmed the antioxidant potential of hydroxamic acids. Similarly to the results of the study of DPPH radical scavenging activity for compounds (+)-**19**, **35a**, **35b**, **35c**, **38a**, **38b** and **38c**, the greatest ability to absorb peroxyl radicals formed as a result of the decomposition of 2,2’-azobis (2-amidinopropane) dihydrochloride was found. At the same time, it was for hydroxamic acid **35a** that the most pronounced antiradical properties were revealed in both experiments, as evidenced by the value of ORAC TE (trolox equivalent), 1.15.

Figure 3 shows a correlation graph linking the values obtained in these tests. Our results demonstrated a positive correlation when comparing DPPH and ORAC values with R^2^ = 0.83.

### 2.4. Inhibition of Aβ_1-42_ Aggregates: In Vitro and Molecular Docking

**Figure 4:**
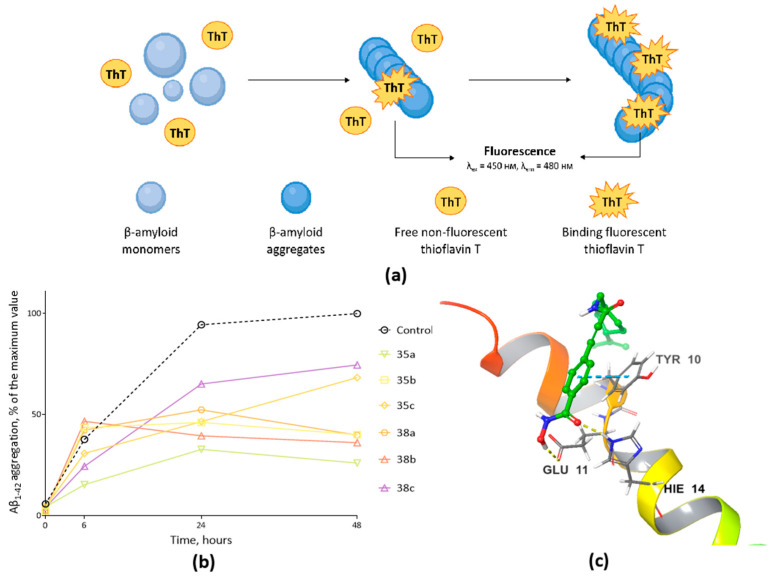
(**a**) The principle of interaction of thioflavin T with β-amyloid. Thioflavin T (ThT) binds to the aggregated structure of β-amyloid, penetrating into the β-layers of the protein, as a result of which its fluorescence is detected. (**b**) Thioflavin T (ThT) fluorescent analysis of the effect of novel monoterpene-derived hydroxamic acids on the aggregation of the pathological form of β-amyloid peptide 1-42. The concentration of the studied compounds was 100 µM, Thioflavin T-10 µM. (**c**) A general idea of the molecular docking of hydroxamic acid **35a** with β-amyloid (PDB ID: 1IYT [56]). The **35a** molecule is represented in green. Nitrogen and oxygen are colored in blue and red, respectively. Hydrogen bonds are shown in yellow dotted lines; *π*-*π* stacking interactions are shown in light blue dotted lines. β-amyloid’s α-helix is colored according to residue number, from red to purple.

The aggregation process of β-amyloid peptide 1-42 (Aβ_1-42_), caused by hydrophobic interactions of peptide monomers and, as a consequence, the formation of the most toxic polymorphic structure-oligomeric fragments of Aβ, was detected using the fluorescent dye Thioflavin T (Figure 4a). This method is the most widely used for selective staining and the identification of amyloid aggregates [51], which is confirmed by a large number of works [52,53].

Similar to previous studies [54,55], a typical S-shaped curve was observed in control samples (Figure 4b), indicating the rapid formation of protein aggregates during the first 24 h, followed by an equilibrium phase without a significant increase in the fluorescence signal when analyzing samples after 48 h. Figure 4b also shows the kinetic curves of the formation of toxic forms of oligomers under the action of compounds that have demonstrated the ability to suppress the aggregation of the pathological β-amyloid peptide 1-42. It is easy to observe that only four substances were found to have excellent inhibitory activity. Thus, in samples with hydroxamic acids **35a**, **38a**, **35b** and **38b** at a concentration of 100 µM, the significantly reduced fluorescence of thioflavin T was observed throughout the experiment, indicating the ability to prevent the aggregation of Aβ_1-42_ by 73.93%, 60.09%, 59.88% and 63.86%, respectively.

There are known examples of docking compounds into β-amyloid structures, both in the monomer and in the fibrils formed by it [57,58]. To assess the binding of hydroxamic acids to the peptide, which could interfere with the aggregation of monomers, docking was performed in the monomer binding pockets. To determine the binding pockets, an initial “blind” docking was carried out over the entire surface of the peptide. The analysis of the obtained results indicates the two most energetically advantageous binding pockets: near the C-terminus of the peptide and in the bend near the N-terminus of the peptide, which is consistent with the literature data [57,58]. After clarifying the coordinates of the binding pockets, refining calculations were performed, and an estimate of the Gibbs binding energy for hydroxamic acids in both positions was obtained. The Gibbs binding energy estimate was used to calculate the relative probabilities of realizing a particular binding position for a given hydroxamic acid.

As an example, Figure 4b shows a visual identification of the binding position of hydroxamic acid with an aromatic linker and a cap group containing the (-)-perillyl fragment **35a**, which exhibits an excellent ability to inhibit the aggregation of β-amyloid. Molecular docking confirmed the possibility of interaction of this compound with the peptide, and statistical processing of the results (assuming a temperature equal to 298 K) demonstrated that binding to both the C-terminus and the bend near the N-terminus is possible, with binding to the C-terminus being 15 times more likely relative to the latter. The formation of the “**35a**-protein” complex upon binding to the pocket near the C-terminus is due to the interaction of hydroxamic acid through hydrogen bonds with GLU11 and HIE14, as well as the π-π-stacking interaction with TYR10.

It is worth noting that compound **35a** has the least selectivity for the binding pocket in comparison with its analogs when forming bonds with the pocket at the N-terminus. For other compounds, binding to only one fragment of the β-amyloid monomer is predicted: **38a** binds to the C-terminus 77 times more likely than to the bend at the N-terminus; **38b** is 230 times more likely to form a bond with the N-pocket than with the C-terminus; for **35b**, the probability ratio is 480:1 in favor of the C-terminus; for **35c**—2700:1 in favor of the N-bend, and for **38c**—290,000:1 in favor of the C-terminus.

### 2.5. Influence on Cell Viability

To assess possible cytotoxic effects of the novel monoterpene-derived hydroxamic acid, their effect on the survival of the neuronal-like neuroblastoma cell line SH-SY5Y was determined. As shown in Table 3, none of the studied compounds had a pronounced cytotoxic activity against the used cell culture.

Thus, the results obtained indicate the possibility of moving to the next in vivo level of investigation of the neuroprotective potential of hydroxamic acids due to the absence of possible restrictions in the form of toxic side effects on the body.

To evaluate the pharmacokinetic properties of compounds, we obtained ADME-Tox descriptors using the QikProp subroutine of the Schrodinger Suites software package (Table 4) [59].

All of the profiled compounds have a low number of stars (*), ranging from 0 to 1, which indicates that they share similar properties with other drug-like compounds. Compounds adhere to Lipinski’s rule of five (RO5) with no violations, and only compounds **30** and **31** have one violation of Jorgensen’s rule of three (RO3), while all other compounds exhibit no violations. The logPo/w values for all compounds are within the acceptable range, signaling favorable partition coefficients. All compounds have less than 8 probable metabolic reactions that they can participate in, the logS values are within the acceptable range, the PCaco values for all compounds are above 25 nm/s, with some even above 200 nm/s, and the predicted human oral absorption for all compounds lies in the moderate range from 70% to 84%. These findings suggest that the compounds are drug-like and likely to be orally available.

Based on the ADME/Tox descriptors, the provided compounds appear to be sufficiently drug-like, and show promise for further investigation as potential drug candidates.

### 2.6. Selection of the Leader Compound 

**Figure 5:**
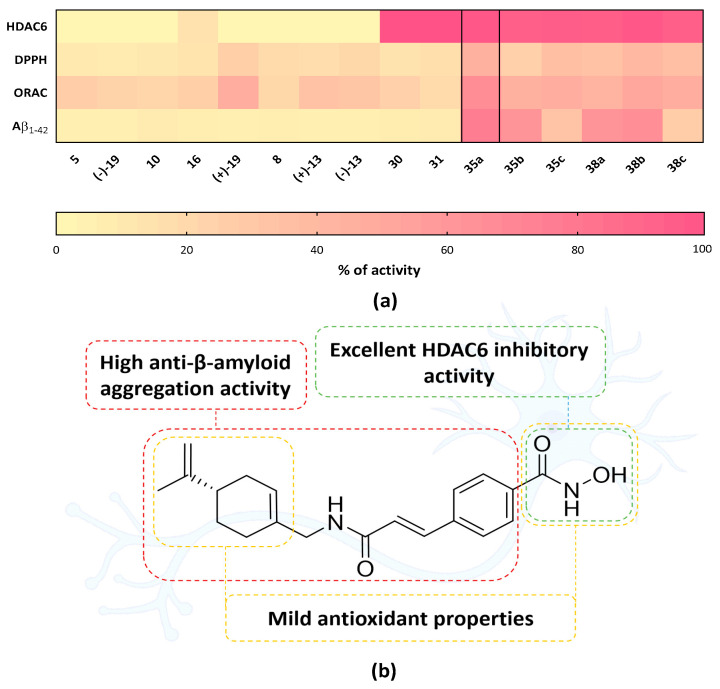
(**a**) Heat map of the studied parameters of the biological activity of novel monoterpene-derived hydroxamic acid. The exact averages and errors of the mean are presented in the corresponding subsections of each individual experiment. The smooth transition of the gradient from yellow to pink indicates an increase in activity in a particular test. HDAC6—inhibition of histone deacetylase 6; DPPH—2,2-diphenyl-1-picrylhydrazyl; ORAC—oxygen radical absorption capacity; Aß1-42—inhibition of β-amyloid aggregation. (**b**) The spectrum of the detected biological activity of the leader compound-hydroxamic acid with an aromatic linker and a Cap group containing (-)-perill fragment **35a**.

At the end of the in vitro series of studies of the biological activity of novel monoterpene-derived hydroxamic acids, we analyzed the structure–activity relationship to select the most promising leader compound for further animal testing. All the results expressed in the percentage of activity are summarized on the heat map (Figure 5). The image clearly shows that the leader compound turned out to be hydroxamic acid with an aromatic linker and a Cap group containing (-)-perillyl fragment **35a** (the contour is marked with a black border). The choice of this compound is due to the spectrum of potential mechanisms of therapeutic action manifested by it, including pronounced HDAC6-inhibitory properties, antiradical activity in DPPH and ORAC tests and the ability to prevent aggregation of the pathological form of β-amyloid peptide without significantly affecting the survival of neuronal-like SH-SY5Y cell culture.

### 2.7. Protective Effect of **35a** against Cognitive Deficits of 13-Month Old 5xFAD Mice

In order to prove the therapeutic effect of the **35a** leader compound on an in vivo 5xFAD transgenic mice neurodegeneration model, cognitive functions were evaluated in the Morris Water Maze test.

**Figure 6:**
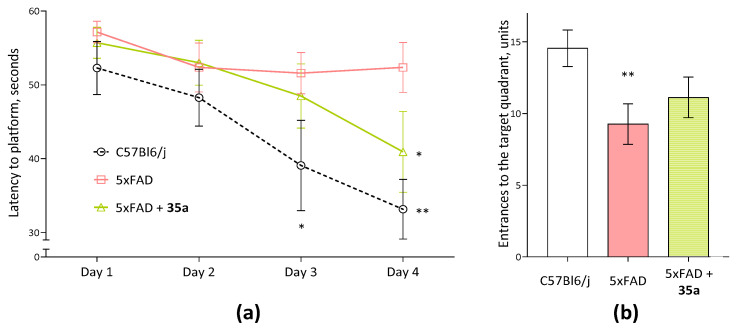
(**a**) Latency to platform of the four-day Morris water maze. * *p* < 0.05 and ** *p* < 0.01 vs. Day 1. (**b**) Amount of entrances in the target quadrant in the Morris water maze. ** *p* < 0.01 vs. C57Bl6/j. Data represent the mean ± SEM (*n* = 8 per group). One-way ANOVA and Dunnett multiple comparison tests were used to assess statistical significance.

As shown in Figure 6a, in a four-day Morris water maze trial involving mice searching for a platform hidden under water, animals of the 5xFAD line demonstrated a similar period of time required to reach the platform. This indicated an obvious decrease in cognitive functions in transgenic animals. In turn, the administration of hydroxamic acid **35a** at a dose of 15 mg/kg reduced this indicator from 55.70 ± 2.10 s (on the first day of training) to 40.93 ± 5.47 s (on the fourth day of training) (*p* = 0.039). A similar pattern was observed in the group of clinically healthy wild-type mice; however, in this case, a significant decrease in the latency period of reaching the platform was already observed on the third day of the experiment. This parameter decreased from 52.27 ± 3.60 s (on the first day of training) to 39.09 ± 6.13 s and 33.16 ± 4.05 s (on the third and fourth days, respectively) (*p* = 0.033 and *p* = 0.004, respectively).

On the fifth day of the experiment, the neuroprotective potential of **35a** was confirmed in the Probe trial session, where the same number of entrances to the target quadrant of the maze was found with the control group (*p* = 0.203), while this indicator was significantly lower for animals from the 5xFAD group (*p* = 0.006 vs. C57Bl6/j and *p* = 0.014 vs. 5xFAD + **35a**).

### 2.8. Oxidative Damage Decreasing and Mitochondrial Function Improving in the 5xFAD Mice Brain by **35a**

To determine the level of lipid peroxidation in brain samples obtained after in vivo testing in mice of experimental groups, the content of malondialdehyde (MDA) was measured. A one-way ANOVA demonstrated that there were significant differences in this indicator between control wild-type and transgenic 5xFAD mice. Thus, the MDA level in genetically modified animals was increased by more than 30% (*p* < 0.0001). This increase in the oxidative stress marker in the brain was reversed with a 21-day administration of hydroxamic acid **35a** (*p* = 0.001), almost to the level of clinically healthy mice of the C57Bl6/j line (*p* = 0.011).

**Figure 7:**
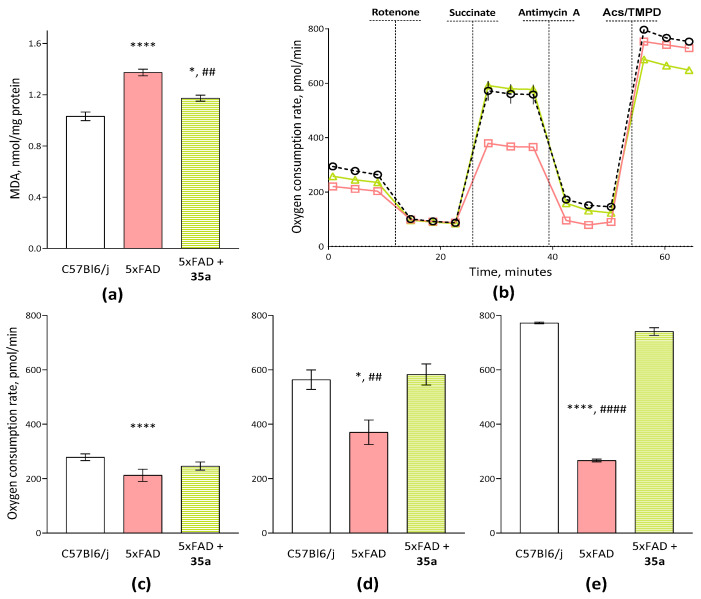
(**a**) Malondialdehyde levels in mouse brain samples. (**b**–**e**) Evaluation of the bioenergetic profile of the synaptosomal-mitochondrial (p2) fraction obtained from mouse brain samples by measuring the rate of oxygen consumption as a result of sequential addition of modulators: glutamate (10 mm) and malate (10 mM)-substrates of the I complex, rotenone (2 µM)-inhibitor of the I complex and succinate (10 mM)-the substrate of the II complex; ascorbate (10 mM) and TMPD (100 µM) are the substrates of the IV complex. (**b**) Kinetic curves of changes in the rate of oxygen consumption by organelles. (**c**–**e**) Data presented as histograms, in which each column represents the average value of the OCR by the organelles of the experimental groups (mean ± SEM, n = 8). To assess statistical significance, one-way ANOVA and Dunnett’s multiple comparison tests were used, where ****, #### *p* < 0.0001 vs. C57Bl6/j and 5xFAD, respectively; ## *p* < 0.01 vs. 5xFAD, * *p* < 0.05 vs. C57Bl6/j.

To identify changes in the respiratory chain of the mitochondria of the 5xFAD transgenic mice brain, we measured respiration mediated by I, II and IV complexes of the electron transport chain of organelles. As shown in Figure 7, mutations observed in genetically modified animals had an effect on respiration mediated by all three complexes studied. This was evidenced by a decrease in the rate of oxygen uptake by the synaptosomal-mitochondrial (p2) fraction with the sequential introduction of substrates of NADH-dehydrogenase (Figure 7c), succinate dehydrogenase (Figure 7d) and cytochrome-C-oxidase (Figure 7e) complexes by 23.87% (*p* = 0.015), 34.26% (*p* = 0.004) and 65.43% (*p* < 0.0001), respectively. However, the activity of complexes II and IV in the organelles of transgenic mice treated with hydroxamic acid **35a** for three weeks had significantly higher rates by 57.30% (*p* = 0.0025) and 177.57% (*p* < 0.0001), respectively.

### 2.9. Reduce Number of β-Amyloid Deposits in the 5xFAD Mice Brain by Protective Effect of **35a**

**Figure 8:**
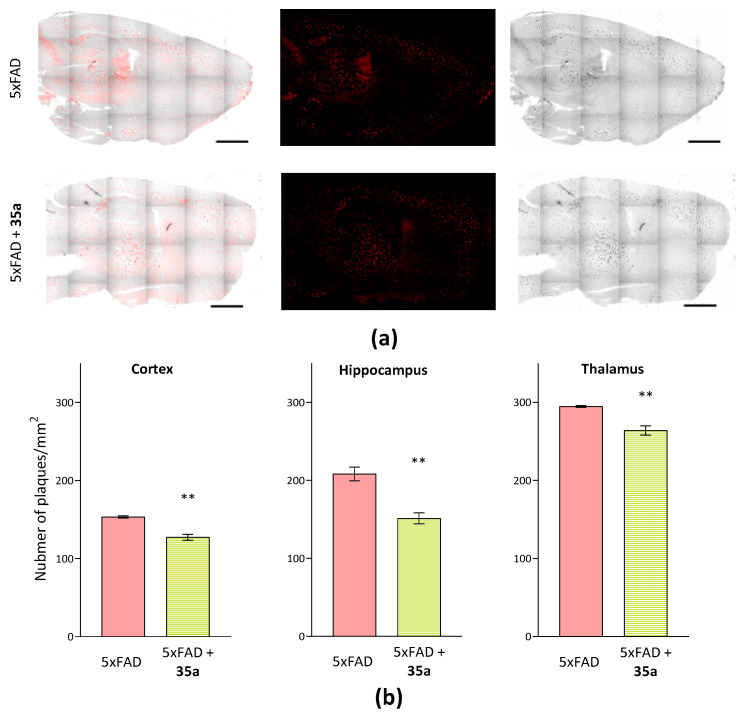
Histological analysis of β-amyloid deposits in brain sections of transgenic 5xFAD mice after administration of hydroxamic acid **35a** for 21 days. (**a**) Representative mouse brain images; staining with Congo Red. Bar, 1000 μm. (**b**) A morphometric analysis in selected zones. ** *p* < 0.01 vs. 5xFAD.

To estimate the amount of β-amyloid inclusions in the selected zones, brain sections were stained with Congo Red. Large amyloid deposits, along with small and medium-sized ones, were detected in the cortex, thalamus and hippocampus in transgenic 5xFAD mice (Figure 8a). The results of morphometric analysis of β-amyloid inclusions of 5xFAD и 5xFAD + **35a** groups are shown in Figure 8b. The number of aggregates in the brain decreased significantly after a 21-day administration of hydroxamic acid **35a** in cortex, midbrain and hippocampus zone.

## 3. Discussion

According to the World Health Organization, Alzheimer’s disease is considered a “global public health priority” because there is still no effective therapeutic strategy in the treatment of this disease [6]. The lack of effectiveness in the application of currently existing pharmacological approaches and the complex pathogenesis of the disease, formed by many factors, dictates new requirements for the development of medicines for the scientific community, primarily based on the paradigm of creating multi-targeted agents [60,61,62,63,64].

In recent years, the relationship between HDAC6 and Alzheimer’s disease has attracted considerable attention from researchers. It was found that an increased level of HDAC6 is registered in the brains of patients and animals modelling this disorder [65,66,67,68,69,70]. In the work of Bai et al. [71], tomographic examination of [18F]PB118, a radioligand with impeccable penetration into the brain and high specificity for HDAC6, demonstrated a significantly increased level of radioactivity in the brain of 5xFAD mice compared with wild animals. Such an increase in HDAC6 content correlates with a high content of hyperphosphorylated tau protein [72,73,74,75] and a reduced level of acetylated α-tubulin [76], as well as with neuropathological changes caused by β-amyloid [77]. All this eventually leads to the emergence and progression of neuronal dysfunction.

Although the information that HDAC6 is involved in the etiopathogenesis of Alzheimer’s disease has appeared relatively recently and little is known about its pathomechanisms, the data discovered to date allow us to consider this regulatory protein as a promising therapeutic target for the treatment of neurodegenerative disorders. Thus, pharmacotherapy with HDAC6 inhibitors in animals simulating Alzheimer’s disease has demonstrated significant therapeutic potential due to the modulation of key links in the pathogenesis of this disease [78,79]. It is noteworthy that to date, the therapeutic potential in the treatment of neurodegenerative diseases has already been demonstrated for a number of HDAC6 inhibitors in a series of preclinical studies [80,81,82,83,84].

We synthesized a number of monoterpene-derived hydroxamic acids of two structural types, with the first one consisting of compounds lacking a linker fragment between the hydroxamate group and the monoterpene moiety. Conversely, substances of the second type were designed using a classical approach to HDAC inhibitors, consisting of monoterpene scaffolds as a Cap-group, aliphatic (hexa/heptamenthylene) or aromatic (para-substituted cynnamic acid derivatives) linkers and the hydroxamate fragment as a Zn-binding group.

All the compounds synthesized in our work were evaluated for their inhibitory activity against histone deacetylase 6. It was found that among a number of hydroxamic acids based on monoterpenoids, the presence of a linker had a great effect on the inhibitory activity of HDAC6, while the methylene linker motif had the most pronounced advantage. It is interesting to note that hydroxamic acids which do not have a linker part in their structure did not demonstrate an inhibitory ability against HDAC6, which emphasizes the importance of having a linker molecule in the structure that allows the hydroxamate group to successfully interact with the catalytic domain of histone deacetylases and chelate zinc ions.

For hydroxamic acids with high HDAC6-inhibiting activity, we also conducted docking analyses against the crystal structure of HDAC6 (PDB: 5EDU), where the features of the interaction of compounds with the sixth isoform of human HDAC, most commonly associated with Alzheimer’s disease, were revealed. Additionally, our analysis demonstrated a strong correlation between the results obtained in an in vitro study of HDAC6-inhibitory activity of hydroxamic acids and the indicators of docking scores.

The focus on the use of hydroxamic acids as templates for the creation of drugs aimed at the pharmacological correction of neurodegenerative disorders is due to the wide range of potential neuroprotective effects found for compounds of this class [85], and is not limited only to HDAC-inhibitory properties. One of the possible mechanisms may be the manifestation of an antioxidant effect, the strengthening of which can be achieved by introducing additional pharmacophore fragments into the molecule [86,87].

Oxidative stress plays an important role in the pathogenesis of neurodegenerative disorders such as Alzheimer’s disease [88,89,90]. The hyperproduction and accumulation of free radicals observed in this pathological condition lead to damage to cells and molecules of lipids, proteins and nucleic acids [91]. Antiradical properties in the context of the search for pharmacological agents aimed at correcting neurodegenerative disorders correlate with improved cognitive function and neuronal survival. In this regard, considerable attention is paid to antioxidant therapy, which is considered as a promising approach to slow the progression of neurodegeneration in Alzheimer’s disease [92,93].

In our work, the study of antiradical properties was carried out using methods for assessing the antioxidant activity of chemical objects, in particular, when searching for potential therapeutic agents involved in maintaining the redox balance in the body and treating pathologies associated with the action of free radicals-DPPH and ORAC tests. It was found that the greatest DPPH radical scavenging activity was hydroxamic acid with a (+)-α-pinene scaffold (+)-**19**, and hydroxamic acids with aromatic linker-**35a**, **35b**, **35c**, **38a**, **38b** and **38c**. When analyzing the ability of hydroxamic acids to absorb peroxyl radicals generated during the thermal decomposition of their source, AAPH, the ability to modulate the redox potential using this model was found for most compounds. It is important to note that the compounds with the most pronounced antiradical activity in the DPPH test had the highest oxygen radical absorbing ability, the values of which corresponded to or exceeded those of the well-known standard antioxidant trolox in a similar concentration. This evidently indicates the presence of a contribution of an aromatic linker based on para-substituted cinnamic acids to the antiradical properties of the compounds under study. 

Since the beginning of the last century, the concept of the amyloid origin of Alzheimer’s disease has been a defining direction in the development of potential therapeutic agents [94,95,96]. Due to the fact that the production and accumulation of pathological forms of β-amyloid are inevitable with the progression of Alzheimer’s disease, targeting the clearance of Aβ in the brain is considered as a promising strategy for the treatment of this disease.

To date, a large number of inhibitors of this process have been identified, belonging to compounds of various chemical classes [97,98,99,100]. Such molecules have their positive effect due to modulation by the conformation of amyloid peptides, stabilization of non-pathological forms of β-amyloid, switching aggregation towards non-toxic peptides, etc. Hydroxamate ligands can also be considered as β-amyloid peptide-binding molecules [58,101]. In particular, in the work of Yadav et al. [102], the protective effect of SH-SY5Y neuroblastoma cells against metal-induced β-amyloid aggregation and neuronal toxicity has been demonstrated. Intraperitoneal injections of deferoxamine also significantly inhibited the aberrant genesis of β-amyloid peptides, reversing spatial learning and memory impairments in APP/PS1 transgenic mice [103,104,105]. Similar properties have been demonstrated for valproic acid, which enhances the excretion of β-amyloid peptides in the brain of transgenic mice simulating Alzheimer’s disease [106].

In addition, to date, convincing evidence has been presented indicating a direct relationship between the aberrant activity of HDAC6 and the level of pathological forms of β-amyloid deposits in a model of transgenic mice with classical amyloid pathology [71]. A heterologous distribution of HDAC6-specific radioligand [18F]PB118 was found with the most pronounced changes in the cerebral cortex and hippocampus similar to the distribution of amyloid deposits. This association of HDAC6 with amyloid pathology may indicate that the use of agents targeting this enzyme may lead to a more effective reduction of β-amyloid plaques and soluble toxic Aβ-oligomers in the brain of patients with Alzheimer’s disease.

In this regard, we investigated the effect of hydroxamic acids on the kinetics of the aggregation process of the pathological form of β-amyloid peptide 1-42 by 48-h registration of the fluorescence of thioflavin T. Thioflavin T is a widely used fluorescent probe that allows the monitoring of the aggregation of β-amyloid [107,108,109].

Among hydroxamic acids, 7 out of 17 compounds demonstrated the ability to suppress the aggregation of the β-amyloid peptide. It is interesting to note that the most pronounced effect was found for compounds with an aromatic linker based on para-substituted cinnamic acids and having a monocyclic para-mentane backbone as a Cap group. Thus, **35a**, **38a**, **35b** and **38b** sharply reduced the fluorescence of thioflavin T, demonstrating a short phase of elongation of aggregates and thereby confirming their inhibitory effect against Aβ_1-42_ (up to 75%). The results obtained suggest an equivalent contribution of the nature of both the linker region and the Cap group to the anti-aggregational properties of compounds. This is due to the fact that because of **30** and **31** having similar Cap groups, as well as **35c** and **38c** with similar linker structures, no activity was detected, or it did not exceed 40%. Thus, the obtained results clearly demonstrated the role of synthesized hydroxamic acids in preventing the conversion of monomers of the pathological form of β-amyloid peptide 1-42 into its toxic aggregates.

The affinity of the synthesized compounds for the Aβ_1-42_ monomer was evaluated through molecular docking across the entire surface of the peptide. It has been demonstrated that the investigated compounds readily bind to both identified monomer binding pockets with varying degrees of preference, which may provide insight into a potential mechanism for inhibiting the aggregation of β-amyloid peptide monomers by these compounds.

The key stage of any study of a promising pharmacological substance considered for the treatment of a particular disease is to conduct experiments on model animals [110,111]. Such in vivo tests are an important tool for confirming the results obtained in in vitro experiments, as well as the possibility of extrapolating data from animals to humans. The approach of using transgenic animals is particularly promising due to more precise manipulation of key parameters of the disease [112,113,114,115]. In our work, based on the data of the in vitro screening of the biological activity of synthesized hydroxamic acids, hydroxamic acid with an aromatic linker and a Cap group containing the (-)-perillyl fragment **35a** was selected as a compound-leader. This compound demonstrated the most promising profile of biological activity, which was expressed in the manifestation of pronounced HDAC6-inhibiting properties and moderate antiradical activity, as well as an excellent ability to prevent the aggregation of the pathological form of β-amyloid peptide 1-42 into a toxic form. An analysis of the presence of possible toxic effects of **35a** on the cell model, expressed in cell death under the action of the substance, allowed us to continue further in vivo studies due to the lack of influence on the survival of the SH-SY5Y cell line.

It is known that the occurrence and progression of cognitive dysfunctions in Alzheimer’s disease is primarily associated with pathological changes observed in the hippocampal formation [116,117,118,119,120]. This is due to the fact that this area is one of the most sensitive to pathological cascades involved in the pathogenesis of this disease. In this regard, to test the assumption of the ability of hydroxamic acid **35a** to improve cognitive deficits of 13-month-old 5xFAD mice due to the multi-target properties found in in vitro experiments, spatial learning and memory were investigated using the Morris Water Maze test. This method is a universal tool for solving the task of analyzing hippocampus-dependent memory, since it is the gold standard for checking functions in this particular area of the brain [121,122].

In our work, similar to previous reports by other authors [123,124], we confirmed that transgenic mice of the 5xFAD line demonstrate a significant deterioration in the ability of spatial learning and memory formation, assessed using the Morris Water Maze test, compared with clinically healthy C57Bl6/j mice. In turn, the daily administration of hydroxamic acid **35a** at a dose of 15 mg/kg significantly weakened cognitive impairment, striving for the level of control wild-type animals. 

To confirm the neuroprotective effect of hydroxamic acid demonstrated in in vivo testing, at the end of animal experiments, we took brain samples in which we examined the level of oxidative stress, the functioning of the mitochondrial respiratory chain and the content of β-amyloid deposits.

Oxidative stress was assessed by measuring the levels of malondialdehyde, a key marker of oxidative stress [125,126]. The 5xFAD mice had significantly higher levels of MDA than the control group animals, which suggests higher levels of free radicals and damage to neuronal cells. Similar results were obtained in the works of other authors, where 5xFAD mice also suffered from damage caused by oxidative stress [127]. Interestingly, intraperitoneal administration of hydroxamic acid **35a** at a dose of 15 mg/kg reduced this indicator, striving for the level of control samples. Obviously, the results obtained indicate that **35a** is able to reduce oxidative damage in the brain of 5xFAD transgenic mice, due to the antiradical properties demonstrated for it in in vitro experiments.

It is known that as a result of free radical reactions, irreversible damage to mitochondria occurs first [128,129]. Such a violation of mitochondrial bioenergetics leads to anomalies in electron transfer reactions and proton leakage, which also stimulate the overproduction of free radicals. Due to the fact that the study of brain samples of transgenic mice demonstrated violations in the oxidative status for them, we additionally studied the respiratory ability of the synaptosomal-mitochondrial (p2) fraction to determine the possible effect of the leader compound **35a** on the activity of electron transport chain complexes.

It was found that in the samples obtained from native 5xFAD mice, in the presence of glutamate/malate, succinate and ascorbate/TMPD (substrates I, II and IV complexes), oxygen consumption by organelles was significantly reduced. This may indicate that the mutations observed in these animals block NADH-dehydrogenase, succinate dehydrogenase and cytochrome-C oxidase complexes, which in turn leads to the hyperproduction of reactive oxygen species. This is consistent with previous studies, where it has been repeatedly reported that both in patients and in model animals, the mitochondria of the brain are seriously damaged, in particular, in disorders of oxidative phosphorylation, hyperproduction of reactive oxygen species and the formation of transitional pores of mitochondrial permeability [130,131,132]. Interestingly, mitochondria obtained from mice from the 5xFAD + **35a** group were able to bypass the inhibition of II and IV mitochondrial complexes and normalize mitochondrial respiration.

We also found that 13-month-old transgenic male mice treated by hydroxamic acid **35a** had a lower amount of β-amyloid deposits in all brain regions studied, including hippocampus, thalamus and cortex zones. A significant number of studies prove that the increased content of this peptide directly correlates with cognitive deficiency [133,134,135], which, obviously, can explain the neuroprotective effect of the lead compound in in vivo experiments.

## 4. Materials and Methods

### 4.1. Chemistry

All chemicals were purchased from commercial sources (Sigma Aldrich (St. Louis, MO, USA), Acros Organics (Geel, Belgium)) and used without further purification. ^1^H and ^13^C NMR spectra were recorded on a Bruker AV-300 spectrometer (Bruker Corporation, Billerica, MA, USA) (300.13 MHz and 75.46 MHz, respectively), Bruker AV-400 (Bruker Corporation, Billerica, MA, USA) (400.13 MHz and 100.61 MHz), Bruker DRX-500 (Bruker Corporation, Billerica, MA, USA) (500.13 MHz and 125.76 MHz). Mass spectra (70 eV) were recorded on a DFS Thermo Scientific high-resolution mass spectrometer. A PolAAr 3005 polarimeter (Optical Activity, Ramsey, UK) was used to measure optical rotations [α]_D_. Melting points were measured on a Mettler Toledo FP900 Thermosystem apparatus (Mettler Toledo, Cornellà de Llobregat, Spain). Elemental analyses were performed on EA-3000 elemental analyzer. Merck silica gel (Merck, Darmstadt, Germany, 63–200 µm) was used for column chromatography. Spectral and analytical measurements were carried out at the Multi-Access Chemical Service Center of Siberian Branch of Russian Academy of Sciences (SB RAS) (Supplementary Materials).

*Synthesis of methyl 7-hydroxy-3,7-dimethyloctanoate **4***

A mixture of 7-hydroxycitronellal **3** (5.15 g; 29.94 mmol), oxone (9.30 g; 30.24 mmol) in 40 mL of MeOH was stirred at room temperature for 7 days. After completion of the reaction, the solvent was evaporated under reduced pressure, water was added and the product was extracted with EtOAc. The organic phase was dried over sodium sulfate. The product was isolated as a colorless liquid (5.02 g; 83%). Spectroscopic data were consistent with that previously reported [136].

*Synthesis of N,7-dihydroxy-3,7-dimethyloctanamide **5***

Methyl 7-hydroxy-3,7-dimethyloctanoate **4** (1.21 g; 6.0 mmol, 1 eq), NH_2_OH*HCl (1.25 g; 18.0 mmol; 3 eq), KOH (2.02 g; 36.0 mmol; 6 eq) and MeOH (15 mL) were added to a vessel, and the last one was sealed. The reaction mixture was stirred at 80 °C for 1 h. A total of 3M HCl was added to the mixture and MeOH was evaporated. The residue was dissolved in water and the product was extracted several times with EtOAc. The combined organic layer was dried over Na_2_SO_4_ and evaporated; the product was purified by crystallization from EtOAc. The product was isolated as a white solid (0.39 g; 32%). ^1^H NMR (300 MHz, DMSO-d_6_) δ 0.82 (d, J = 6.2 Hz, 3H), 0.98–1.11 (m, 7H), 1.13–1.40 (m, 5H), 1.64–2.00 (m, 3H), 4.14 (s, 1H), 8.71 (s, 1H), 10.36 (s, 1H). ^13^C NMR (101 MHz, DMSO-d_6_) δ 19.5, 21.4, 29.3, 29.4, 30.0, 37.0, 40.1, 43.9, 69.0, 168.7. HRMS: *m*/*z* 188.1277 (M-CH_3_^+^ C_9_H_18_O_3_N^+^, calc. 188.1281).

*Synthesis of 3,7-dimethyloctanoic acid **7***

A mixture containing 3,7-dimethyloctan-1-ol (1.00 g; 6.33 mmol), KMnO_4_ (3.00 g; 18.99 mmol), TBAB (1.10 g; 3.41 mmol) in 25 mL of water, 32 mL of DCE and 10 mL of acetic acid was refluxed for 5 h. The mixture was cooled to room temperature and HCl (conc.) was added until pH~1. Na_2_SO_3_ was slowly added while stirring until the mixture turned colorless. The mixture was diluted with water, the organic phase was separated, and the product was extracted with CH_2_Cl_2_. The combined organic phase was successively washed with 10% HCl (aq.), water and brine and was dried over sodium sulfate. The product was isolated as a colorless liquid (0.93 g; 85%). Spectroscopic data were consistent with that previously reported [137].

*Synthesis of campholenic acid **12***

Compounds (+)-**12** and (-)-**12** were obtained starting from (1S)-(+)-10-camphorsulfonic acid and (1R)-(-)-10-camphorsulfonic acid as described in [41]. Spectroscopic data were consistent with that previously reported [138].

*Synthesis of (-)-perillic acid **15***

Compound **15** was obtained as described in [42]. Spectroscopic data were consistent with that previously reported [139].

*Synthesis of (+)-myrtenal (+)-**17***

Compound (+)-**17** was obtained as described in [140]. Spectroscopic data were consistent with that previously reported [140].

*Synthesis of myrtenic acid **18***

Compounds (-)-**18** and (+)-**18** were obtained as described in [42]. Spectroscopic data were consistent with that previously reported [141].

*Synthesis of **8, 10, 13, 16** and **19***

NH_2_OH × HCl (1.67 g, 24.0 mmol) and K_2_CO_3_ (3.31 g; 24.0 mmol) were dissolved in water (20 mL), and ethyl acetate (20 mL) was added. After the resulting mixture was cooled to 0 °C, 10 mL of the ethyl acetate solution of the corresponding acyl chloride (12 mmol) was added dropwise and the reaction mixture was stirred at room temperature overnight. Layers were separated and the aqueous layer was extracted with EtOAc (2 × 30 mL); the combined organic layer was consequently washed with water (2 × 30 mL) and brine (2 × 15 mL), and it was dried over Na_2_SO_4_. The solvent was evaporated and the product was isolated by column chromatography with a gradient of EtOAc in hexane.

*N-Hydroxy-3,7-dimethyloctanamide **8***

White solid; yield of 25%.

^1^H NMR (300 MHz, DMSO-d_6_) δ 0.82 (d, J = 6.6 Hz, 3H), 0.84 (d, J = 6.6 Hz, 6H), 1.00–1.16 (m, 3H), 1.16–1.32 (m, 3H), 1.42–1.57 (m, 1H), 1.67–1.97 (m, 3H), 8.68 (s, 1H), 10.33 (s, 1H). ^13^C NMR (75 MHz, DMSO-d_6_) δ 19.4, 22.5, 22.6, 24.1, 27.4, 29.8, 36.4, 38.7, 40.0, 168.5. HRMS: *m*/*z* 186.1486 (M-H^+^ C_10_H_20_O_2_N^+^, calc. 186.1489).

*N-Hydroxy-3,7-dimethyloct-6-enamide **10***

Yellow solid; yield of 40%.

^1^H NMR (300 MHz, DMSO-d_6_) δ 0.83 (d, J = 6.2 Hz, 3H), 1.00–1.18 (m, 1H), 1.19–1.37 (m, 2H), 1.55 (s, 3H), 1.63 (s, 3H), 1.68–2.02 (m, 4H), 5.00–5.12 (m, 1H), 8.71 (s, 1H), 10.36 (s, 1H). ^13^C NMR (75 MHz, DMSO-d_6_) δ 17.6, 19.3, 25.0, 25.6, 29.6, 36.4, 39.9, 124.5, 130.8, 168.6. HRMS: *m*/*z* 185.1411 (M^+^ C_10_H_19_O_2_N^+^, calc. 185.1410).

*(S)-N-Hydroxy-2-(2,2,3-trimethylcyclopent-3-en-1-yl)acetamide (+)-**13***

Yellow solid; yield of 70%.

^1^H NMR (400 MHz, DMSO-d_6_) δ 0.71 (s, 3H), 0.94 (s, 3H), 1.55–1.58 (m, 3H), 1.76–1.92 (m, 2H), 2.01–2.29 (m, 3H), 5.17–5.22 (m, 1H), 8.70 (s, 1H), 10.38 (s, 1H). ^13^C NMR (101 MHz, DMSO-d_6_) δ 12.8, 19.9, 25.5, 33.5, 35.2, 46.6, 46.7, 121.8, 147.9, 169.2. HRMS: *m*/*z* 183.1252 (M^+^ C_10_H_17_O_2_N^+^, calc. 183.1254). ${\left[\alpha \right]}_{D}^{26}$ = +3.1 (c 0.8 in MeOH).

*(R)-N-Hydroxy-2-(2,2,3-trimethylcyclopent-3-en-1-yl)acetamide (-)-**13***

Yellow solid; yield of 39%.

NMR spectra were identical to (+)-**13**. HRMS: *m*/*z* 183.1257 (M^+^ C_10_H_17_O_2_N^+^, calc. 183.1254). ${\left[\alpha \right]}_{D}^{26}$ = −1.0 (c 1.0 in MeOH).

*(S)-N-Hydroxy-4-(prop-1-en-2-yl)cyclohex-1-enecarboxamide **16***

White solid; yield of 19%.

^1^H NMR (400 MHz, DMSO-d_6_) δ 1.30–1.44 (m, 1H), 1.71 (s, 3H), 1.74–1.84 (m, 1H), 1.91–2.04 (m, 1H), 2.04–2.36 (m, 4H), 4.72 (d, J = 5.7 Hz, 2H), 6.40–6.46 (м, 1H), 8.69 (s, 1H), 10.53 (s, 1H) ^13^C NMR (101 MHz, DMSO-d_6_) δ 165.5, 148.7, 131.2, 131.0, 109.2, 39.6, 30.0, 26.6, 24.2, 20.6. HRMS: *m*/*z* 181.1101 (M^+^ C_10_H_15_O_2_N^+^, calc. 181.1097). ${\left[\alpha \right]}_{D}^{26}$ = −85.6 (c 0.5 in MeOH).

*(1R,5S)-N-Hydroxy-6,6-dimethylbicyclo [3.1.1]hept-2-ene-2-carboxamide (-)-**19***

Yellow solid; yield of 38%.

^1^H NMR (400 MHz, DMSO-d_6_) δ 0.74 (s, 3H), 1.00 (d, J = 8.8 Hz, 1H), 1.27 (s, 3H), 2.06 (m, 1H), 2.20–2.43 (m, 3H), 2.55 (t, J = 5.7, 1H), 6.25 (m, 1H), 8.70 (s, 1H), 10.60 (s, 1H). ^13^C NMR (126 MHz, DMSO-d_6_) δ 20.8, 25.8, 30.9, 31.3, 37.3, 40.0, 41.0, 127.8, 141.1, 164.6. HRMS: *m*/*z* 181.1096 (M^+^ C_10_H_15_O_2_N^+^, calc. 181.1097). ${\left[\alpha \right]}_{D}^{26}$ = −49.0 (c 0.5 in MeOH).

*(1S,5R)-N-Hydroxy-6,6-dimethylbicyclo [3.1.1]hept-2-ene-2-carboxamide (+)-**19***

Yellow solid; yield of 44%.

NMR spectra were identical to (-)-**19.** HRMS: *m*/*z* 181.1099 (M^+^ C_10_H_15_O_2_N^+^, calc. 181.1097). ${\left[\alpha \right]}_{D}^{26}$ = +39.0 (c 0.9 in MeOH).

*Synthesis of (S)-2-((4-(prop-1-en-2-yl)cyclohex-1-en-1-yl)methyl)isoindoline-1,3-dione **21***

To a mixture of PPh_3_ (2.9 g; 11.0 mmol), phthalimide (1.6 g; 11.0 mmol) and perillyl alcohol (1.5 g; 10.0 mmol) in 30 mL of dry THF that was cooled by 0 °C, DIAD (2.2 g; 11.0 mmol) was added. The mixture was stirred at room temperature overnight and then the solvent was removed using rotary evaporator. The product was isolated by column chromatography (eluent-hexane/EtOAc) as a white solid (2.0 g; 72%). Spectroscopic data were consistent with that previously reported [142].

*Synthesis of 1-(bromomethyl)-4-isopropylbenzene **24***

To a solution of a cumyl alcohol (5.0 g; 33.3 mmol) in 30 mL of anhydrous toluene cooled to 0 °C, 1.2 mL (12.6 mmol) of PBr_3_ was added. The solution was stirred at room temperature overnight. After that, a saturated solution of NaHCO_3_ was poured into the reaction mixture, and the product was extracted with Et_2_O. The combined organic phase was washed with brine and dried over Na_2_SO_4_. The crude product (6.8 g; 95%) was used in the next step without purification. Spectroscopic data were consistent with that previously reported [143].

*Synthesis of 2-(4-isopropylbenzyl)isoindoline-1,3-dione **25***

A mixture containing bromide **24** (3.0 g; 14.1 mmol) and potassium phthalimide (2.7 g; 14.6 mmol) in 20 mL of DMF was stirred at 60 °C for several hours until the full conversion of starting materials (control by gas chromatography). After that, the reaction mixture was left to cool down to room temperature and water was added. The product was extracted with EtOAc, and the organic phase was subsequently washed with water and brine and dried over Na_2_SO_4_. The product was purified by column chromatography (eluent-hexane/EtOAc) and isolated as a white solid (3.6 g; 92%). Spectroscopic data were consistent with that previously reported [144].

*Synthesis of amines **22** and **26***

A solution of appropriate N-alkylphthalimide (1.8 mmol) and 0.25 mL of ethylenediamine in MeOH (6 mL) was refluxed for several hours until the full completion of the starting material (controlled by gas chromatography). The reaction mixture was cooled to room temperature and the solvent was evaporated under reduced pressure. Hexane was added to the crude material, a precipitate was separated and the organic phase was washed with water and brine and dried over Na_2_SO_4_. The products were isolated as a colorless liquid (74% for **22** and 60% for **26**). Spectroscopic data were consistent with that previously reported (**22**) [142], (**26**) [145].

*Synthesis of myrtanylamine **27***

To a stirred solution of β-pinene (6.8 g; 50 mmol) in 8 mL of dry THF under an ice-water bath, BH_3_ × Me_2_S (1.6 mL; 16.7 mmol) was slowly added in an Ar atmosphere. The solution was stirred at room temperature overnight. Hydroxylamine-O-sulfonic acid (4.2 g; 36.8 mmol) was then added and the reaction mixture was refluxed for 3 h. After that, the reaction mixture was allowed to cool to room temperature, a diluted solution of hydrochloric acid was added, and the acidified solution was extracted with Et_2_O. The water phase was neutralized with NaOH until pH 10–12, and the product was extracted with Et_2_O. The combined organic phase was washed with brine and dried over Na_2_SO_4_. A yield of the product was 3.9 g (50%). Spectroscopic data were consistent with that previously reported [146].

*Synthesis of oxonane-2,9-dione **28***

Suberic acid (5.0 g; 28.7 mmol) was refluxed in 10 mL of acetic anhydride for 3 h. The solvent was evaporated in vacuo to provide 4.4 g (98%) of the crude material, which was used in the next step without purification. Spectroscopic data were consistent with that previously reported [147].

*Synthesis of oxocane-2,8-dione **29***

Azelaic acid (10.0 g; 53.2 mmol) was refluxed in 30 mL of acetic anhydride for 6 h. The solvent was evaporated in vacuo to provide 9.0 g (99%) of the crude material, which was used in the next step without purification. Spectroscopic data were consistent with that previously reported [147].

*Synthesis of (S)-N1-Hydroxy-N8-((4-(prop-1-en-2-yl)cyclohex-1-en-1-yl)methyl)octanediamide **30***

Amine **22** (0.364 g; 2.43 mmol) was slowly added to a solution of suberic anhydride (0.403 g; 2.58 mmol) in dry THF (25 mL) at 0 °C with vigorous stirring; the resulting mixture was stirred overnight at room temperature. The solution was filtered and evaporated, leaving a corresponding acid, which was used further without purification. A solution of amido acid in dry THF (25 mL) was treated with ethyl chloroformate (0.434 mL; 4.56 mmol) and triethylamine (0.689 mL; 4.94 mmol) at 0 °C and consequently stirred at room temperature for 30 min and filtered. At the same time, hydroxylamine hydrochloride (0.668 g; 9.59 mmol) and potassium hydroxide (0.537 g; 9.57 mmol) solutions in methanol (5 mL each) were prepared. The methanol solution resulting from their mixing with the following filtration was added to the amido acid solution in THF; the reaction mixture was stirred overnight, filtered, evaporated, suspended in chloroform, filtered again, evaporated, dissolved in 60 mL of EtOAc, washed consequently with 5% NaOH and brine to isolate the unreacted amido acid, dried over Na_2_SO_4_ and evaporated in vacuo. The crude product was purified by column chromatography on silica gel (eluent-CHCl_3_/MeOH) to reveal the titled compound as a white solid (0.27 g; 35%). Mp 113.5–116 °C; ^1^H NMR (400 MHz, DMSO-d_6_) δ 1.16–1.29 (m, 4H), 1.30–1.42 (m, 1H), 1.41–1.53 (m, 4H), 1.68–1.71 (m, 3H), 1.71–1.79 (m, 1H), 1.80–1.88 (m, 1H), 1.87–1.98 (m, 4H), 2.01–2.11 (m, 4H), 3.56 (d, J = 5.8 Hz, 2H), 4.67–4.72 (m, 2H), 5.45–5.50 (m, 1H), 7.83 (t, J = 5.8 Hz, 1H), 8.68 (s, 1H), 10.32 (s, 1H). ^13^C NMR (151 MHz, DMSO-d_6_) δ 171.9, 169.0, 149.2, 134.8, 120.5, 108.8, 43.7, 40.5, 35.3, 32.2, 29.8, 28.4, 28.4, 27.0, 26.5, 25.3, 25.0, 20.6. HRMS: *m*/*z* 322.2246 (M^+^ C_18_H_30_O_3_N_2_^+^, calc. 322.2251). ${\left[\alpha \right]}_{D}^{26}$ = −48.4 (c 0.45 in MeOH).

*Synthesis of (S)-N1-hydroxy-N9-((4-(prop-1-en-2-yl)cyclohex-1-en-1-yl)methyl)nonanediamide **31***

Amine **22** (0.30 g; 1.99 mmol) was added to a solution of azelaic anhydride (0.31 g, 1.82 mmol) in 20 mL of dry THF cooled to 0 °C. The resulting solution was stirred at room temperature for 24 h and cooled to ~−20 °C. The precipitate formed was filtered out; the filtrate was evaporated under a reduced pressure. The solid was dissolved in 10 mL of dry THF, and Et_3_N (0.3 mL; 2.15 mmol) and ethyl chloroformate (0.19 mL; 2.02 mmol) were added to the resulting solution while cooling. The mixture was stirred at room temperature for 1 h and the solid precipitated was filtered out. The filtrate was added to a solution of hydroxylamine in MeOH, which was prepared as follows: to a cooled solution of KOH (0.23 g; 4.11 mmol) in 5 mL of MeOH a solution of NH_2_OH * HCl (0.29 g; 4.17 mmol) in MeOH (5 mL) was added. The mixture was stirred at room temperature for 30 min and the solid formed was removed. The resulting mixture was stirred at room temperature overnight. The precipitate was removed and the solvent was evaporated in vacuo. The crude product was purified by column chromatography on silica gel (eluent-CHCl_3_/MeOH) to reveal the titled compound as a white solid (0.29 g; 44%). Mp 110.8 °C; ^1^H NMR (400 MHz, DMSO-d_6_) δ 1.16–1.29 (m, 6H), 1.30–1.41 (m, 1H), 1.42–1.52 (m, 4H), 1.67–1.71 (m, 3H), 1.72–1.80 (m, 1H), 1.81–1.88 (m, 1H), 1.89–1.99 (m, 4H), 2.00–2.13 (m, 4H), 3.56 (d, J = 5.7 Hz, 2H), 4.69 (m, 2H), 5.48 (m, 1H), 7.83 (t, J = 5.9 Hz, 1H), 8.65 (s, 1H), 10.32 (s, 1H). ^13^C NMR (101 MHz, DMSO-d_6_) δ 171.9, 169.1, 149.2, 134.9, 120.5, 108.9, 43.7, 40.5, 35.3, 32.2, 29.8, 28.6, 28.5, 28.5, 27.1, 26.5, 25.3, 25.1, 20.6. HRMS: *m*/*z* 336.2407 (M^+^ C_19_H_32_O_3_N_2_^+^, calc. 336.2410). ${\left[\alpha \right]}_{D}^{23}$ = −33.1 (c 0.32 in MeOH).

*Synthesis of methyl 4-formylbenzoate **32***

Thionyl chloride (1.5 mL, 20.7 mmol) was added to a mixture of 4-formylbenzoic acid (1.0 g; 6.7 mmol) in methanol (15 mL) and cooled to 0 °C. The mixture was stirred overnight; then, the solvent was evaporated and an aqueous solution of HCl was added. The mixture was stirred for 6 h; the solid formed was filtered off, washed with water and dried. The product yield was 0.9 g (85%). The NMR spectra were consistent with previously reported data [148].

*Synthesis of (E)-3-(4-methoxycarbonylphenyl)prop-2-enoic acid **33***

A solution consisting of methyl 4-formylbenzoate **32** (0.5 g; 3.1 mmol), malonic acid (0.48 g; 4.58 mmol), and piperidine (0.25 mL) in pyridine (2.5 mL) was refluxed for 2 h. After being cooled to room temperature, the reaction mixture was poured into 1M HCl (30 mL). The precipitate was filtered, washed with water and acetonitrile, and dried to provide 0.57 g (91%) of (E)-3-(4-methoxycarbonylphenyl)prop-2-enoic acid. NMR data were in agreement with [147].

*Synthesis of (E)-4-(3-methoxy-3-oxoprop-1-en-1-yl)benzoic acid **36***

To a solution of 4-formylbenzoic acid (0.2 g; 1.3 mmol) and K_2_CO_3_ (0.6 g; 4.0 mmol), in 3 mL of water, trimethyl phosphonoacetate (0.3 g; 1.6 mmol) was added. The reaction mixture was stirred at room temperature for overnight before acidifying to pH 2. The resulting precipitate was filtered, washed with water and dried. The spectral data were identical to those described in the literature [149].

*Synthesis of esters **34a—c** and **37a—c***

T3P (50 wt.% solution in ethyl acetate; 10 mmol) was added to a mixture of (E)-3-(4-methoxycarbonylphenyl)prop-2-enoic acid (4.9 mmol), amine (5.3 mmol) and pyridine (1.3 mL) in ethyl acetate (2.7 mL). The mixture was stirred at 75 °C for 8–10 h and then a saturated solution of NaHCO_3_ was added. The precipitate formed was filtered, washed with water and dried.

*Methyl (S,E)-4-(3-oxo-3-(((4-(prop-1-en-2-yl)cyclohex-1-en-1-yl)methyl)amino)prop-1-en-1-yl)benzoate **34a***

White solid, yield of 73% and mp of 126.7 °C.

^1^H NMR (500 MHz, CDCl_3_) δ 1.34–1.51 (m, 1H), 1.67 (s, 3H), 1.73–1.82 (m, 1H), 1.83–1.94 (m, 1H), 1.95–2.17 (m, 4H), 3.78–3.98 (m, 5H), 4.66 (d, J = 15.0 Hz, 2H), 5.60 (brs, 1H), 6.14–6.30 (m, 1H), 6.55 (d, J = 15.8 Hz, 1H), 7.49 (d, J = 8.0 Hz, 2H), 7.62 (d, J = 15.8 Hz, 1H), 7.95 (d, J = 8.0 Hz, 2H). ^13^C NMR (126 MHz, CDCl_3_) δ 166.4, 165.3, 149.5, 139.6, 139.1, 133.8, 130.6, 129.9, 127.5, 123.1, 122.9, 108.6, 52.1, 45.2, 40.8, 30.3, 27.23, 26.9, 20.6. HRMS: *m*/*z* 339.1829 (M^+^ C_21_H_25_O_3_N^+^, calc. 339.1827). ${\left[\alpha \right]}_{D}^{26}$ = −47.3 (c 0.6 in CHCl_3_).

*Methyl (E)-4-(3-((4-isopropylbenzyl)amino)-3-oxoprop-1-en-1-yl)benzoate **34b***

White solid, yield of 80% and mp of 170.8–172.2 °C.

^1^H NMR (300 MHz, CDCl_3_) δ 1.21 (d, J = 6.9 Hz, 6H), 2.89 (h, J = 6.9 Hz, 1H), 3.89 (s, 3H), 4.52 (d, J = 5.6 Hz, 2H), 6.02 (t, J = 5.1 Hz, 1H), 6.46 (d, J = 15.6 Hz, 1H), 7.13–7.31 (m, 4H), 7.51 (d, J = 8.4 Hz, 2H), 7.66 (d, J = 15.6 Hz, 1H), 7.99 (d, J = 8.4 Hz, 2H). ^13^C NMR (126 MHz, CDCl_3_) δ 166.4, 165.1, 148.3, 139.8, 139.0, 135.2, 130.6, 129.9, 127.9, 127.5, 126.7, 122.8, 52.1, 43.6, 33.7, 23.8. HRMS: *m*/*z* 337.1669 (M^+^ C_21_H_23_O_3_N^+^, calc. 337.1673).

*Methyl 4-((E)-3-((((1S,2R,5S)-6,6-dimethylbicyclo[3.1.1]heptan-2-yl)methyl)amino)-3-oxoprop-1-en-1-yl)benzoate **34c***

White solid, yield of 45% and mp of 79.6–81.6 °C.

^1^H NMR (300 MHz, CDCl_3_) δ 0.87 (d, J = 9.6 Hz, 1H), 1.04 (s, 3H), 1.17 (s, 3H), 1.42–1.62 (m, 1H), 1.75–2.01 (m, 5H), 2.15–2.44 (m, 2H), 3.28–3.49 (m, 2H), 3.90 (s, 3H), 5.81 (t, J = 6.0 Hz, 1H), 6.46 (d, J = 15.6 Hz, 1H), 7.51 (d, J = 8.4 Hz, 2H), 7.61 (d, J = 15.6 Hz, 1H), 7.99 (d, J = 8.4 Hz, 2H). ^13^C NMR (126 MHz, CDCl_3_) δ 166.5, 165.2, 139.4, 139.1, 130.5, 129.9, 127.5, 123.1, 52.1, 45.3, 43.5, 41.2, 41.1, 38.6, 33.1, 27.8, 25.8, 23.1, 19.7. HRMS: *m*/*z* 341.1988 (M^+^ C_21_H_27_O_3_N^+^, calc. 341.1986). ${\left[\alpha \right]}_{D}^{26}$ = −2.9 (c 0.42 in CHCl_3_).

*Methyl (S,E)-3-(4-(((4-(prop-1-en-2-yl)cyclohex-1-en-1-yl)methyl)carbamoyl)phenyl)acrylate **37a***

White solid, yield of 60% and mp of 138.5 °C.

^1^H NMR (400 MHz, CDCl_3_) δ 1.41–1.55 (m, 1H), 1.71 (s, 3H), 1.78–1.88 (m, 1H), 1.89–2.00 (m, 1H), 2.02–2.22 (m, 4H), 3.80 (s, 3H), 3.88–4.07 (m, 2H), 4.70 (d, J = 8.3 Hz, 2H), 5.58–5.71 (m, 1H), 6.12 (t, J = 5.1 Hz, 1H), 6.48 (d, J = 16.0 Hz, 1H), 7.56 (d, J = 8.3 Hz, 2H), 7.68 (d, J = 16.0 Hz, 1H), 7.78 (d, J = 8.3 Hz, 2H). ^13^C NMR (126 MHz, CDCl_3_) δ 166.9, 166.5, 149.5, 143.4, 137.1, 135.7, 133.9, 128.0, 127.4, 122.9, 119.4, 108.6, 51.7, 45.5, 40.8, 30.3, 27.3, 26.9, 20.6. HRMS: *m*/*z* 339.1833 (M^+^ C_21_H_25_O_3_N^+^, calc. 339.1829). ${\left[\alpha \right]}_{D}^{25}$ = −42.7 (c 0.48 in CHCl_3_).

*Methyl (E)-3-(4-((4-isopropylbenzyl)carbamoyl)phenyl)acrylate **37b***

White solid, yield of 74% and mp of 159.3–161.2 °C.

^1^H NMR (300 MHz, CDCl_3_) δ 1.12–1.41 (m, 6H), 2.89 (h, J = 6.8 Hz, 1H), 3.79 (s, 3H), 4.60 (d, J = 5.5 Hz, 2H), 6.30–6.39 (m, 1H), 6.47 (d, J = 16.0 Hz, 1H), 7.13–7.41 (m, 4H), 7.55 (d, J = 8.2 Hz, 2H), 7.67 (d, J = 16.1 Hz, 1H), 7.78 (d, J = 8.2 Hz, 2H). ^13^C NMR (126 MHz, CDCl_3_) δ 166.9, 166.3, 148.4, 143.3, 137.1, 135.6, 135.1, 128.0, 127.9, 127.4, 126.7, 119.5, 51.7, 43.9, 33.7, 23.9. HRMS: *m*/*z* 337.1676 (M^+^ C_21_H_23_O_3_N^+^, calc. 337.1673).

*Methyl (E)-3-(4-((((1S,2R,5S)-6,6-dimethylbicyclo[3.1.1]heptan-2-yl)methyl)carbamoyl)phenyl)acrylate **37c***

White solid, yield of 72% and mp of 89.9–91.2 °C.

^1^H NMR (400 MHz, CDCl_3_) δ 0.89 (d, J = 9.7 Hz, 1H), 1.06 (s, 3H), 1.18 (s, 3H), 1.48–1.59 (m, 1H), 1.70 (s, 1H), 1.80–2.05 (m, 3H), 2.23–2.42 (m, 2H), 3.37–3.54 (m, 2H), 3.80 (s, 3H), 6.15 (t, J = 5.9 Hz, 1H), 6.47 (d, J = 16.1 Hz, 1H), 7.55 (d, J = 8.4 Hz, 2H), 7.67 (d, J = 16.1 Hz, 1H), 7.74 (d, J = 8.3 Hz, 2H). ^13^C NMR (126 MHz, CDCl_3_) δ 167.0, 166.6, 143.4, 136.9, 136.0, 127.9, 127.3, 119.3, 51.7, 45.5, 43.6, 41.2, 41.1, 38.6, 33.1, 27.8, 25.8, 23.0, 19.7. HRMS: *m*/*z* 341.1982 (M^+^ C_21_H_27_O_3_N^+^, calc. 341.1986). ${\left[\alpha \right]}_{D}^{25}$ = −3.7 (c 0.54 in CHCl_3_).

*Synthesis of hydroxamic acids **35a–c** and **38a–c***

KOH (0.6 g; 10.9 mmol) was added to a cooled suspension of NH_2_OH*HCl (0.5 g; 7.3 mmol) in 3 mL of MeOH. The mixture was stirred at room temperature for 30 min; the precipitate formed was filtered and the solution was added to a solution of an ester (0.3 mmol) in 1 mL of MeOH cooled by an ice-water bath. The mixture was stirred at the same temperature for 10 min and the solvent was evaporated under reduced pressure. Water was added to the residue and the solution formed was neutralized with an aqueous solution of HCl to pH 5–6. The precipitate was filtered, washed with water and dried. The product was recrystallized from EtOH.

*(S,E)-N-hydroxy-4-(3-oxo-3-(((4-(prop-1-en-2-yl)cyclohex-1-en-1-yl)methyl)amino)prop-1-en-1-yl)benzamide **35a***

White solid, yield of 66% and mp of 179.9 °C.

^1^H NMR (400 MHz, DMSO-d_6_) δ 1.30–1.47 (m, 1H), 1.69 (s, 3H), 1.73–1.81 (m, 1H), 1.81–1.93 (m, 1H), 1.95–2.15 (m, 4H), 3.72 (d, J = 5.6 Hz, 2H), 5.51–5.59 (m, 1H), 6.76 (d, J = 15.8 Hz, 1H), 7.46 (d, J = 15.8 Hz, 1H), 7.62 (d, J = 8.0 Hz, 2H), 7.78 (d, J = 8.0 Hz, 2H), 8.25 (t, J = 5.9 Hz, 1H), 9.11 (s, 1H), 11.28 (s, 1H). ^13^C NMR (101 MHz, DMSO-d_6_) δ 165.0, 164.0, 149.7, 138.2, 138.0, 135.0, 133.7, 127.9, 127.9, 124.2, 121.5, 109.4, 44.6, 41.0, 30.3, 27.5, 27.0, 21.1. HRMS: *m*/*z* 340.1781 (M^+^ C_20_H_24_O_3_N_2_^+^, calc. 340.1785). ${\left[\alpha \right]}_{D}^{25}$ = −28.9 (c 0.46 in MeOH).

*(E)-N-hydroxy-4-(3-((4-isopropylbenzyl)amino)-3-oxoprop-1-en-1-yl)benzamide **35b***

White solid, yield of 75% and mp of 187.4–189.7 °C.

^1^H NMR (400 MHz, DMSO-d_6_) δ 1.18 (d, J = 6.9 Hz, 6H), 2.85 (h, J = 6.9 Hz, 1H), 4.36 (d, J = 5.6 Hz, 2H), 6.77 (d, J = 15.8 Hz, 1H), 7.16–7.27 (m, 4H), 7.49 (d, J = 15.8 Hz, 1H), 7.63 (d, J = 8.0 Hz, 2H), 7.78 (d, J = 8.0 Hz, 2H), 8.66 (t, J = 5.6 Hz, 1H), 9.11 (s, 1H), 11.23 (s, 1H). ^13^C NMR (126 MHz, DMSO-d_6_) δ 164.6, 163.6, 147.1, 137.9, 137.5, 136.7, 133.3, 127.6, 127.5, 126.3, 123.7, 42.2, 33.2, 24.0. Anal. Calcd for C_20_H_22_N_2_O_3_: C, 70.99; H, 6.55; N, 8.28. Found: C, 70.81; H, 6.61; N, 8.10.

*4-((E)-3-((((1S,2R,5S)-6,6-dimethylbicyclo[3.1.1]heptan-2-yl)methyl)amino)-3-oxoprop-1-en-1-yl)-N-hydroxybenzamide **35c***

White solid, yield of 58% and mp of 139.5–141.4 °C.

^1^H NMR (300 MHz, DMSO-d_6_) δ 0.85 (d, J = 9.4 Hz, 1H), 1.03 (s, 3H), 1.16 (s, 3H), 1.38–1.57 (m, 1H), 1.73–2.03 (m, 5H), 2.16 (q, J = 8.1 Hz, 1H), 2.25–2.41 (m, 1H), 3.18 (t, J = 6.7 Hz, 2H), 6.70 (d, J = 15.8 Hz, 1H), 7.42 (d, J = 15.8 Hz, 1H), 7.62 (d, J = 7.9 Hz, 2H), 7.77 (d, J = 7.9 Hz, 2H), 8.14 (brs, 1H), 9.09 (s, 1H), 11.27 (s, 1H). ^13^C NMR (101 MHz, DMSO-d_6_) δ 164.6, 163.7, 137.6, 137.4, 133.2, 127.5, 127.4, 124.0, 44.3, 43.0, 40.8, 40.8, 38.3, 32.7, 27.8, 25.7, 22.9, 19.3. Anal. Calcd for C_20_H_26_N_2_O_3_: C, 70.15; H, 7.65; N, 8.18. Found: C, 69.94; H, 7.75; N, 8.11. ${\left[\alpha \right]}_{D}^{25}$ = −1.4 (c 0.42 in MeOH).

*(S,E)-4-(3-(hydroxyamino)-3-oxoprop-1-en-1-yl)-N-((4-(prop-1-en-2-yl)cyclohex-1-en-1-yl)methyl)benzamide **38a***

White solid, yield of 51% and mp of 167.2–167.6 °C.

^1^H NMR (500 MHz, DMSO-d_6_) δ 1.33–1.45 (m, 1H), 1.69 (s, 3H), 1.73–1.80 (m, 1H), 1.82–1.94 (m, 1H), 1.95–2.16 (m, 4H), 3.80 (d, J = 5.9 Hz, 2H), 4.69 (s, 2H), 5.54 (s, 1H), 6.55 (d, J = 15.8 Hz, 1H), 7.49 (d, J = 15.8 Hz, 1H), 7.64 (d, J = 8.0 Hz, 2H), 7.89 (d, J = 8.0 Hz, 2H), 8.64 (t, J = 5.9 Hz, 1H), 9.14 (s, 1H), 10.84 (s, 1H). ^13^C NMR (126 MHz, DMSO-d_6_) δ 165.5, 162.5, 149.3, 137.5, 137.4, 135.0, 134.7, 127.9, 127.4, 120.7, 120.6, 108.9, 44.4, 40.6, 29.9, 27.1, 26.6, 20.7. HRMS: *m*/*z* 340.1778 (M^+^ C_20_H_24_O_3_N_2_^+^, calc. 340.1781). ${\left[\alpha \right]}_{D}^{25}$ = −34.3 (c 0.5 in MeOH).

*(E)-4-(3-(hydroxyamino)-3-oxoprop-1-en-1-yl)-N-(4-isopropylbenzyl)benzamide **38b***

White solid, yield of 70% and mp of 160.3–164.9 °C.

^1^H NMR (500 MHz, DMSO-d_6_) δ 1.17 (d, J = 6.9 Hz, 6H), 2.84 (h, J = 6.9 Hz, 1H), 4.44 (d, J = 5.9 Hz, 2H), 6.56 (d, J = 15.8 Hz, 1H), 7.14–7.32 (m, 4H), 7.50 (d, J = 15.9 Hz, 1H), 7.66 (d, J = 8.0 Hz, 2H), 7.92 (d, J = 8.0 Hz, 2H), 9.07 (t, J = 5.9 Hz, 1H), 9.14 (s, 1H), 10.84 (s, 1H). ^13^C NMR (126 MHz, DMSO-d_6_) δ 165.56, 162.47, 146.96, 137.50, 137.42, 136.99, 134.83, 127.88, 127.40, 127.36, 126.21, 120.75, 42.46, 33.15, 23.98. HRMS: *m*/*z* 338.1626 (M^+^ C_20_H_22_O_3_N_2_^+^, calc. 338.1625).

*N-(((1S,2R,5S)-6,6-dimethylbicyclo[3.1.1]heptan-2-yl)methyl)-4-((E)-3-(hydroxyamino)-3-oxoprop-1-en-1-yl)benzamide **38c***

White solid, yield of 65% and mp of 108.6 °C.

^1^H NMR (500 MHz, DMSO-d_6_) δ 0.85 (d, J = 9.3 Hz, 1H), 1.06 (s, 3H), 1.17 (s, 3H), 1.45–1.57 (m, 1H), 1.77–1.99 (m, 6H), 2.31 (t, J = 7.9 Hz, 2H), 3.27 (t, J = 6.9 Hz, 2H), 6.54 (d, J = 15.8 Hz, 1H), 7.49 (d, J = 15.8 Hz, 1H), 7.63 (d, J = 7.9 Hz, 2H), 7.85 (d, J = 7.9 Hz, 2H), 8.47 (t, J = 5.8 Hz, 1H), 9.08 (s, 1H), 10.80 (s, 1H). ^13^C NMR (126 MHz, DMSO-d_6_) δ 165.9, 162.8, 137.7, 137.6, 135.5, 128.0, 127.6, 120.9, 45.0, 43.4, 41.1, 41.0, 38.6, 33.1, 28.1, 26.0, 23.2, 19.4. HRMS: *m*/*z* 342.1942 (M^+^ C_20_H_26_O_3_N_2_^+^, calc. 342.1938). ${\left[\alpha \right]}_{D}^{25}$ = −4.1 (c 1.1 in MeOH).

### 4.2. Fluorimetric Measurement of HDAC6 Activity

HDAC6 activity was determined using the fluorometric activity analysis kit FLUOR DE LYS^®®^HDAC (Enzo Life Sciences, Farmingdale, NY, USA) in accordance with the manufacturer’s instructions. Trichostatin A was used as a well-known HDAC inhibitor. Fluorescence was measured using a multifunctional tablet analyzer Cytation™3 (BioTek Instruments, Inc., Winooski, VT, USA) at λ_ex_ = 360 nm, λ_em_ = 460 nm.

### 4.3. In Vitro Antiradical Activities

The measurement of the antiradical activity of synthesized hydroxamic acids was carried out using the method described in [150]. The studied compounds were introduced into the wells of a 96-well black tablet (the final concentration was 100 microns), after which a freshly prepared solution of 2,2-diphenyl-1-picrylhydrazyl (Sigma Aldrich, St. Louis, Missouri, USA) was added. After the incubation time had elapsed, the DPPH radical-scavenging activity was measured using a multifunctional Cytation™3 tablet analyzer (BioTek Instruments, Inc., Winooski, VT, USA) at λ = 517 nm by the difference in the reduction of the DPPH radical peak between control samples containing an equivalent volume of solvent and samples with the substances under study.

The analysis of the absorption capacity of oxygen radicals was performed as described in [151], with some modifications. Trolox (Sigma Aldrich, St. Louis, MI, USA) was used as a standard antioxidant. The studied compounds were introduced into the holes of a 96-hole black tablet with a transparent bottom with Trolox (25 µL; the final concentration was 100 µM) and fluorescein (150 µL; the final concentration was 80 nM, Sigma Aldrich, St. Louis, MI, USA). The resulting mixture was shaken and incubated for 30 min at 37 °C. After a 30-min incubation period, a solution of 2,2’-azobis (2-amidinopropane) dihydrochloride (AAPH) (25 µL; final concentration of 12 mM, Sigma Aldrich, St. Louis, MI, USA) was quickly introduced into all wells of the tablet. The tablet was immediately placed in a multifunctional tablet analyzer Cytation™ 3 (BioTek Instruments, Inc., Winooski, VT, USA). The fluorescence intensity was recorded for 300 min at λ_ex_ = 480 nm, λ_em_ = 520 nm with an interval of every 5 min. In the same experiment, calibration solutions for the reference antioxidant Trolox were used to confirm the validity of the use of this method (0, 6.25, 12.5, 25, 50 and 100 µM).

### 4.4. Fluorimetric Measurement of Thioflavin T

Fluorescence analysis using Thioflavin T made it possible to monitor the aggregation process of the pathological form of β-amyloid peptide 1-42 (Sigma Aldrich, St. Louis, MI, USA). The experimental protocol was carried out similarly to that described in [58] with some modifications. The registration of changes in the fluorescence of thioflavin T (10 µM, Sigma Aldrich, St. Louis, MI, USA) was performed on a multifunctional tablet analyzer Cytation™3 (BioTek Instruments, Inc., Winooski, VT, USA) at λ_ex_ = 450 nm, λ_em_ = 480 nm. The fluorescence intensity of the solution without Aβ_1-42_ was subtracted from the fluorescence values of solutions containing protein, due to the need to subtract the background fluorescence.

### 4.5. Molecular Docking and ADME/Tox Evaluation

For the HDAC6 protein and β-amyloid, the corresponding structures were found in the Protein Data Bank (PDB)-5EDU and 1IYT. The structures were loaded and processed using the Protein Preparation Wizard subroutine of the Schrodinger Suite software package [152,153,154,155,156], with any missing loops and side chains restored. Preprocessing was performed using the Prime module [56,152,153], hydrogen bonds were optimized, non-key waters and other non-key small molecules were removed, and a limited minimization of the protein geometry was performed using the OPLS3e force field [157].

The ligand structures were prepared using the LigPrep [158] subroutine. The pharmacokinetic profile of the compounds was calculated using the QikProp subroutine [59]. Docking to the active center of the HDAC6 protein was performed using the Induced Fit Docking protocol [56,158,159,160,161,162,163], and the reference ligand was redocked to verify the selected method. The active center for Induced Fit Docking was declared as a cube centered in the geometric coordinate center of the crystallized ligand of trichostatin A. The cube edges corresponded to the possibility of docking ligands commensurate with the reference. The Prime module processed amino acids within a 5 Å radius from the ligand atoms according to the results of pre-docking in the Induced Fit algorithm. Post-processing by Glide docking was carried out using the Standard Precision protocol.

The RMSD of the reference ligand of the 5EDU structure was 0.6205, indicating that the reference pose is reproduced well and the method of docking to the active center of the HDAC6 protein is selected successfully.

During the work with the β-amyloid structure, the Glide software package [163,164,165,166] was used for the primary docking and determination of the active amino acids to which the compounds have the greatest affinity. Using the Receptor Grid Generation tool of the Glide software package, the largest possible receptor grid was created: the cube limiting the position of the geometric centers of ligands during docking had the maximum allowable size of 40 × 40 × 40 Å, centered in the geometric center of the peptide atoms’ coordinates; the size of the allowable ligands for docking was also set to a maximum value of 36 Å. In the resulting receptor grid, Glide docking was carried out using the Extra Precision protocol, in order to determine the binding domains of the peptide, in which the coordinates of the ligands obtained from the docking results would be clustered. Two peptide fragments were found to which the molecules demonstrated affinity: the N-terminus of the peptide and a bend closer to the C-terminus of the peptide. Compounds were docked according to both of these provisions using the Induced Fit Docking protocol.

The active centers for Induced Fit Docking were declared as cubes centered in the geometric center of the coordinates of amino acid atoms from 3 to 12 (C-end) and amino acids from 22 to 30 (fold near the N-end). The cube edges corresponded to the possibility of docking ligands with a radius of 20 Å. The Prime module processed amino acids within a 5 Å radius from the ligand atoms according to the results of pre-docking in the Induced Fit algorithm. Post-processing by Glide docking was carried out using the Standard Precision protocol.

The best docking poses obtained in this manner were introduced into the calculation of ΔG MM-GBSA using the Prime module, with the declaration of all amino acids within a 5 Å radius from the ligand atoms as “flexible” and undergoing optimization. The obtained binding ΔG values were used for statistical analysis using the Boltzmann distribution (assuming a temperature of 298 K) to determine the frequency of reproducing docking positions relative to each other for each molecule.

### 4.6. Cell Culture and Cell Viability Assay

The culture of human neuroblastoma cells (defined as SH-SY5Y) was cultured in DMEM with the addition of embryonic bovine serum (10%), 2 mM glutamine (DiaM, Moscow, Russia) and penicillin-streptomycin (100 U/Ml–100 µg/mL, PanEco, Moscow, Russia) in conditions of 5% CO_2_ in the air at 37 °C. Upon reaching 80% confluence, cells in the amount of 1 × 10^4^ were placed in 96-well transparent plates and incubated under the same conditions for 24 h. After the incubation time, the cells were exposed to a 24-h exposure to various concentrations of hydroxamic acids dissolved in dimethyl sulfoxide (range from 1 to 100 µM). An equivalent volume of solvent was used as a control (final DMSO content = 1%).

Cell survival analysis was performed using MTT test. To do this, 3-(4,5-dimethylthiazol-2-yl)-2,5-diphenyltetrazolium bromide (MTT, 5 mg/mL, DiaM, Moscow, Russia) was introduced into all wells and incubated at 37 °C for 2 h to restore metabolically active cells of the yellow tetrazolium salt MTT to purple formazane crystals. The resulting formazane was then re-suspended in 200 µL DMSO per well. The optical density was recorded using a multifunctional Cytation™3 flatbed analyzer (BioTek Instruments, Inc., Winooski, VT, USA) at λ = 530 nm.

### 4.7. Animals Grouping and Treatment

As part of the in vivo testing of the biological activity of the leader compound, a transgenic mouse model with Alzheimer’s disease 5xFAD at the age of 13 months was used. This line expresses two human genes: APP (amyloid peptide precursor protein gene) with three types of mutations (Swedish (K670N, M671L), Florida (I716V) and London (V717I)), as well as the presenilin 1 gene with mutations (M146L and L286V) characteristic of this disease [167]. Clinically healthy wildtype single-litter mice C57Bl6/j (*n* = 8) were used as a control group. Transgenic 5xFAD mice were randomly divided into two groups: (1) animals receiving intraperitoneal injections of hydroxamic acid **35a** at a dose of 15 mg/kg (10% DMSO + NaCl) for three weeks—the 5xFAD + **35a** group (*n* = 8) and (2) mice receiving an equivalent volume of solvent—the group 5xFAD (*n* = 8). All animals were kept in standard vivarium conditions at a temperature of 22 ± 1 °C and a humidity of 55% indoors with a 12-h light–dark cycle. The animals had no restrictions in the consumption of water and food. The study of the neurobehavioral profile was started on the 15th day of the experiment.

### 4.8. Spatial Learning and Memory Assessment of the Mice

The Morris Water Maze test was performed for five consecutive days to assess the formation of spatial learning and memory dependent on the hippocampus.

The installation consisted of a pool (Open Science, Moscow, Russia) with a diameter of 150 cm, filled to a depth of 40 cm with water (22 ± 1 °C). The lighting conditions were as follows: the dark side of the pool—50 lux; the light side—75 lux. On the sides of the pool there were visual cues—4 figures with different images fixed on racks.

Testing was carried out according to a standard protocol, where for the first four days mice were placed in a pool with a platform hidden under water (1.5 cm below the water surface) in one of the quadrants of the pool. The animals were placed in the pool four times a day from different positions (N, E, S or W) and given 60 s to search for a hidden platform. Mice that did not detect a hidden platform within the allotted time period were carefully directed to it. Upon reaching the platform, all animals were left on it and given 30 s to memorize and study the environment. At the end of the time spent on the platform, the mice were taken from the installation using a tool for extraction in the form of a net. During all the training tests, the location of the platform remained constant. On the fifth day of the experiment, a Probe Trial was conducted without a platform. To do this, the animals were placed in the pool for 90 s and a number of indicators of the neurobehavioral profile were recorded.

All experimental attempts in the Morris water maze were recorded on a camera capturing video images from above the pool, which were processed using EthoVision software.

### 4.9. Extraction of Brain Samples

At the end of the in vivo tests, the procedure of euthanasia animals by cervical dislocation was carried out, after which brain samples were taken. All samples were divided into two parts—(1) to determine the level of malondialdehyde and the functioning of the mitochondrial respiratory chain; (2) to determine the moderation of β-amyloid deposits.

### 4.10. Assay of MDA Level in Brain Samples

To determine the level of malondialdehyde in the brain of animals, the samples were homogenized in a buffer solution and subjected to a centrifugation procedure at 12,000 rpm for 10 min at 4 °C, after which the infusion fluid was collected for subsequent analysis. Quantitative determination of the protein was carried out as described earlier [168].

The content of malondialdehyde was determined using a modified TBA test [126]. According to the experimental scheme, the resulting homogenate was introduced into the corresponding wells of a 96-well tablet, after which the TBARS reagent was immediately and carefully added to the samples and incubated for 90 min at 90 °C. After the incubation time, the samples were centrifuged at 6000 rpm for 15 min. The optical density of the selected suprasetting fluid was measured using a multifunctional Cytation™3 flatbed analyzer (BioTek Instruments, Inc., Winooski, VT, USA) at λ = 540 nm.

### 4.11. Measurement of Mitochondrial Oxygen Consumption Rate

The analysis of the work of the complexes of the electron transport chain of mitochondria was carried out on the mitochondrial p2 fraction obtained from brain samples of animals of experimental groups. Mitochondrial respiration was determined by the rate of oxygen consumption by organelles using the Seahorse XFe96 cell metabolism analyzer (Seahorse Bioscience, North Billerica, MA, USA), in full compliance with the protocol described in [169].

### 4.12. Histology of β-Amyloid Deposits in Brain Samples

After cervical dislocation, the half of brain was dissected and fixed with Carnoy’s solution (ethanol–chloroform–glacial acetic acid, 6:3:1) for 17 h. Tissue was dehydrated through ethanol solutions (96% (I), (II) and (III) for 10 min; 100% (I) and (II) for 10 min). The sample was incubated consecutively with ethanol–chloroform (1:1) for 30 min, chloroform (I) for 1 h and chloroform (II) overnight, and embedded in paraffin (3 times, for 1 h each) at 62 °C. Paraffin sections (8 μm thick) were mounted on poly-L-lysine-coated slides through the scheme, as described previously [170] in a covered 400 um hippocampal zone. Slides were deparaffinized in a xylene for 20 min; rehydrated through ethanol solutions (100% for 20 min, 95% for 5 min, and 50% for 10 min); washed in deionized water for 15 min; stained with 0.5% Congo Red in 50% ethanol for 5 min; differentiated by 0.2% KOH in 80% ethanol for 2 min; washed again and embedded with water-based Epredia™ Immu-Mount™ medium (Thermo Fisher Scientific Inc., Paisley, UK) [171]. The stained deposits were evaluated using a ZEISS LSM 880 laser scanning microscope (Carl Zeiss, Oberkochen, Germany) in the tile scan mode. A total of 10 images per animal was reviewed. Image processing of β-amyloid deposits’ morphometric analysis was based on QuPath’s pixel classifier, a machine learning algorithm for the detection of different areas (such as hippocampal, thalamus and cortex zones) with control of the histology atlas within images.

### 4.13. Statistics

The data of all experiments are presented as an average ± SEM. The differences between the groups were studied using one-way ANOVA by Bonferroni’s and Dunnett’s post hoc tests. The statistical analysis was performed using GraphPad Prism 5 (GraphPad Software, San Diego, CA, USA).

No animals were excluded from the results of in vivo experiments.

## 5. Conclusions

Alzheimer’s disease is a serious socio-economic problem of a large number of developed countries. Despite the fact that a wide range of protocols for the treatment of this disease has been demonstrated in recent years, the currently available approaches still do not have a high efficiency. This demonstrates the need to develop new strategies for future clinical applications that could eliminate critical gaps in existing applications.

In this study, the analysis of neuroprotective properties of novel monoterpene-based hydroxamic acids synthesized as promising agents against Alzheimer’s disease was carried out using an integrated approach, including a series of in vitro, in vivo and ex vivo experiments, as well as a docking procedure. The compound-leader, (*S*,*E*)-*N*-hydroxy-4-(3-oxo-3-(((4-(prop-1-en-2-yl)cyclohex-1-en-1-yl)methyl)amino)prop-1-en-1-yl)benzamide **35a** was discovered. The compound demonstrated the most promising profile of biological activity. This was expressed in the manifestation of excellent HDAC6-inhibiting properties and antiradical activity by this hydroxamic acid, as well as the ability to modulate the aggregation of the pathological form of the β-amyloid peptide 1-42, which was reflected in the study of processes associated with the formation of hippocampus-dependent spatial memory in transgenic animals. The **35a** restored cognitive dysfunctions in genetically modified 5xFAD mice, whose pathological phenotype includes disorders observed in Alzheimer’s disease. Moreover, the post mortem analysis of animal brain samples from experimental groups demonstrated a significant decrease in the level of malondialdehyde, stimulation of the mitochondrial respiratory chain complexes up to the level of clinically healthy wild type mice and slowed down the rate of β-amyloid formation in the hippocampal, thalamus and cortex zones, which confirmed in vitro neuroprotective effects of hydroxamic acid.

All of this may indicate that hydroxamic acid with an aromatic linker and a Cap group containing the (-)-perillil fragment **35a** may be offered for use as a promising anti-Alzheimer’s drug.

## Figures and Tables

**Table 1:** Values of IC_50_ (µM) inhibition of HDAC6 by novel monoterpene-derived hydroxamic acids.

Compound	HDAC6-Inhibitory Effect	Compound	HDAC6-Inhibitory Effect
**5**	>100	**30**	0.56 ± 0.01
(-)-**19**	>100	**31**	0.74 ± 0.02
**10**	>100	**35a**	3.85 ± 0.24
**16**	87.38 ± 2.33	**35c**	6.90 ± 0.43
(+)-**19**	>100	**35b**	8.23 ± 0.40
**8**	>100	**38b**	4.78 ± 0.04
(+)-**13**	>100	**38a**	7.74 ± 0.11
(-)-**13**	>100		

**Table 2:** Molecular docking of hydroxamic acid against HDAC6 (PDB ID: 5EDU [48]).

Compound	H-Bonds	Other Interactions
**Trichostatin A**	HIS610	Pi-Pi: HIE500
**31**	SER568 HIS610 TYR782	---
**30**	SER568 GLY619 TYR782	---
**35a**	HIE500 SER568 TYR782	Pi-Pi: PHE620 HIE651
**38b**	SER568	Pi-Pi: PHE680
**38a**	GLY619	Pi-Pi: PHE680
**35b**	HIE500 SER568 TYR782	Pi-Pi: HIE500 PHE680
**35c**	SER568 GLY619	Pi-Pi: PHE620 HIE651 PHE680
**38c**	TYR782	---

**Table 3:** Values of IC_50_ (µM) cytotoxic action of novel monoterpene-derived hydroxamic acids.

Compound	IC_50_	Compound	IC_50_
**5**	>100	**30**	>100
(-)-**19**	>100	**31**	89.36 ± 3.92
**10**	>100	**35a**	89.23 ± 2.09
**16**	>100	**35c**	86.67 ± 1.45
(+)-**19**	>100	**38c**	90.45 ± 2.90
**8**	>100	**35b**	>100
(+)-**13**	>100	**38b**	>100
(-)-**13**	>100	**38a**	>100

**Table 4:** Values of ADME/Tox parameters for the obtained compounds.

ID	*	logPo/w	logS	PCaco	logBB	PMDCK	#M	%HOA	RO5	RO3
**30**	0	1.454	−2.742	107.287	−1.829	134.863	7	71.801	0	1
**35a**	0	2.665	−4.918	209.505	−1.809	91.336	5	84.092	0	0
**31**	0	1.773	−3.192	72.881	−2.137	96.298	7	70.664	0	1
**38c**	1	2.11	−4.166	120.574	−1.793	50.268	0	76.551	0	0
**35c**	1	2.477	−4.663	218.955	−1.642	95.797	0	83.336	0	0
**35b**	0	2.633	−4.905	210.157	−1.836	91.643	2	83.934	0	0
**38b**	0	2.61	−4.872	198.621	−1.853	86.218	2	83.356	0	0
**38a**	0	2.642	−4.934	198.46	−1.846	86.143	5	83.542	0	0

*—The number of values of properties or descriptors that go beyond 95% of the range of similar values for known drugs; logPo/w—the predicted value of the octanol/water partial coefficient; logS—the predicted value of solubility in water in mol/l; PCaco—assessment of permeability through cells of the Caco-2 line in nm/s; logBB—assessment of permeability through the blood–brain barrier; PMDCK—assessment of permeability through MDCK cells in nm/s (used as a model of penetration through the BBB); #M—the number of possible metabolic reactions into which the substance can enter; %HOA—predicted oral absorption; RO5—number of violations of the Lipinski rule of five; RO3—number of violations of the Jorgensen rule of three.

## Data Availability

Samples of the compounds and data used during the current study are available from the corresponding author.

## Supplementary Materials

The following supporting information can be downloaded at: https://www.mdpi.com/article/10.3390/ijms24119743/s1.
