# Supplementary material for: Elaboration of the Effective Multi-Target Therapeutic Platform for the Treatment of Alzheimer’s Disease Based on Novel Monoterpene-Derived Hydroxamic Acids
Source: Int J Mol Sci. 2023 Jun 4;24(11):9743. doi: 10.3390/ijms24119743 (PMC10254045; doi:10.3390/ijms24119743)
Supplement: Supplementary file 1 [file ijms-24-09743-s001.zip › ijms-2394340-supplementary.pdf]

Elaboration of the Effective Multi-Target Therapeutic Platform for the Treatment of Alzheimer's Disease Based on Novel Monoterpene-Derived Hydroxamic Acids

Yulia Aleksandrova[1], Aldar Munkuev[2], Evgenii Mozhaitsev[2], Evgenii Suslov[2], Dmitry Tsypyshev[2], Kirill Chaprov[1], Roman Begunov[3], Konstantin Volcho[2], Nariman Salakhutdinov[2] and Margarita Neganova[1,*]

[1] Institute of Physiologically Active Compounds at Federal Research Center of Problems of Chemical Physics and Medicinal Chemistry, Russian Academy of Sciences, Severnij Pr. 1, Chernogolovka 142432, Russia
[2] Department of Medicinal Chemistry, N. N. Vorozhtsov Novosibirsk Institute of Organic Chemistry, Siberian Branch, Russian Academy of Sciences, Lavrentiev Ave., 9, Novosibirsk 630090, Russia
[3] P. G. Demidov Yaroslavl State University, Matrosova Ave., 9, Yaroslavl 150003, Russia

* Correspondence: neganovam@ipac.ac.ru; Tel.: +7-(962)-937-68-69

^{1}H NMR spectrum of compound **5**

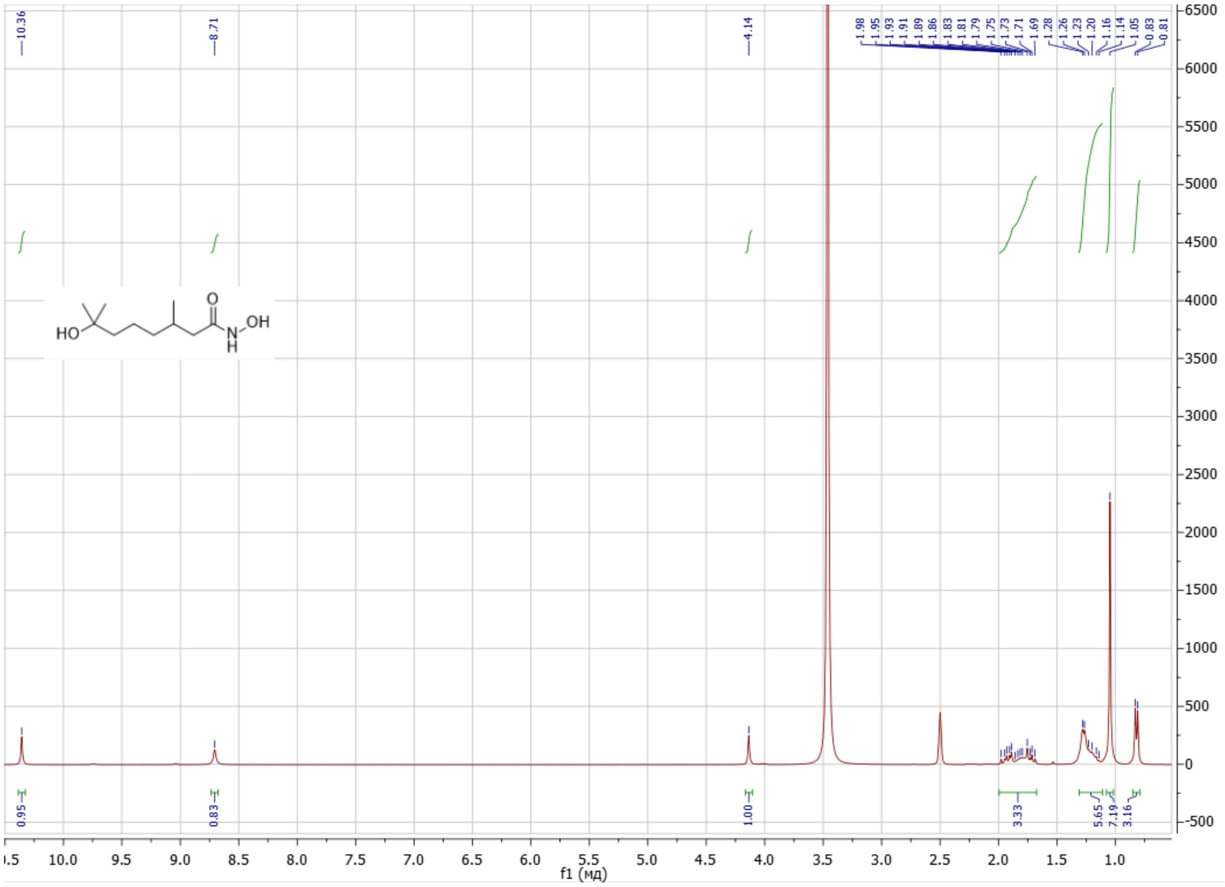

^{13}C NMR spectrum of compound **5**

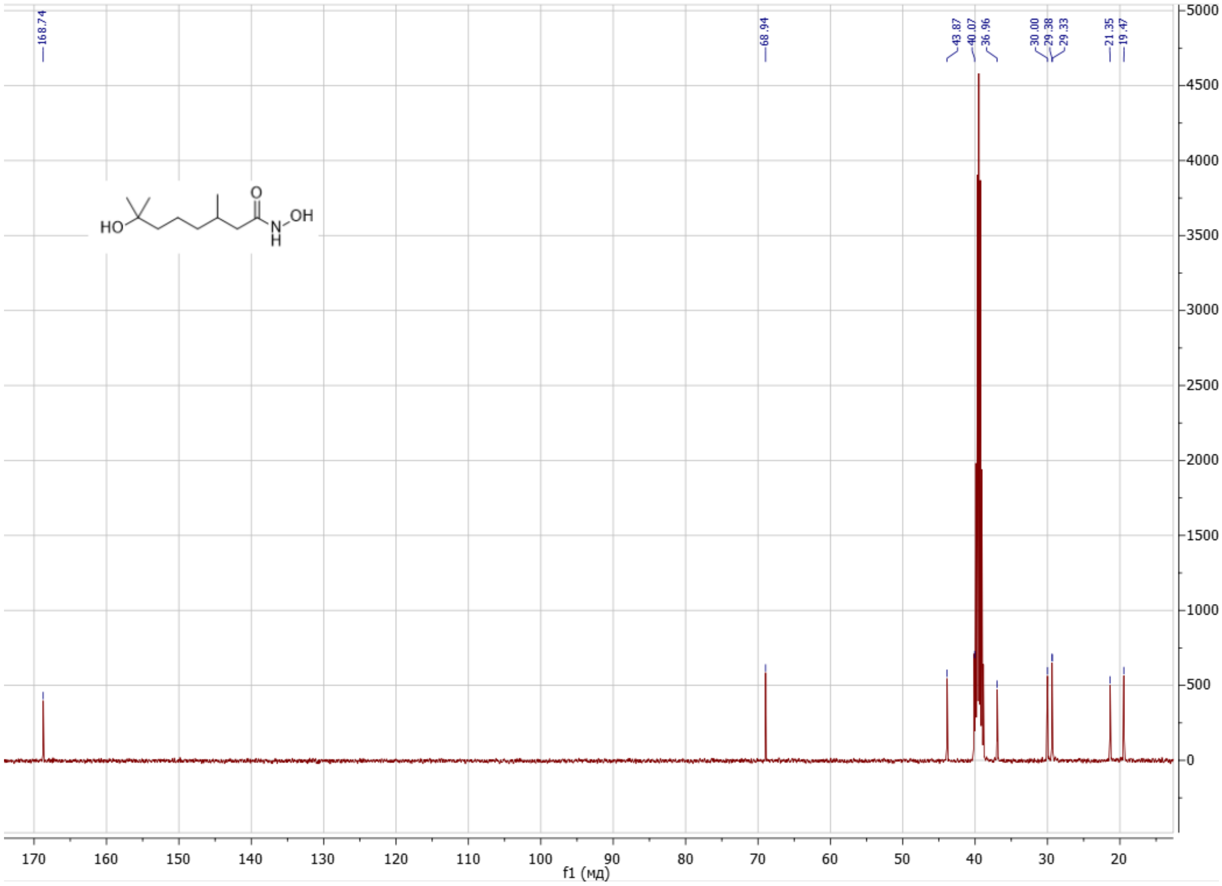

Mass-spectrum of compound **5**

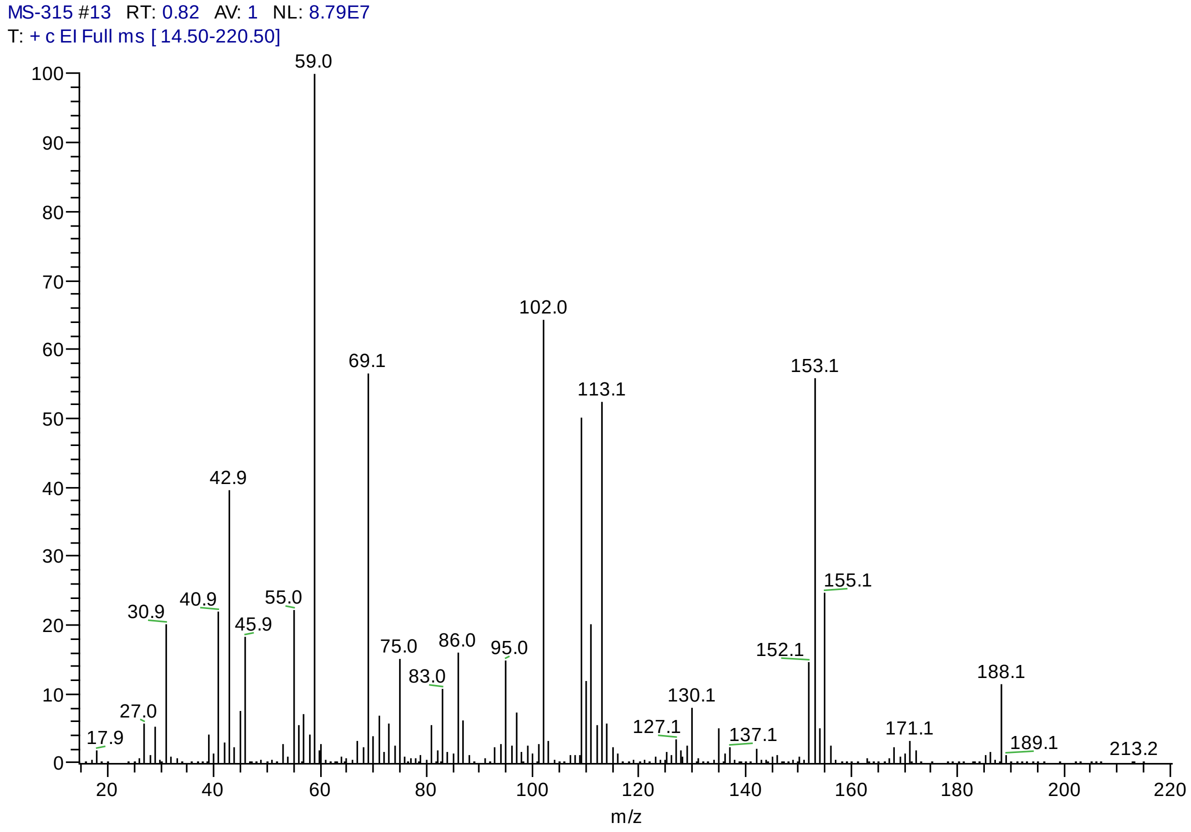

^{1}H NMR spectrum of compound **8**

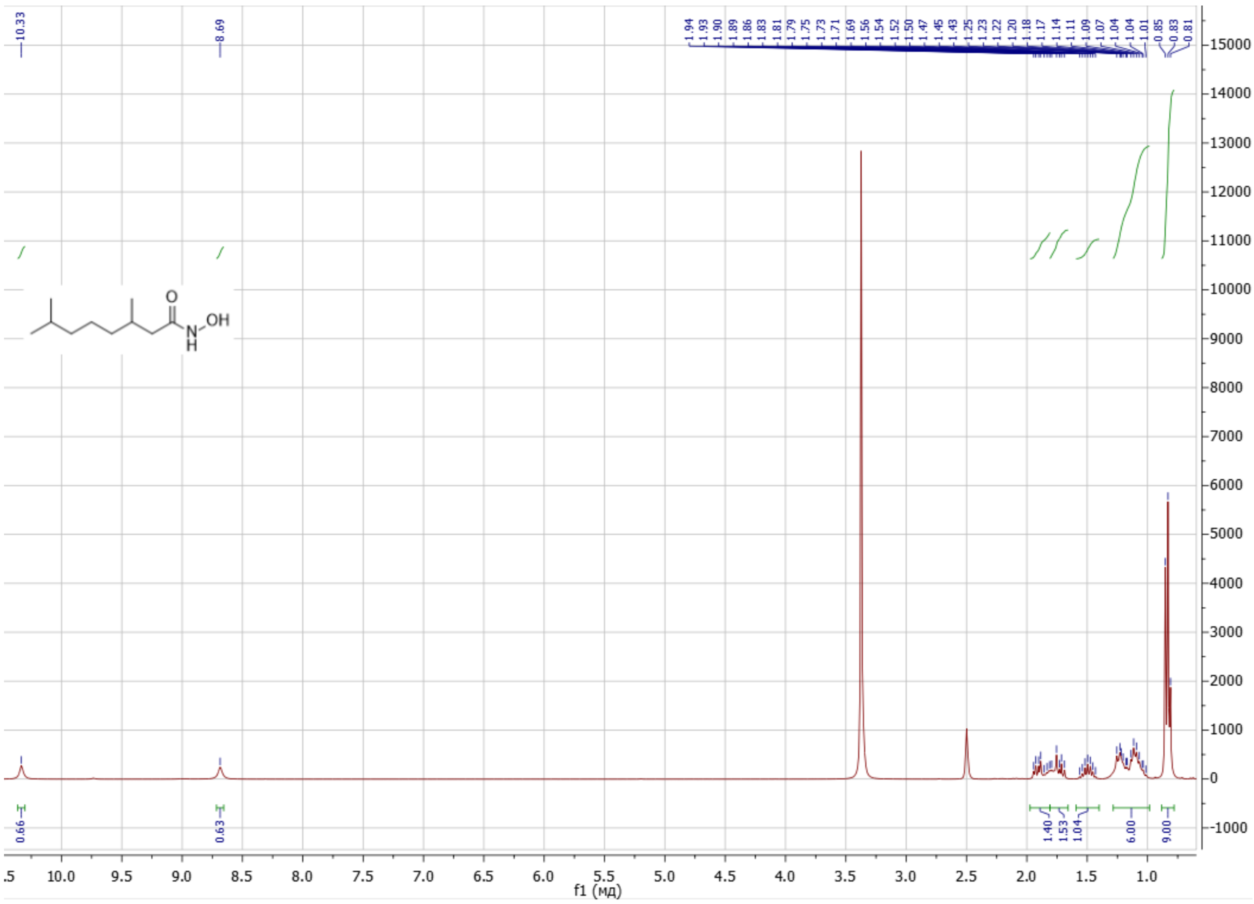

^{13}C NMR spectrum of compound **8**

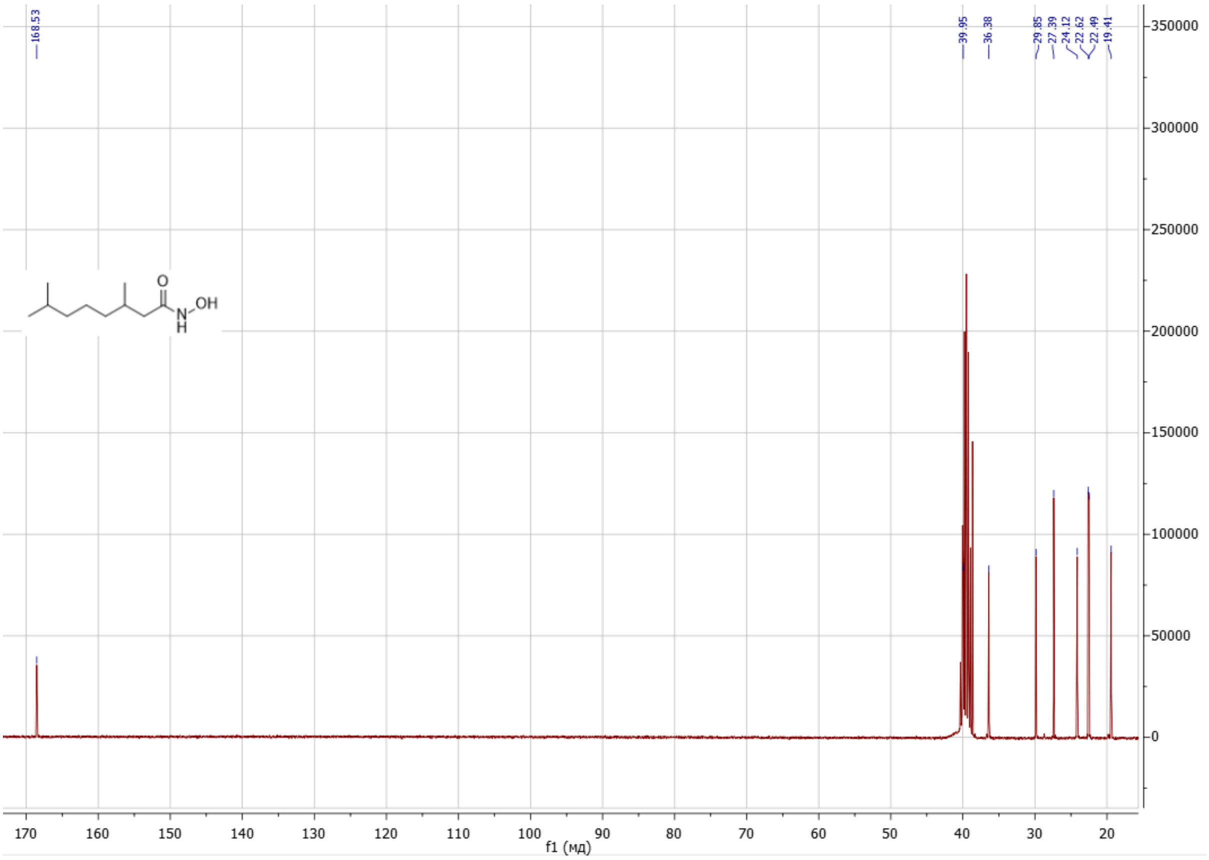

Mass-spectrum of compound **8**

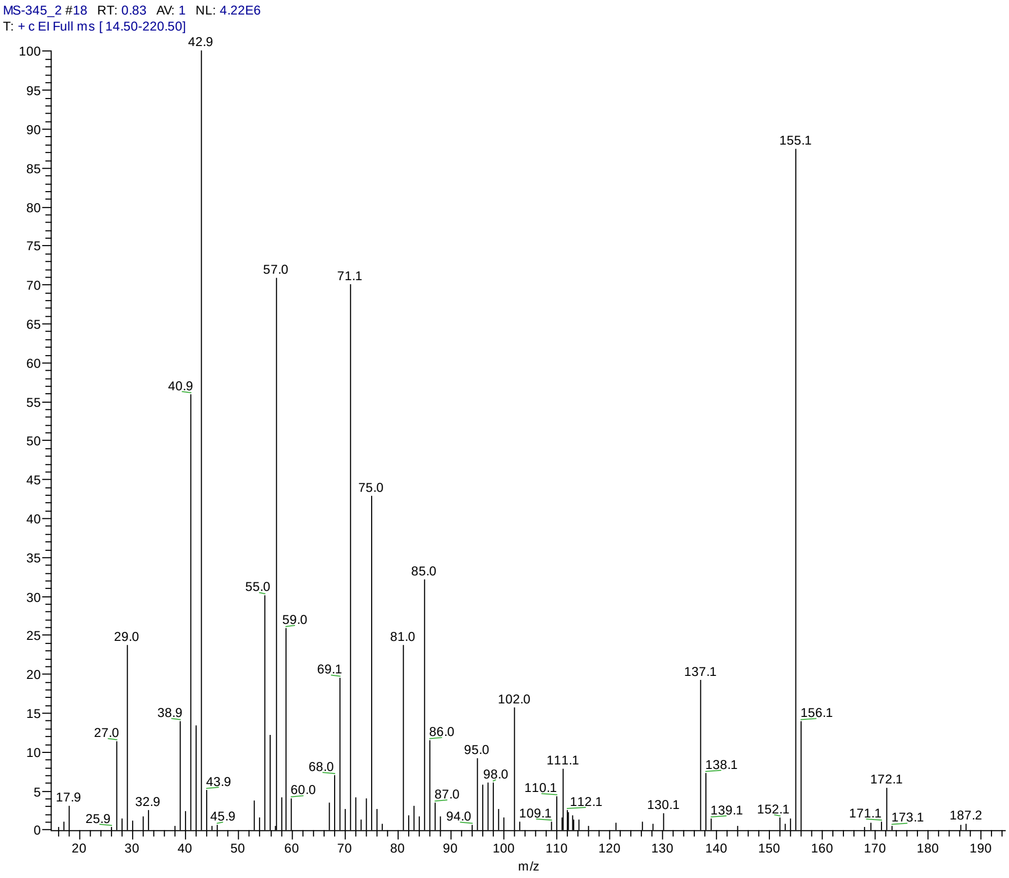

^{1}H NMR spectrum of compound **10**

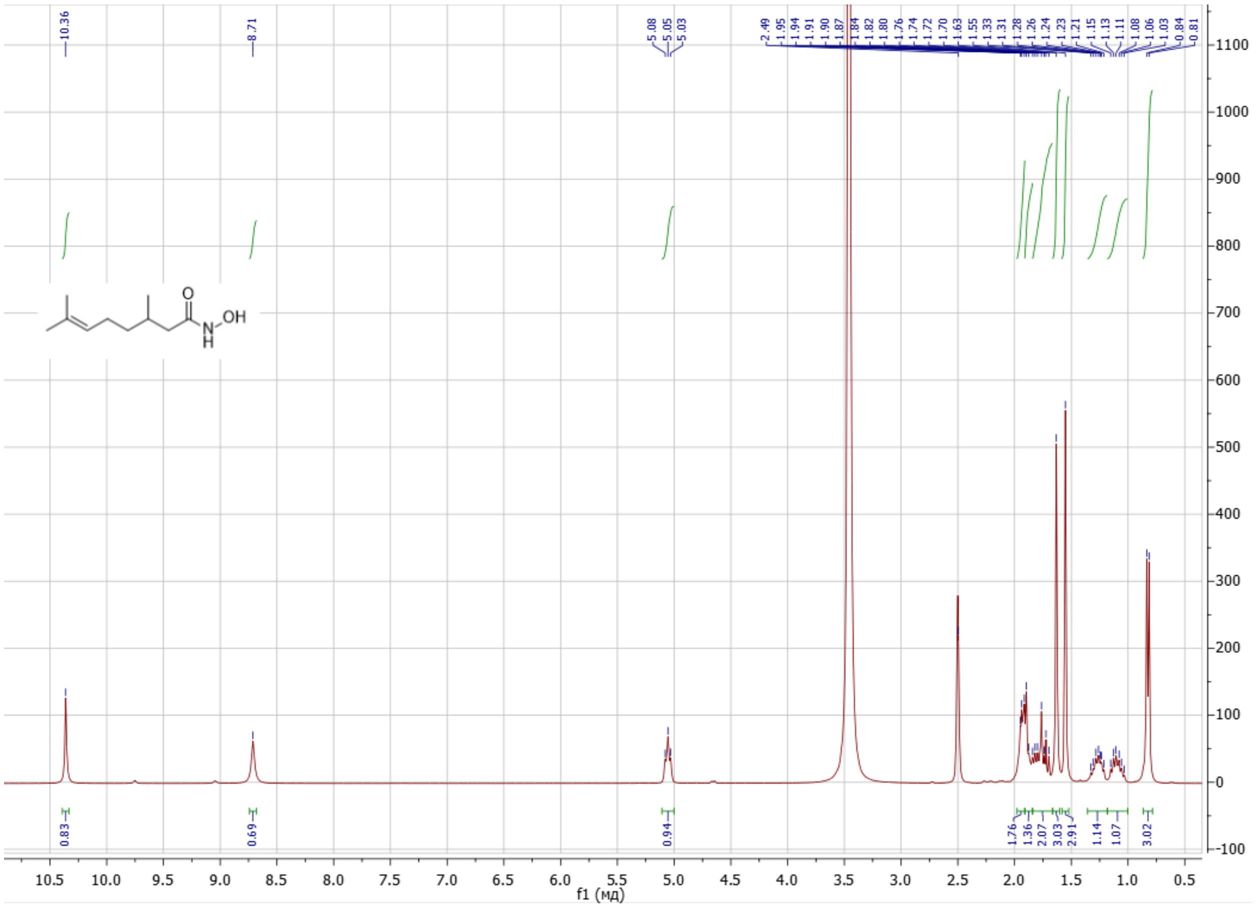

^{13}C NMR spectrum of compound **10**

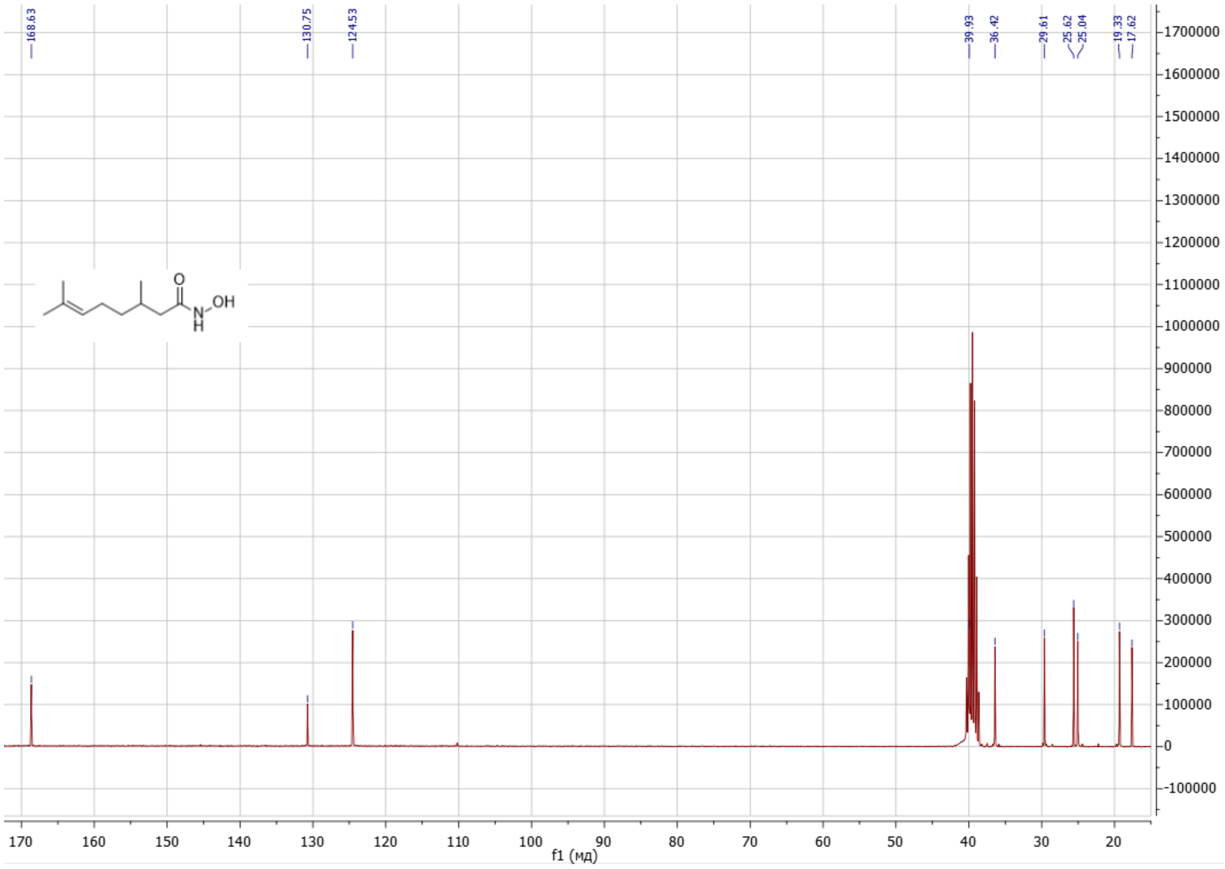

Mass-spectrum of compound **10**

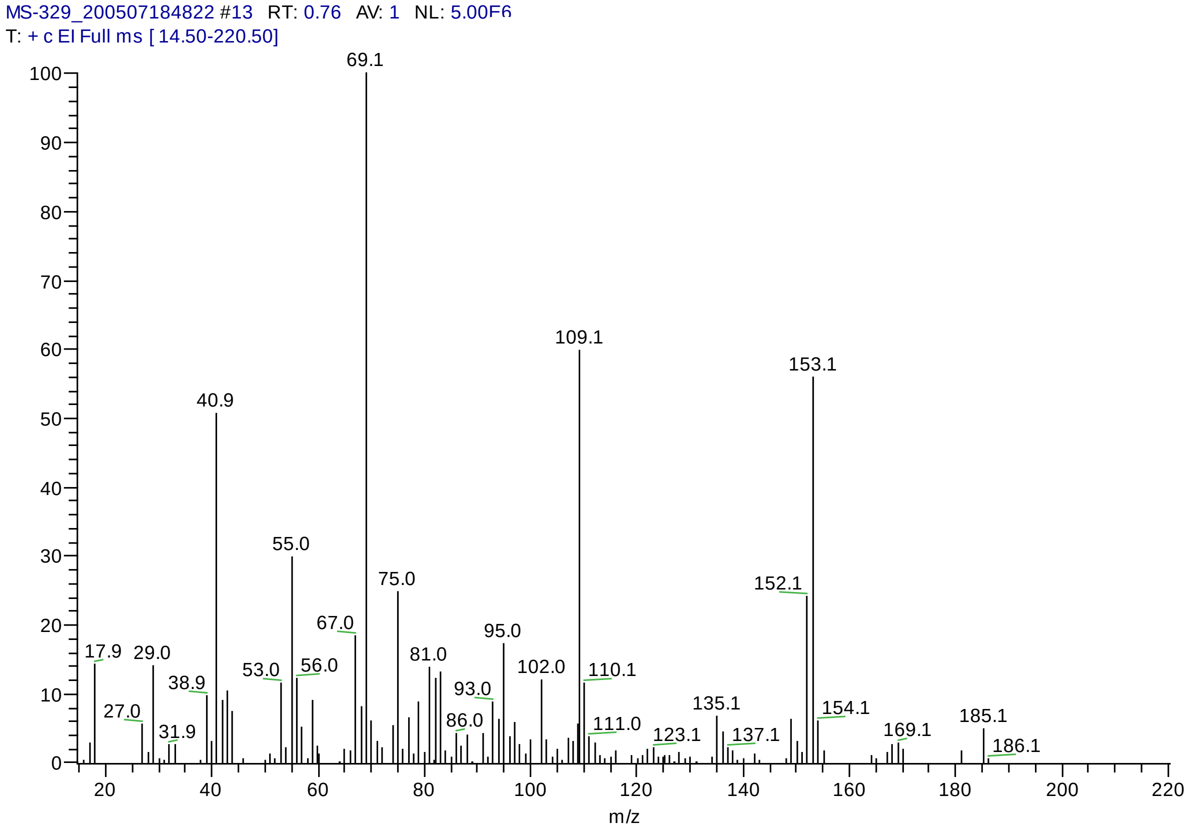

^{1}H NMR spectrum of compound (+)-**13**

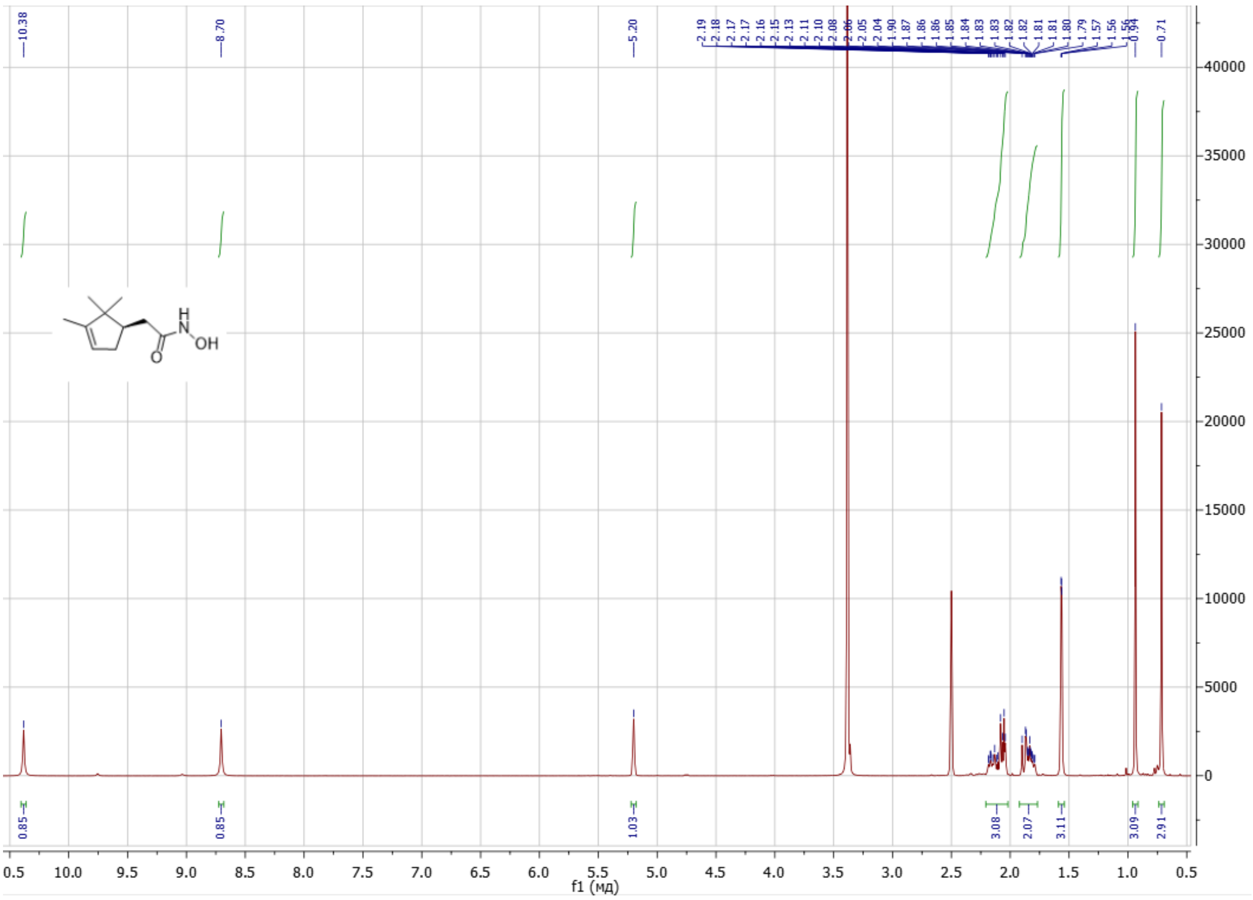

^{13}C NMR spectrum of compound (+)-**13**

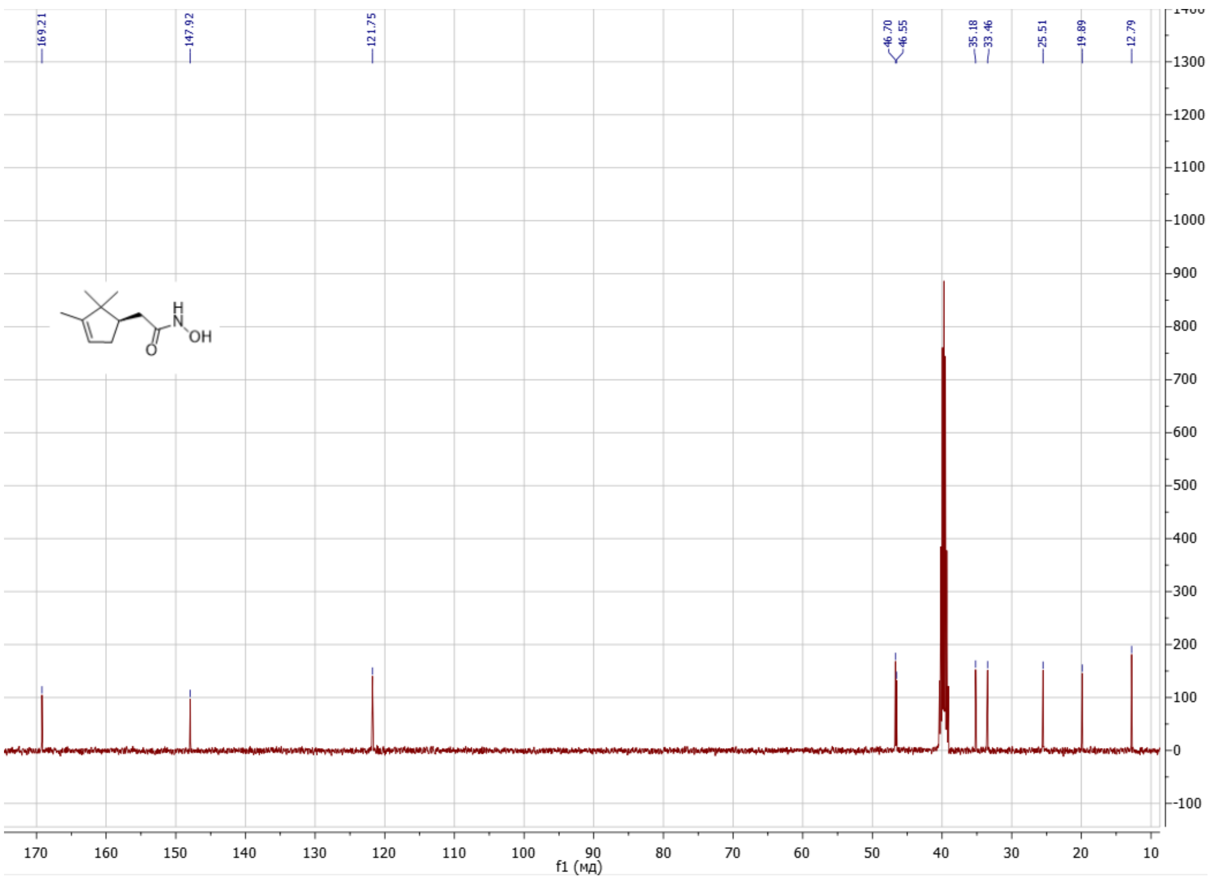

Mass-spectrum of compound (+)-**13**

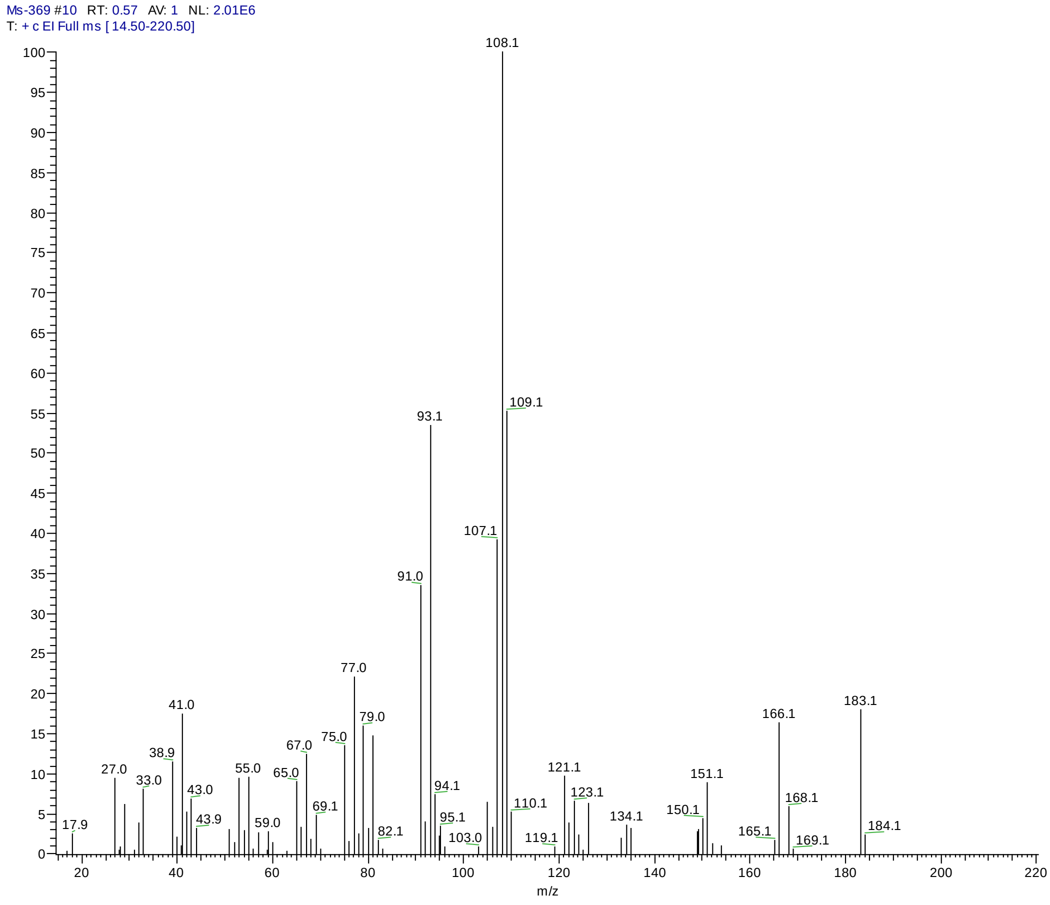

^{1}H NMR spectrum of compound **16**

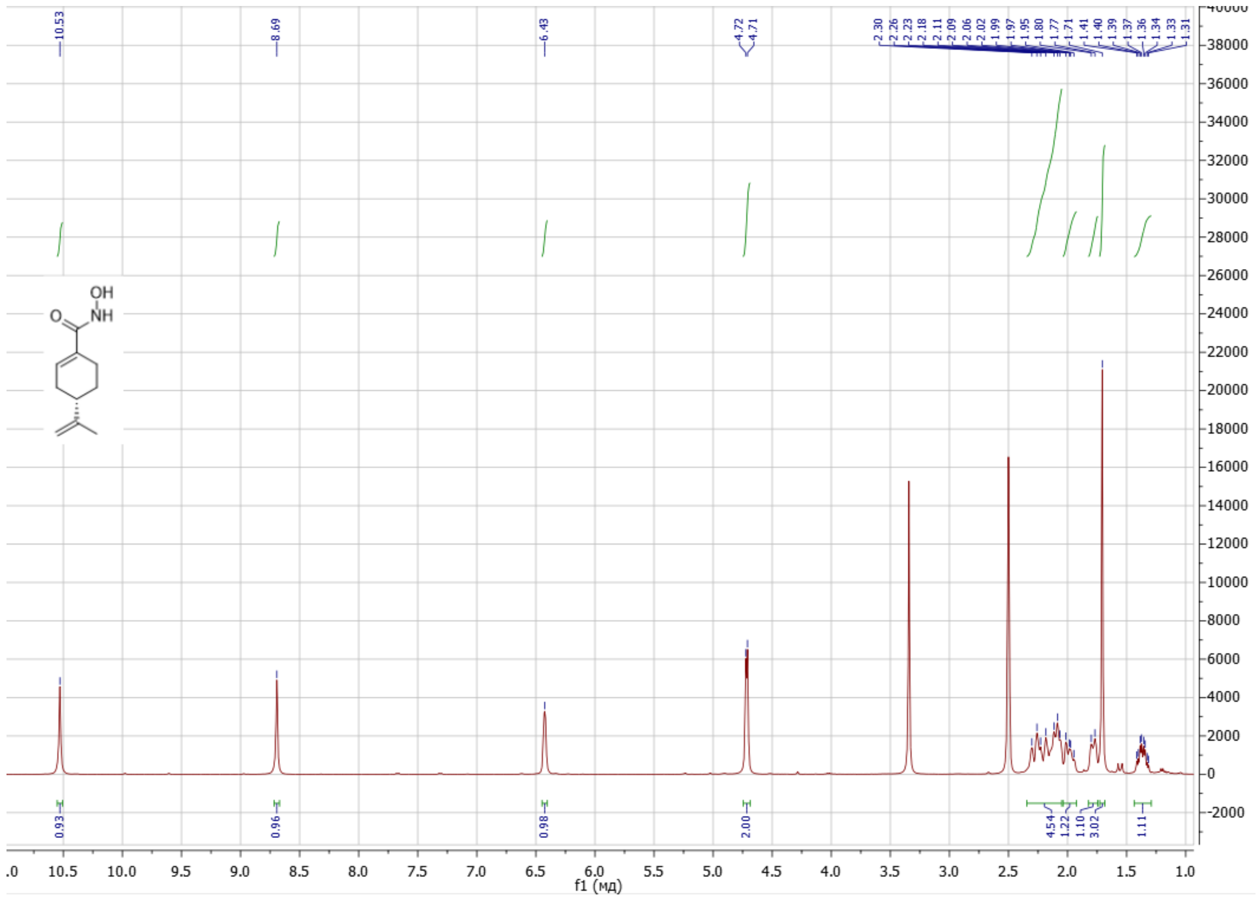

^{13}C NMR spectrum of compound **16**

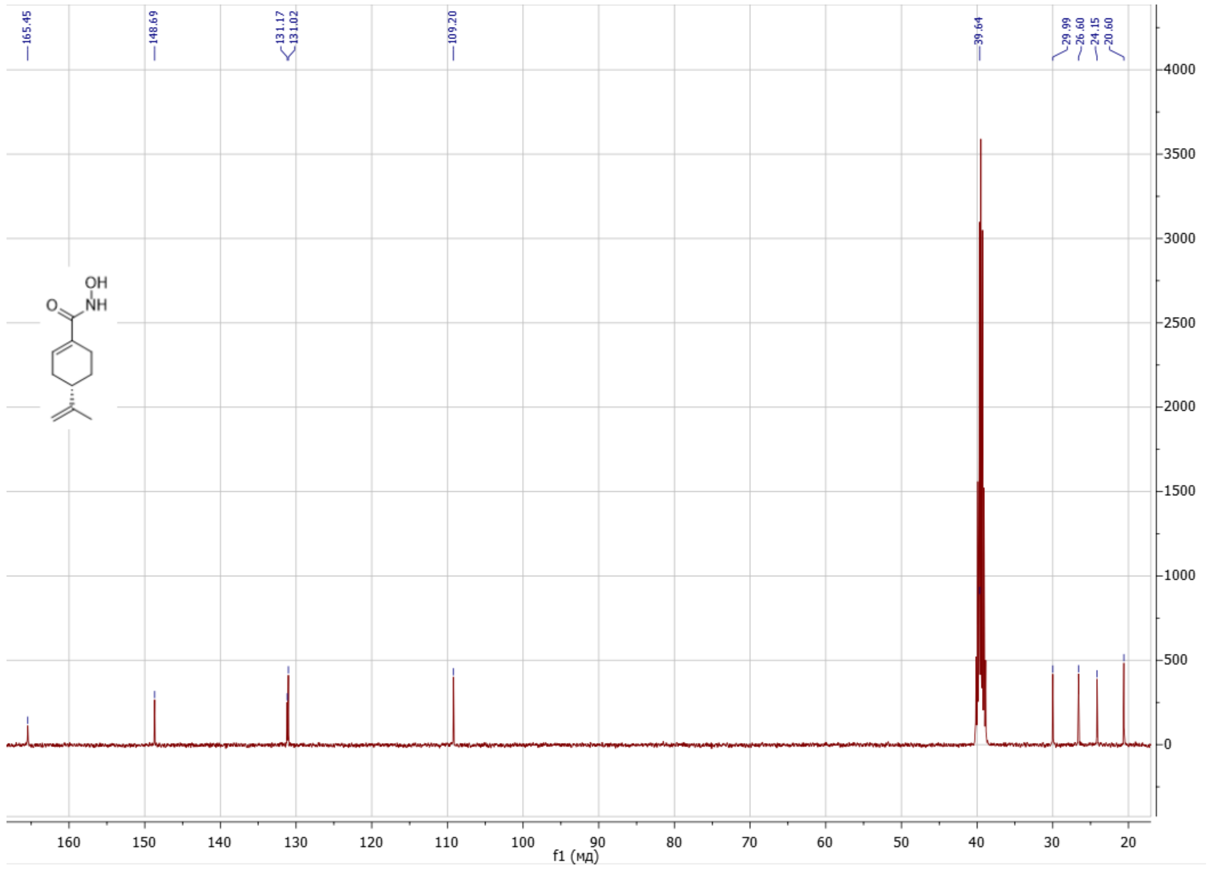

Mass-spectrum of compound **16**

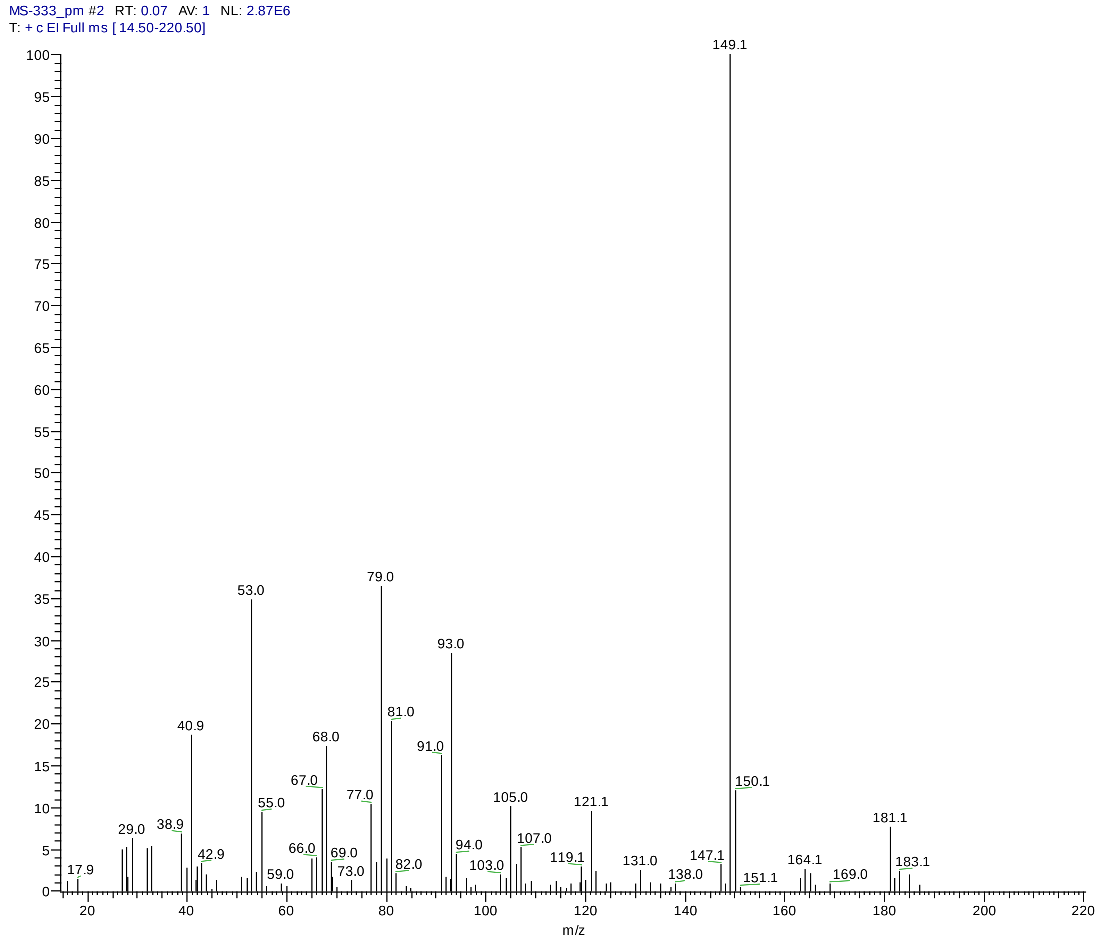

^{1}H NMR spectrum of compound (-)-**19**

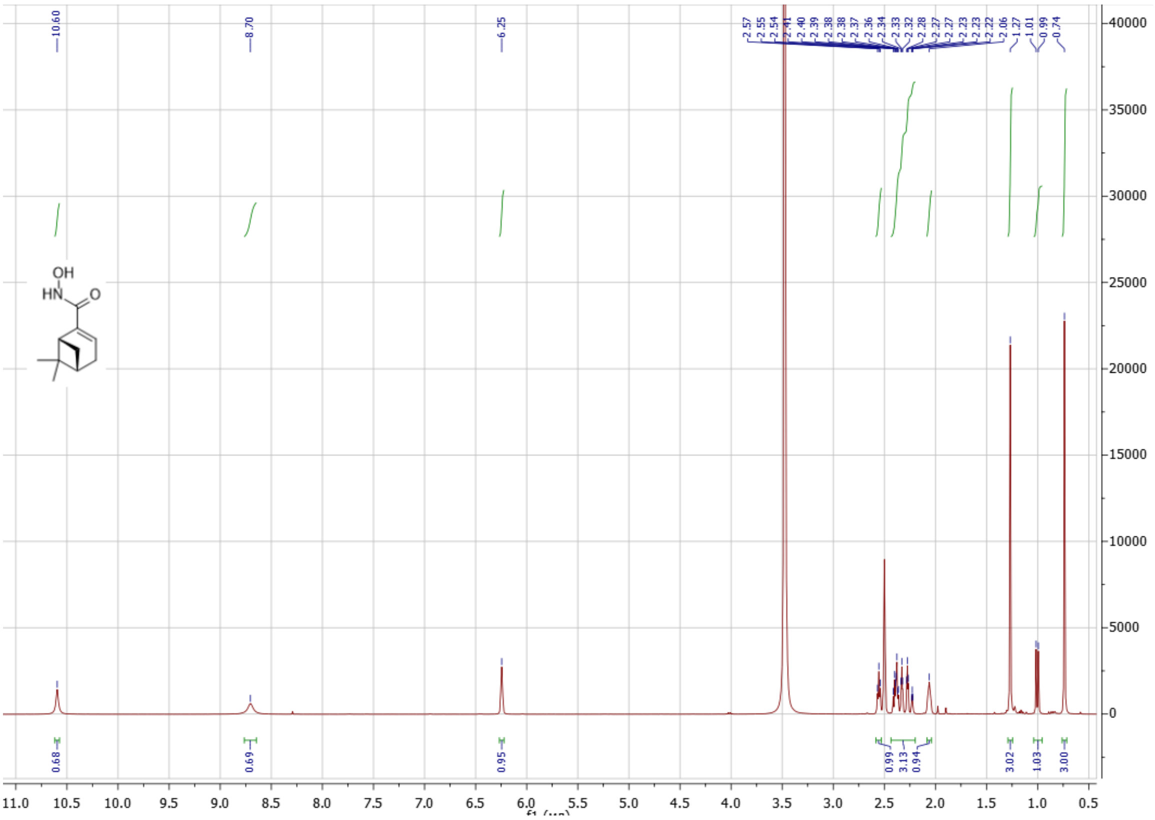

^{13}C NMR spectrum of compound (-)-**19**

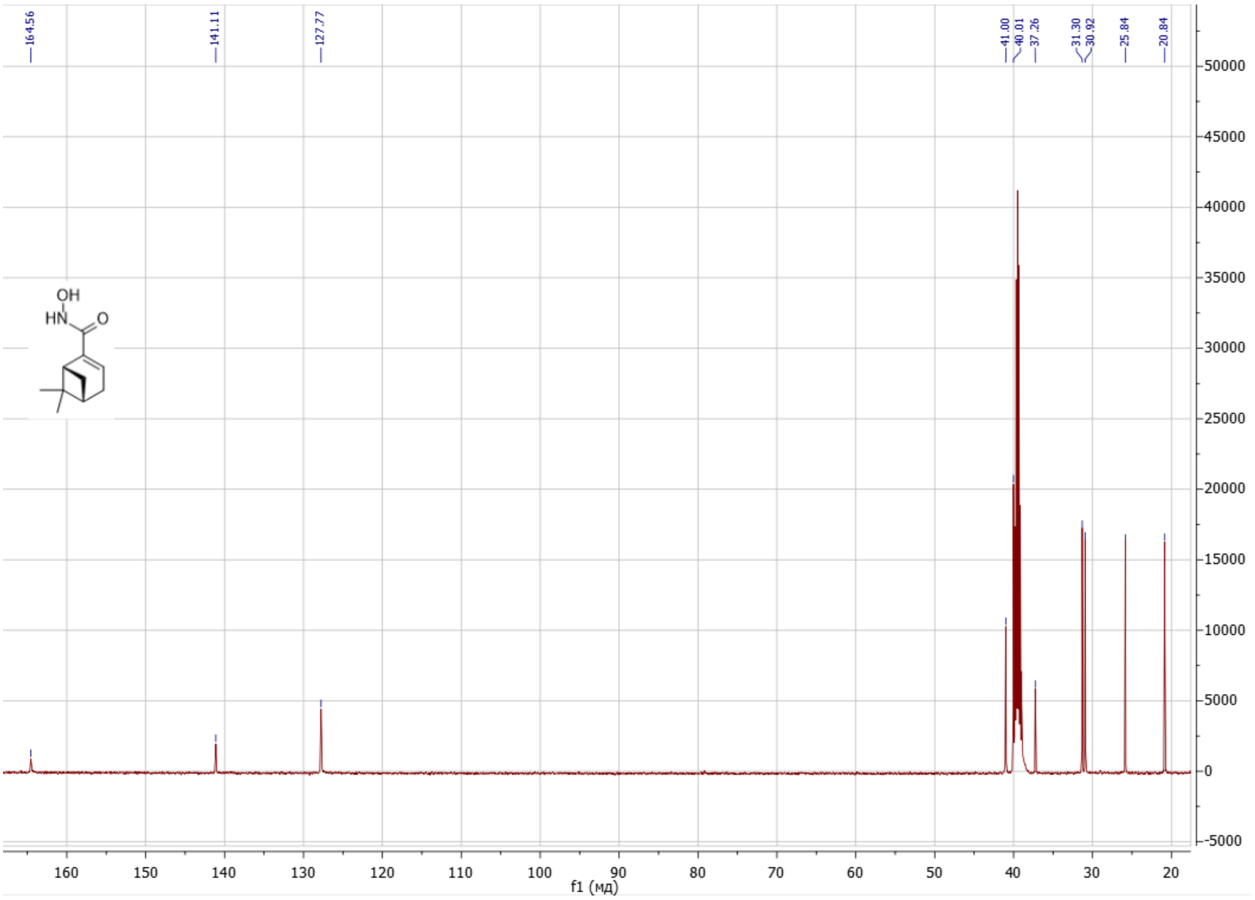

Mass-spectrum of compound (-)-**19**

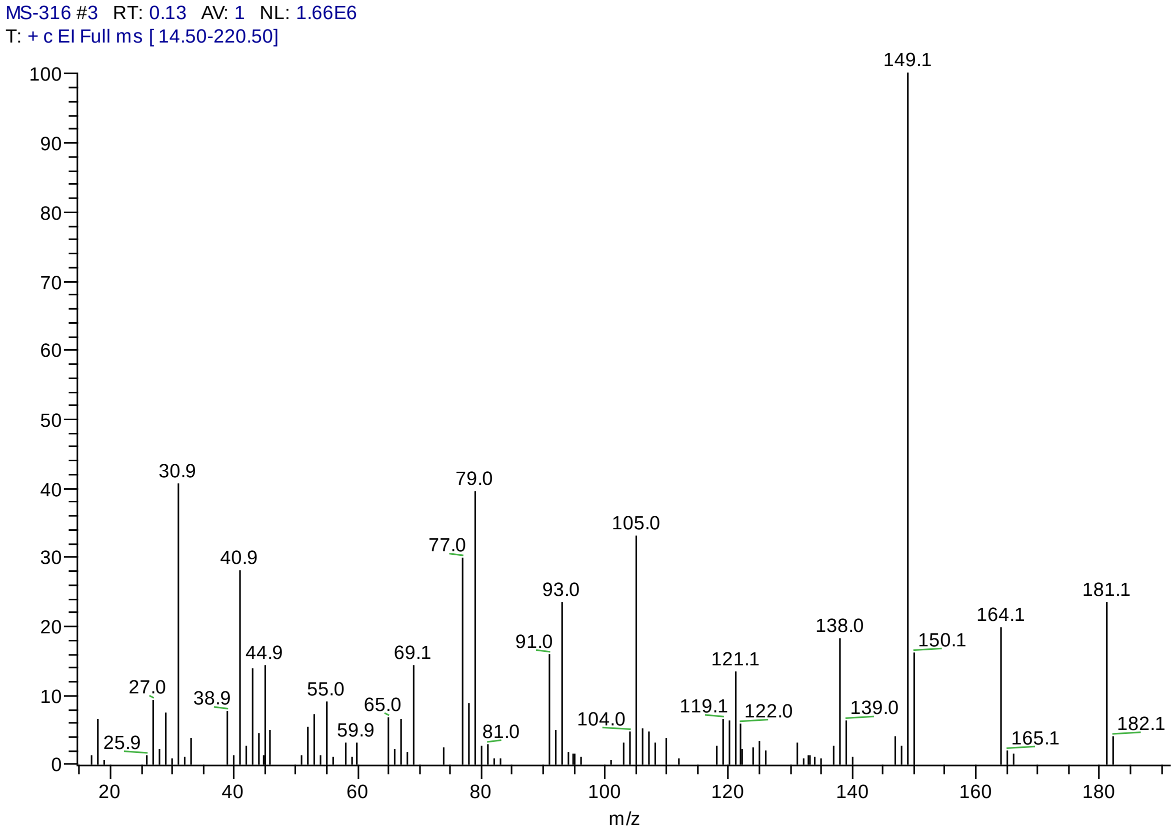

^{1}H NMR spectrum of compound **30**

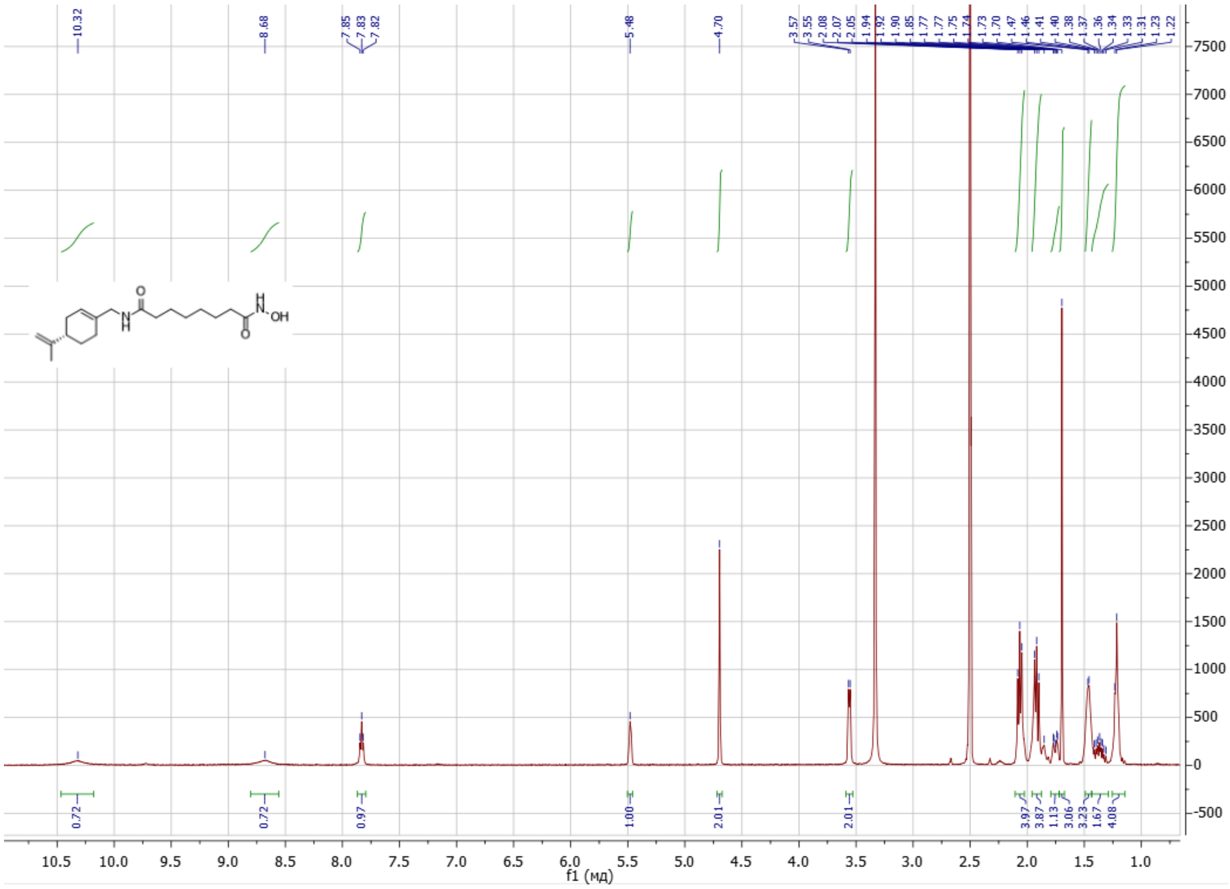

^{13}C NMR spectrum of compound **30**

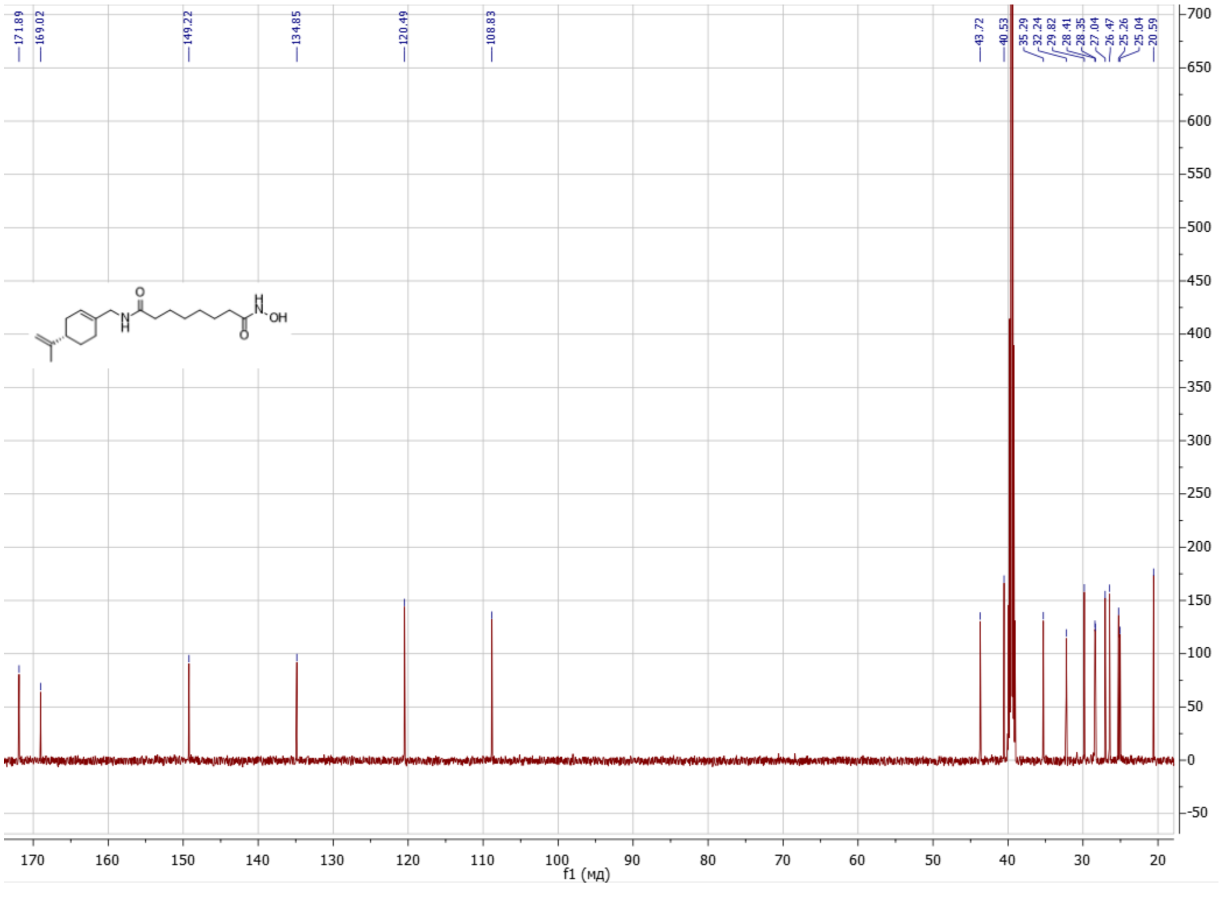

Mass-spectrum of compound **30**

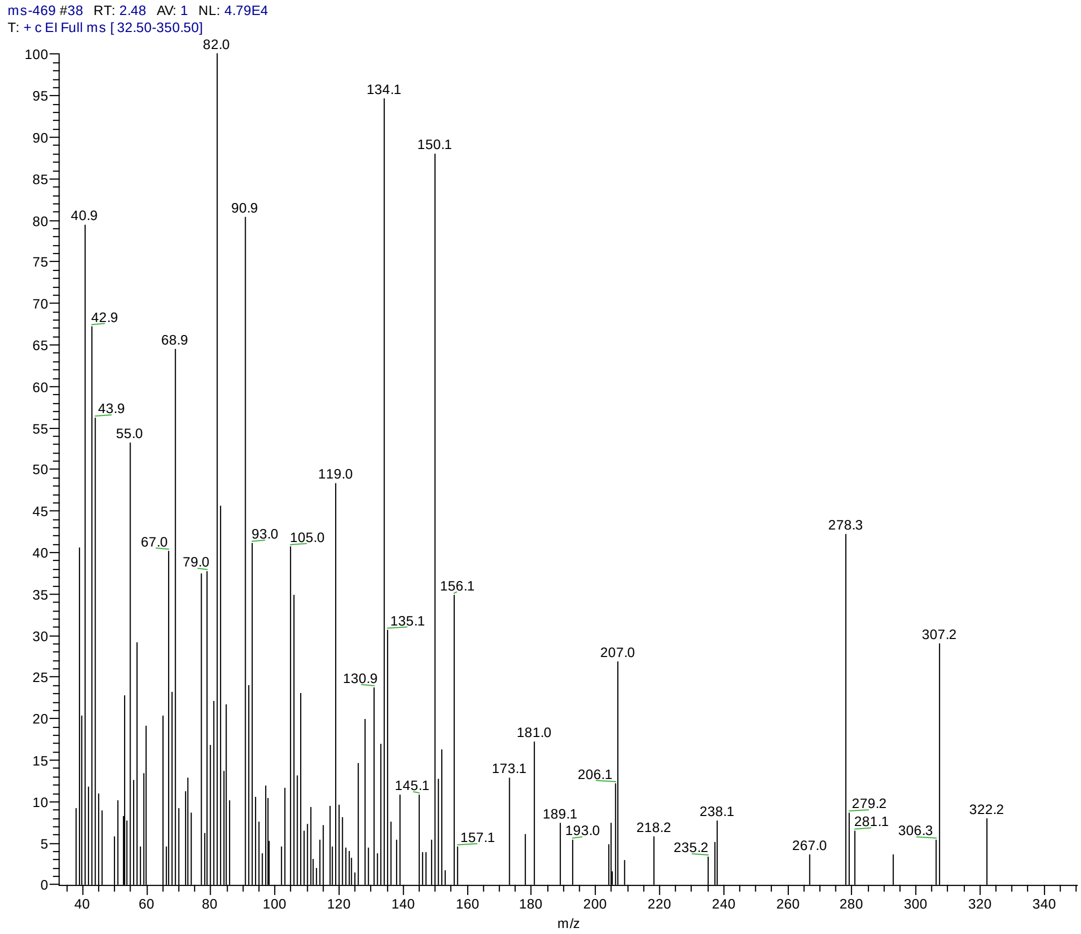

^{1}H NMR spectrum of compound **31**

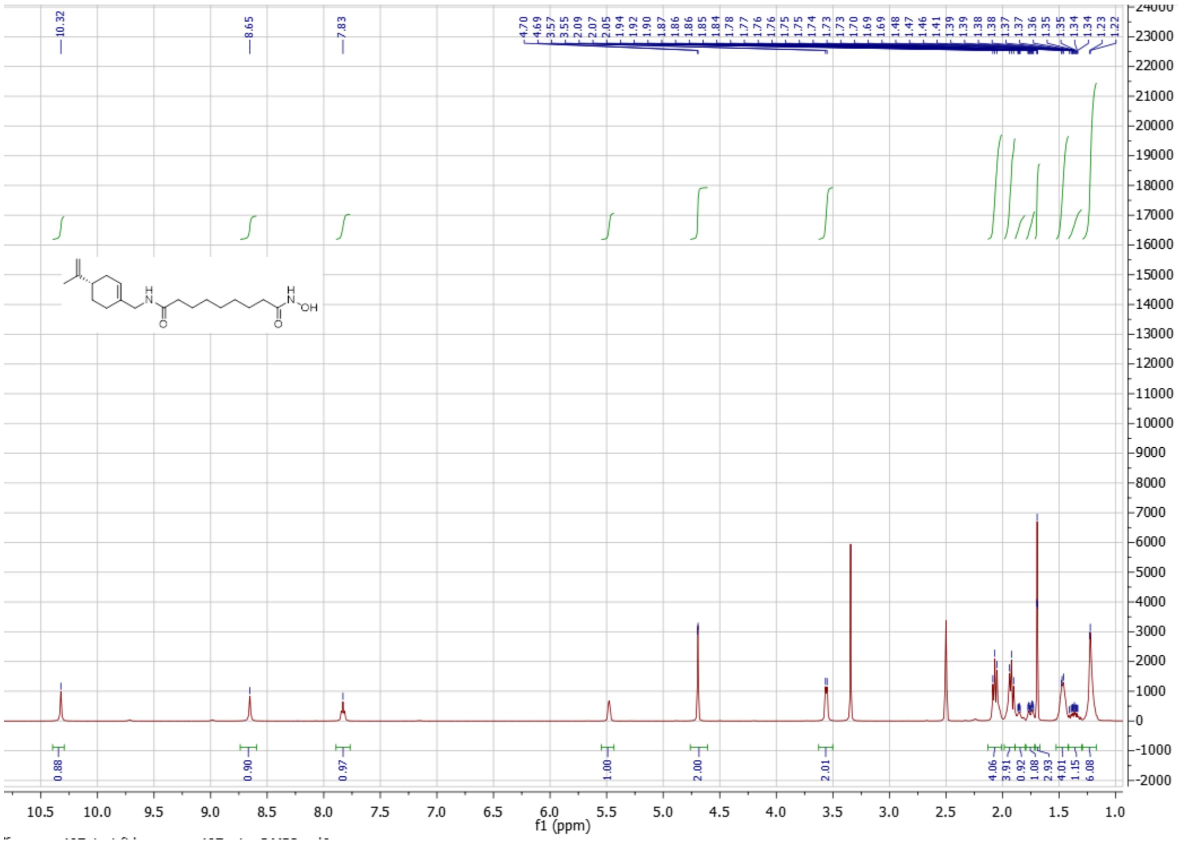

^{13}C NMR spectrum of compound **31**

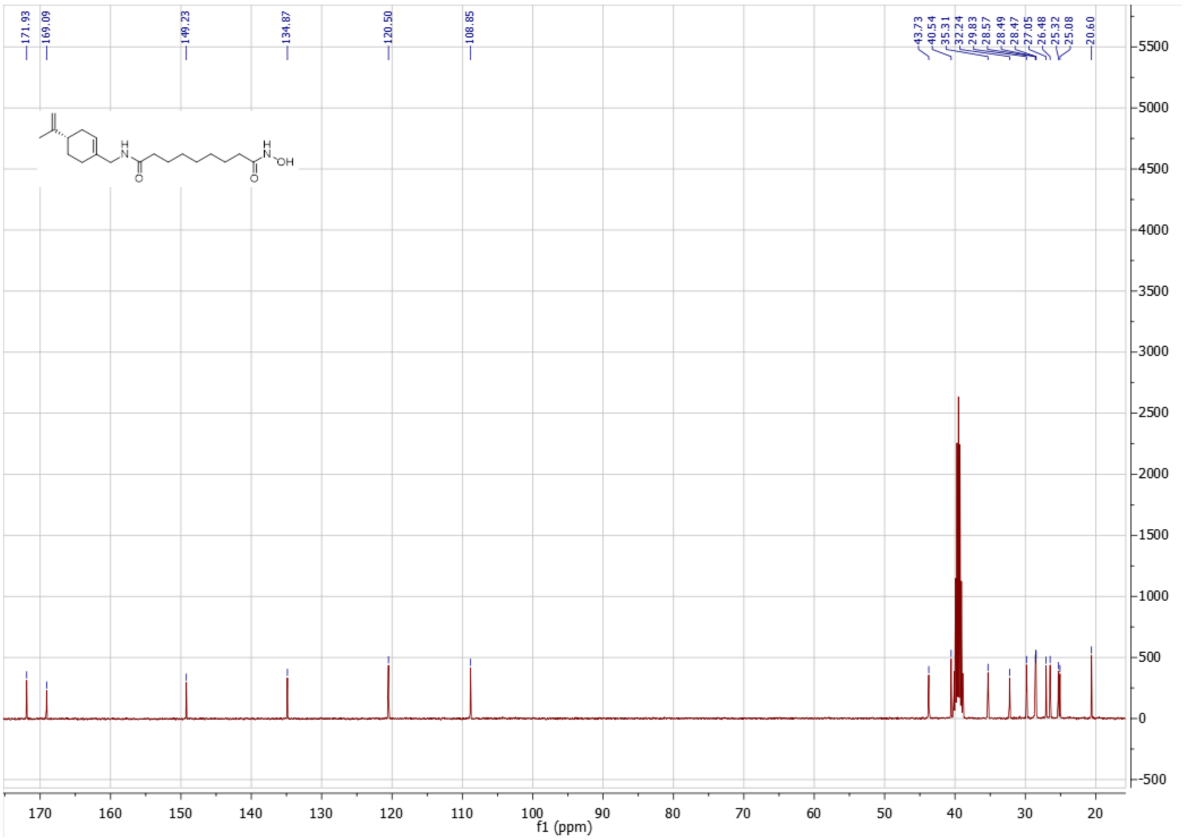

Mass-spectrum of compound **31**

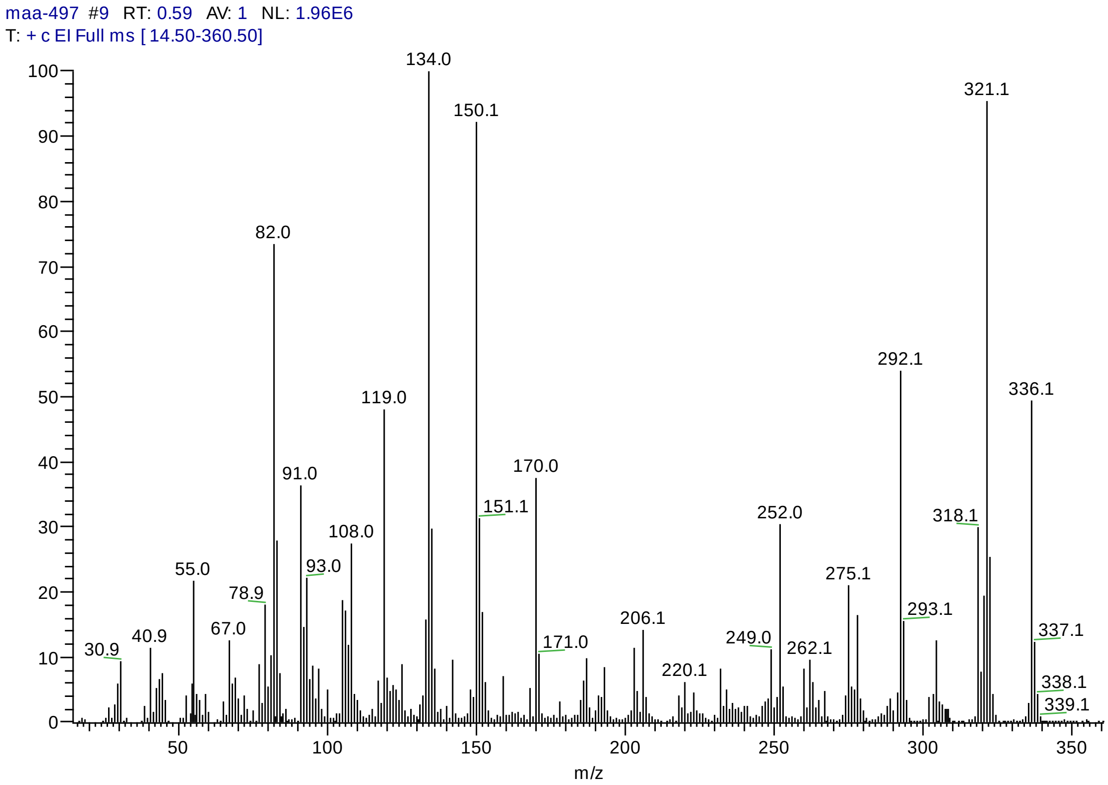

^{1}H NMR spectrum of compound **34a**

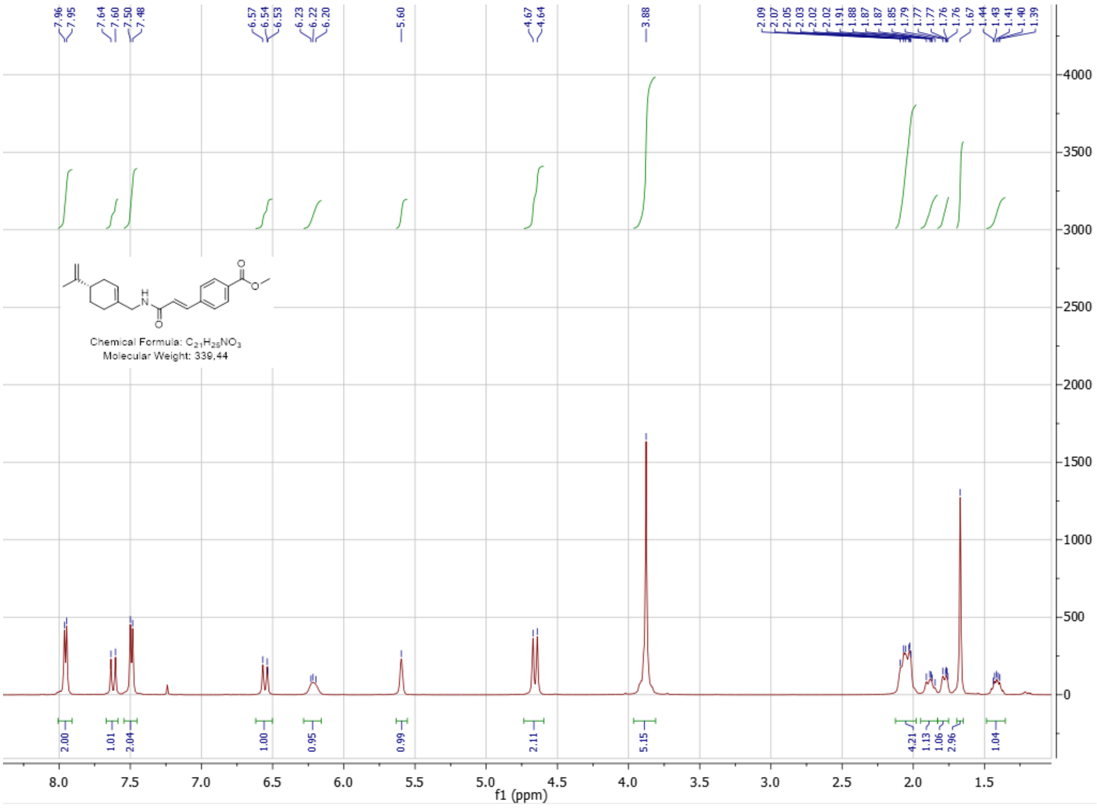

^{13}C NMR spectrum of compound **34a**

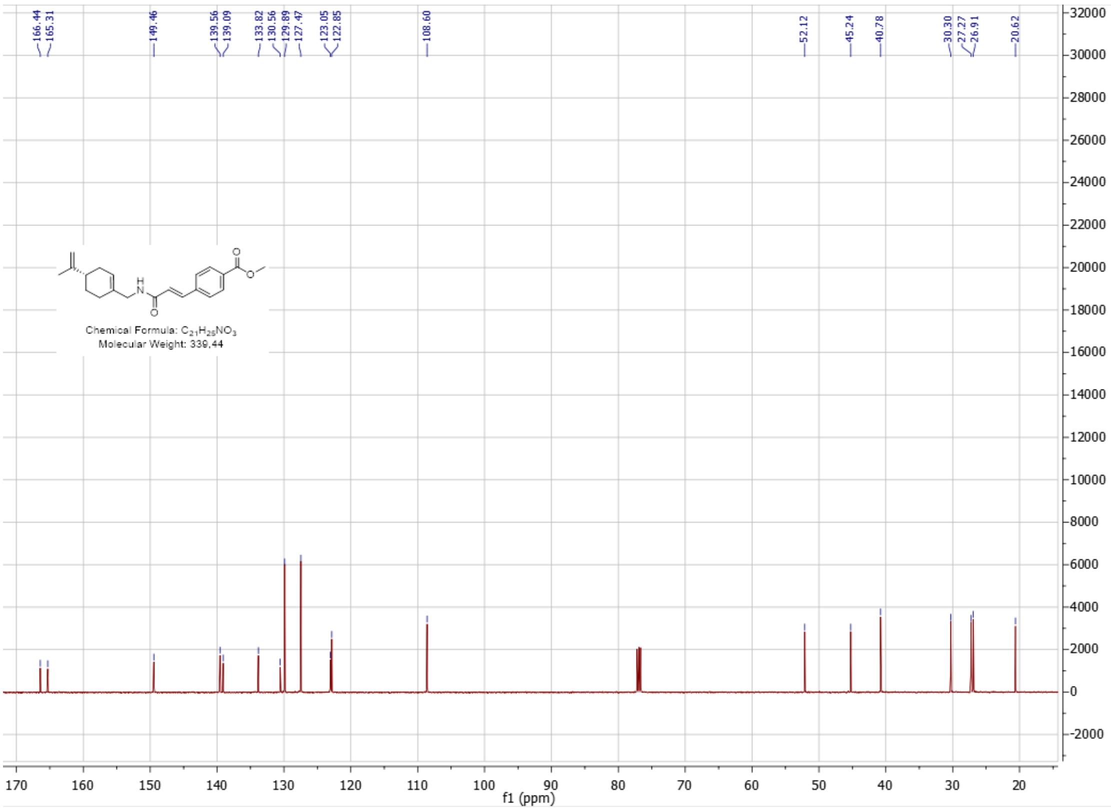

Mass-spectrum of compound **34a**

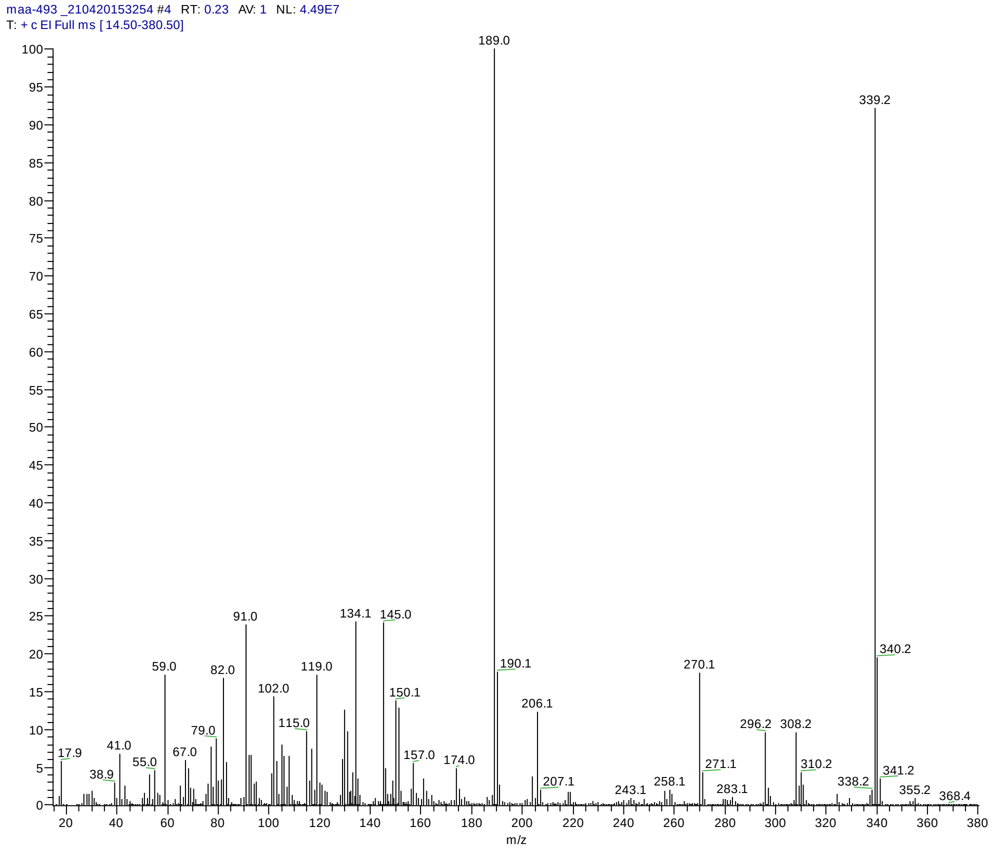

^{1}H NMR spectrum of compound **34b**

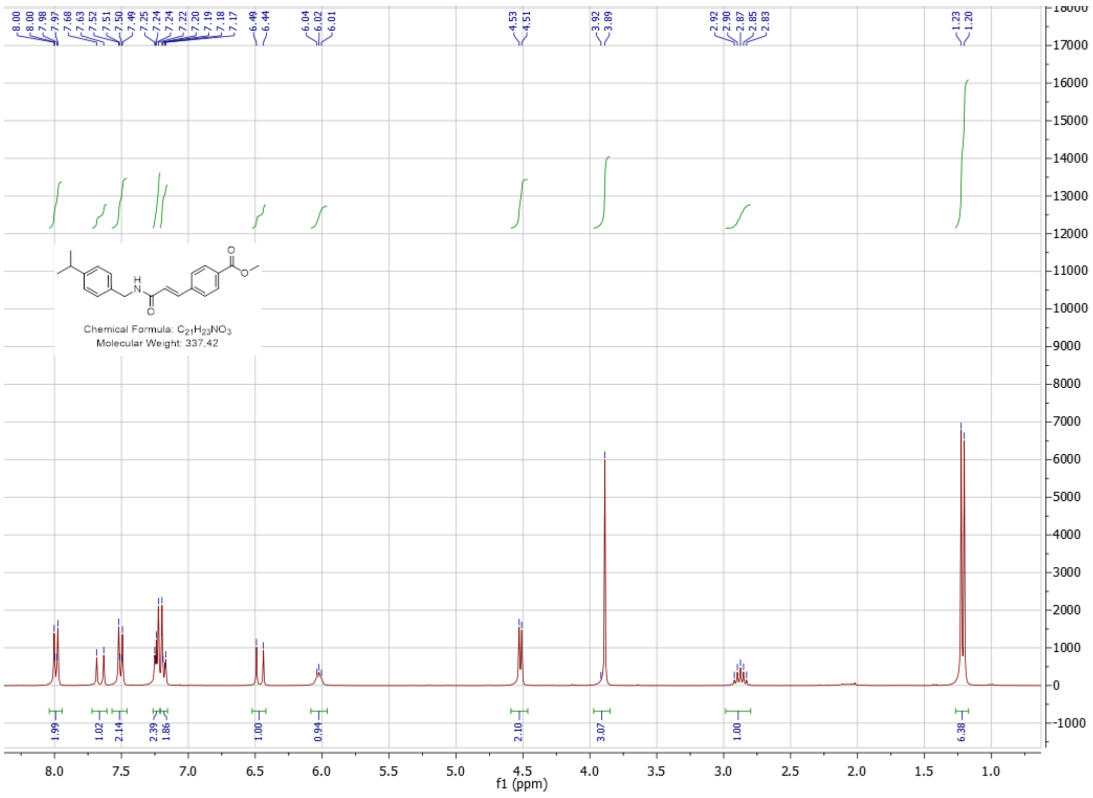

^{13}C NMR spectrum of compound **34b**

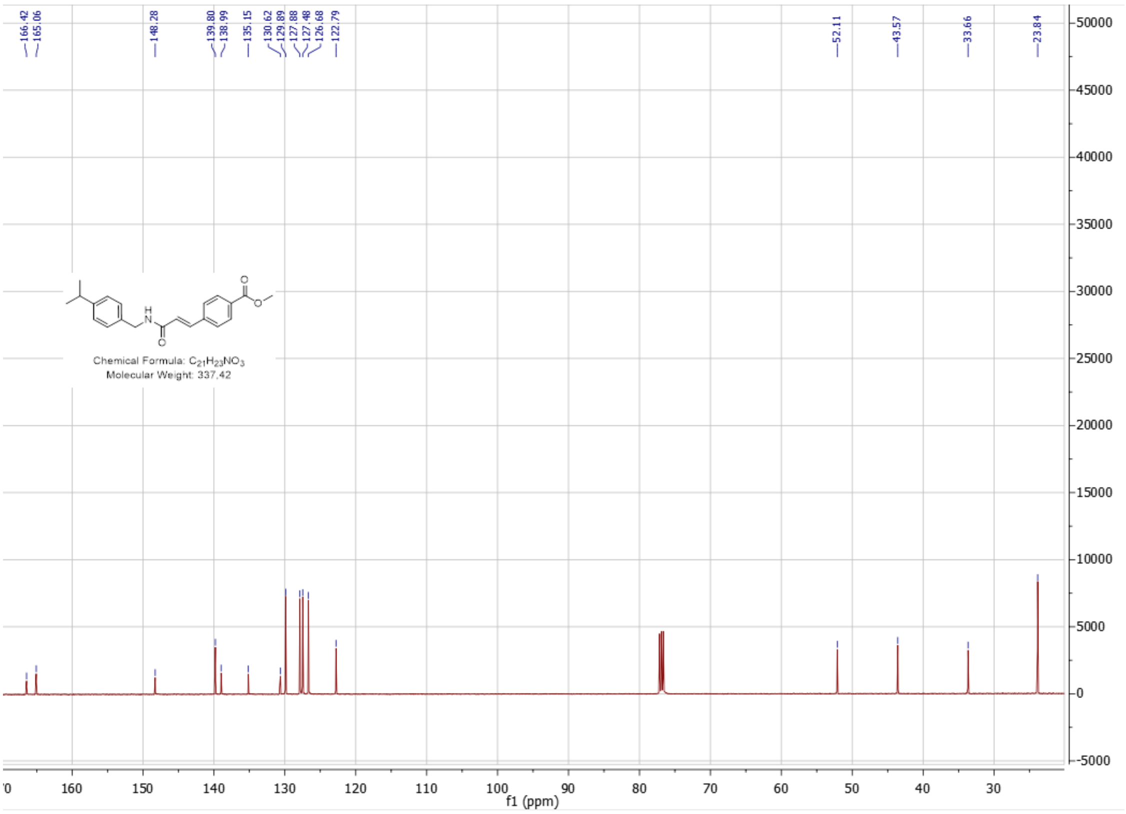

Mass-spectrum of compound **34b**

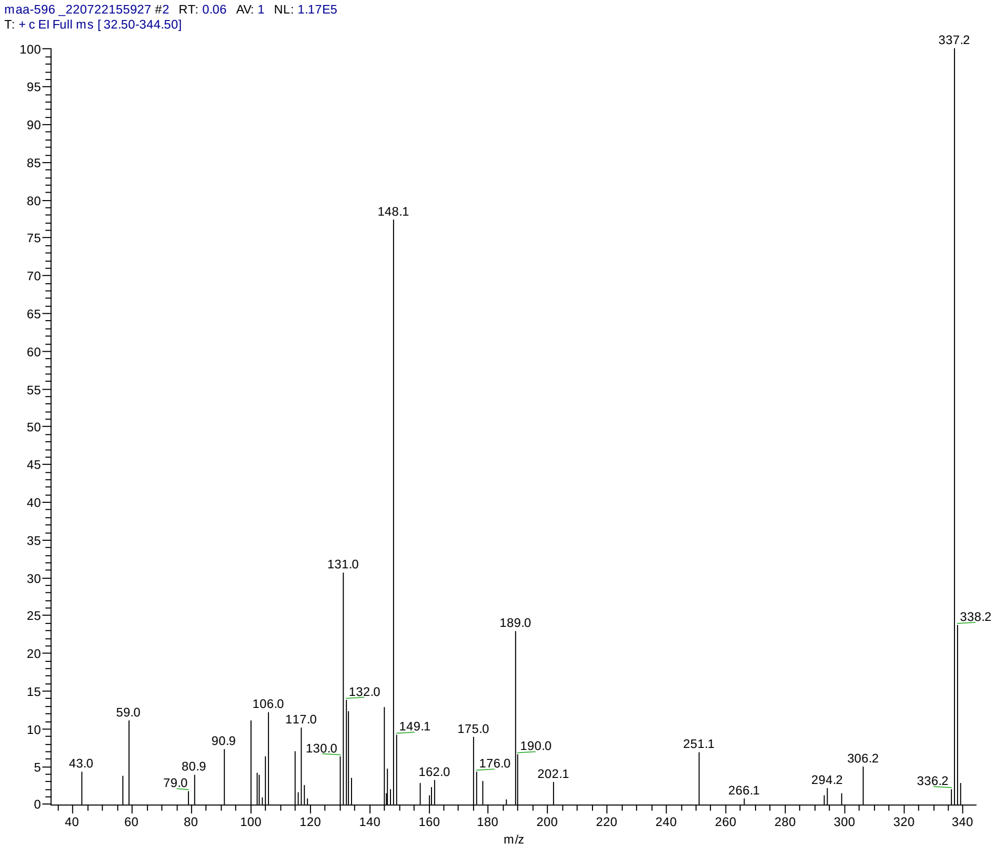

^{1}H NMR spectrum of compound **34c**

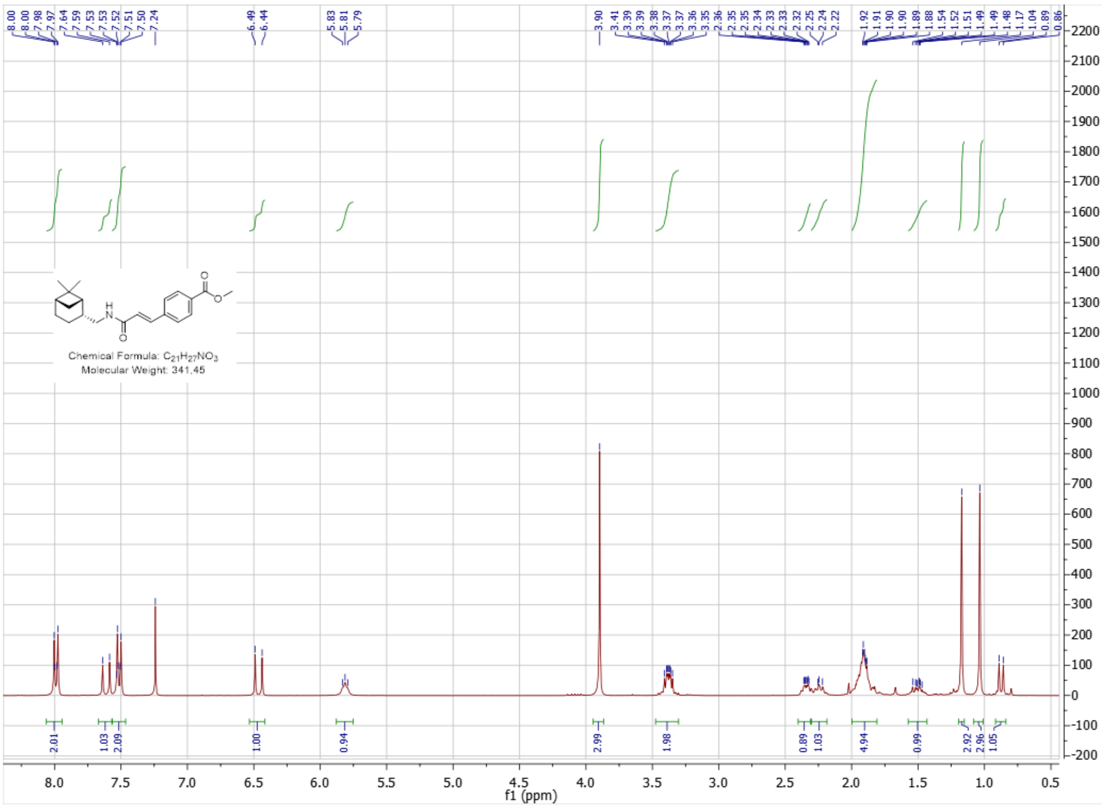

^{13}C NMR spectrum of compound **34c**

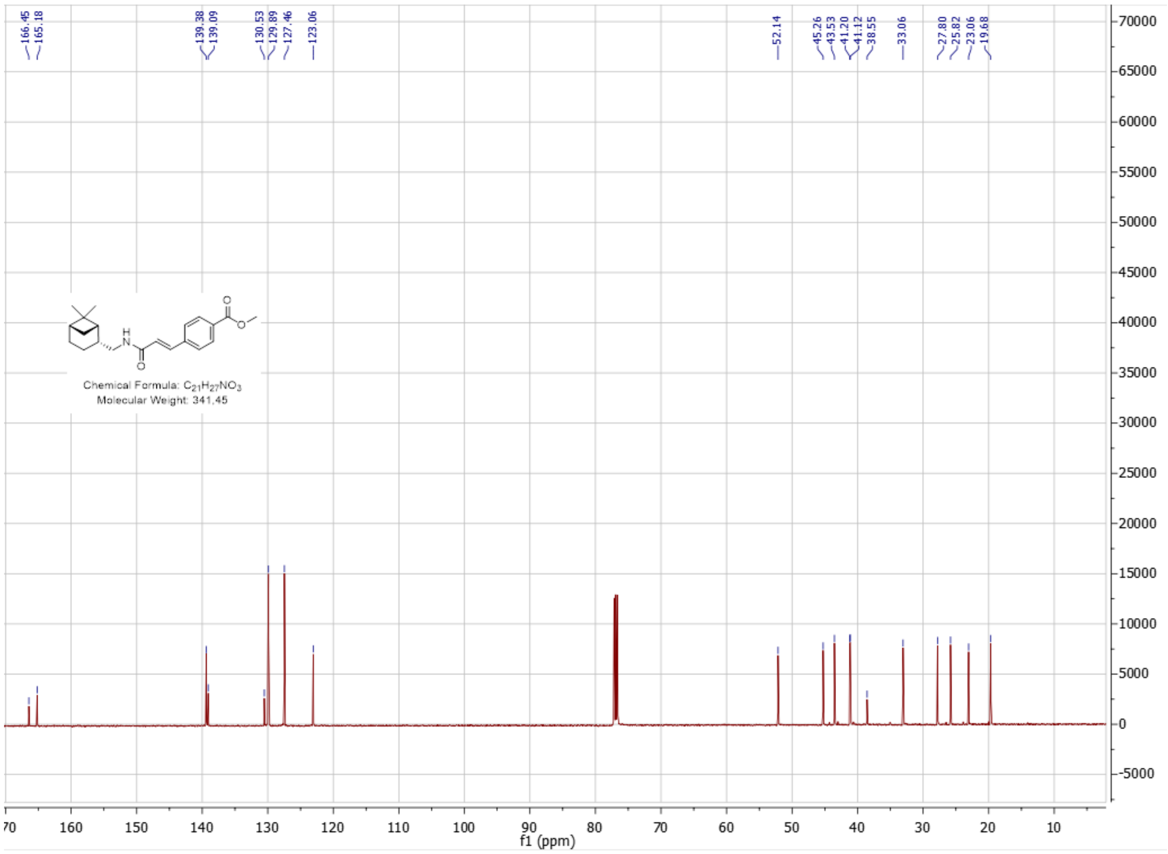

Mass-spectrum of compound **34c**

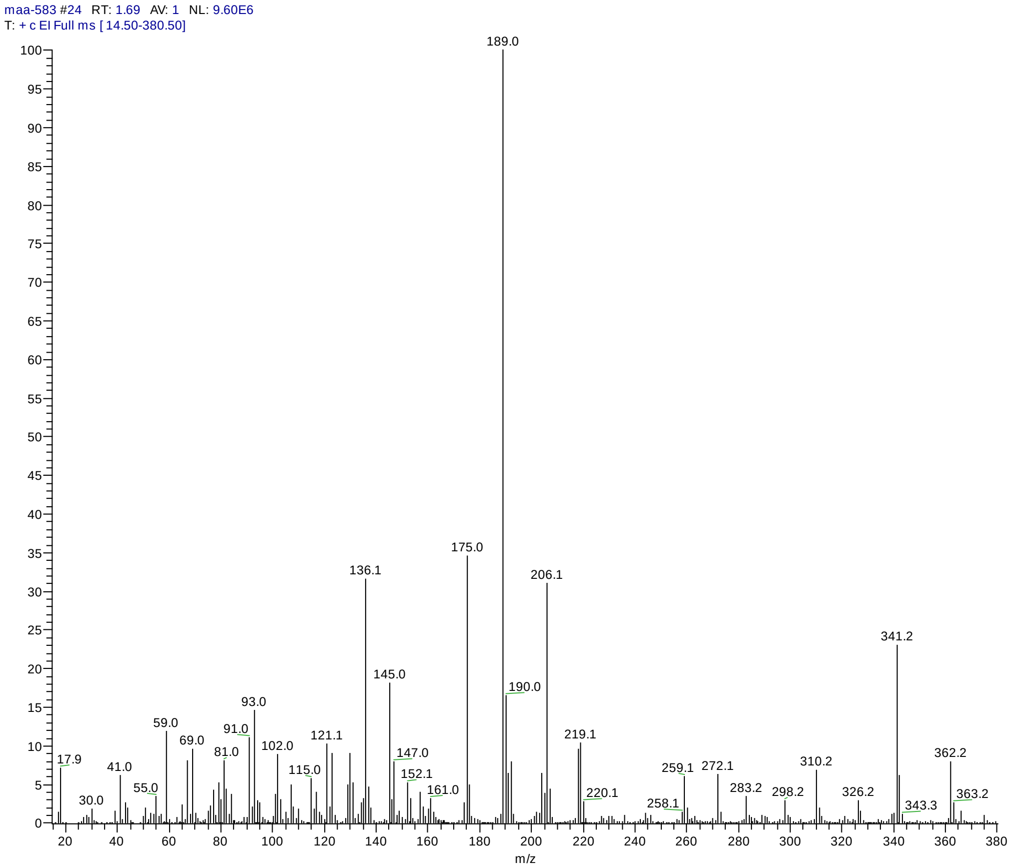

^{1}H NMR spectrum of compound **35a**

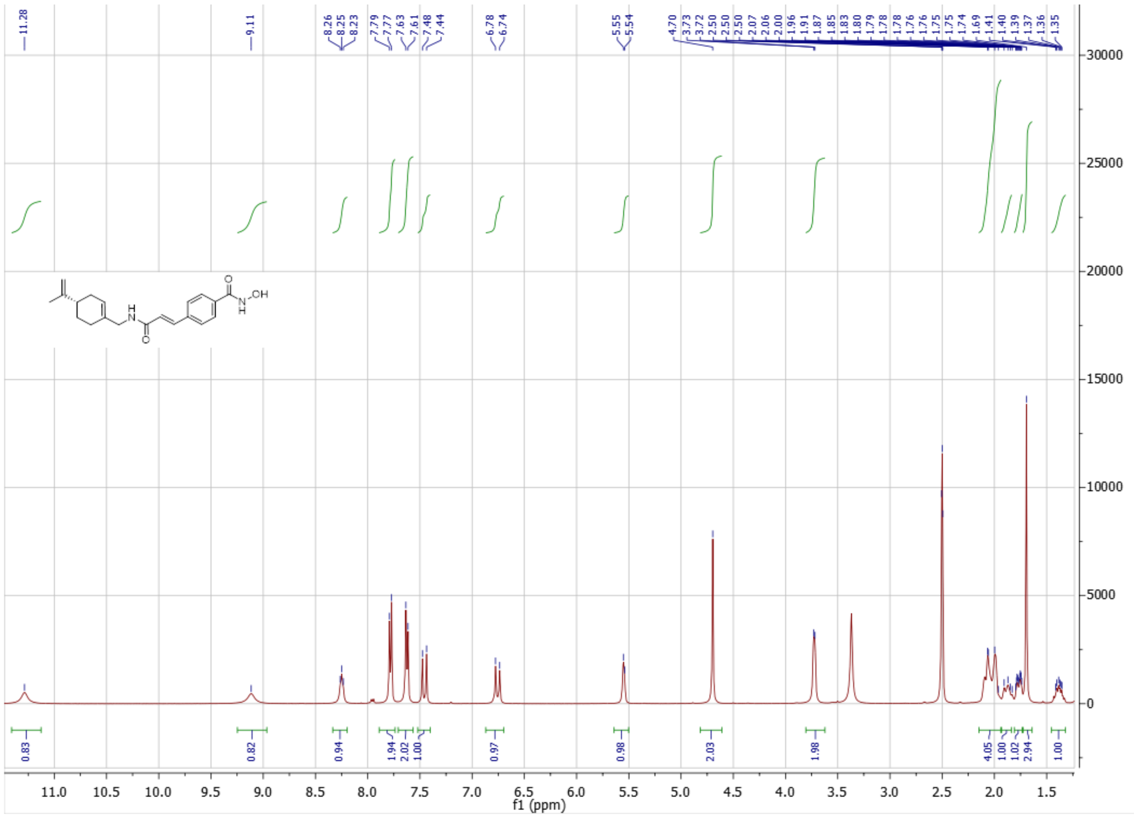

^{13}C NMR spectrum of compound **35a**

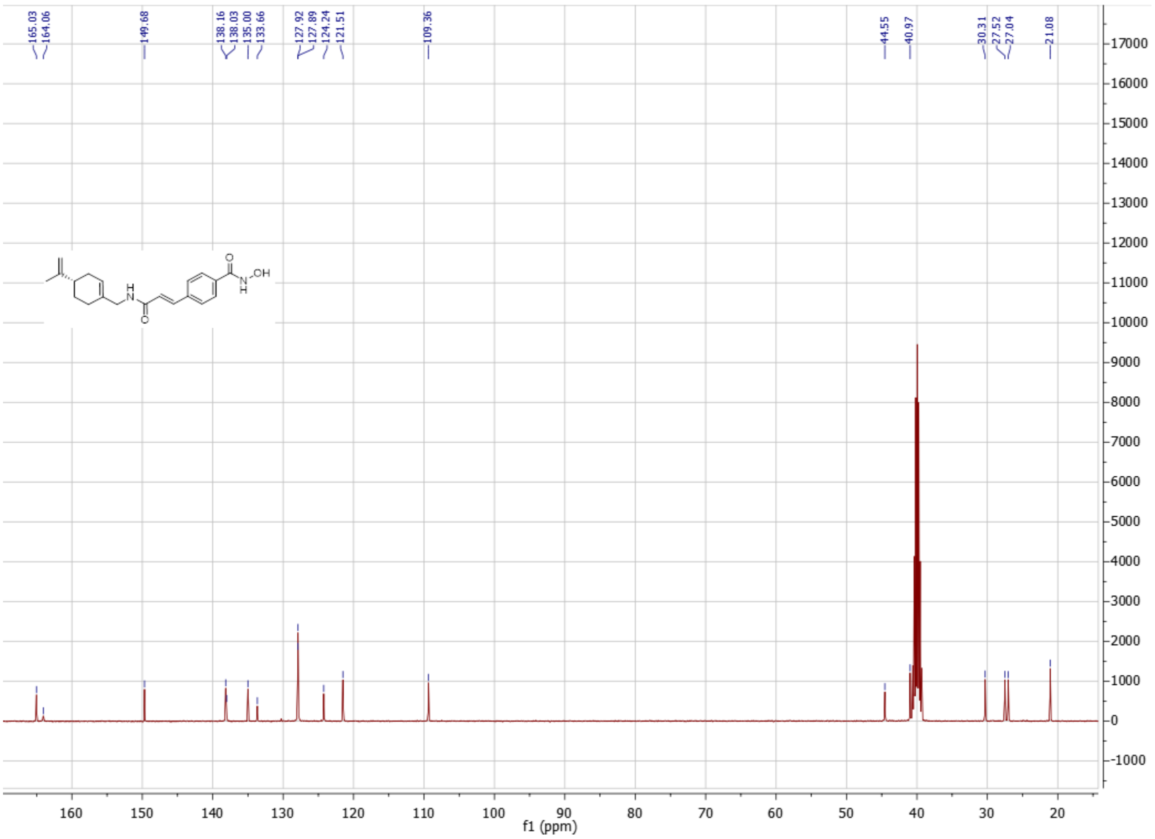

Mass-spectrum of compound **35a**

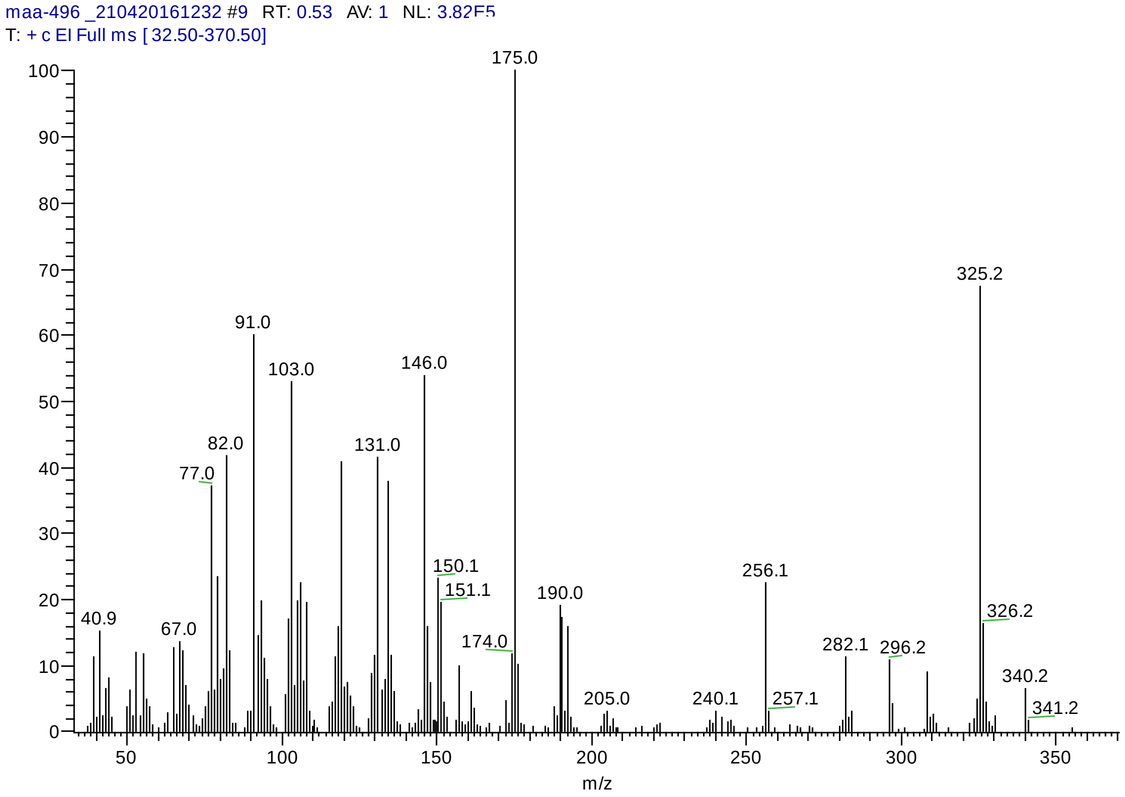

^{1}H NMR spectrum of compound **35b**

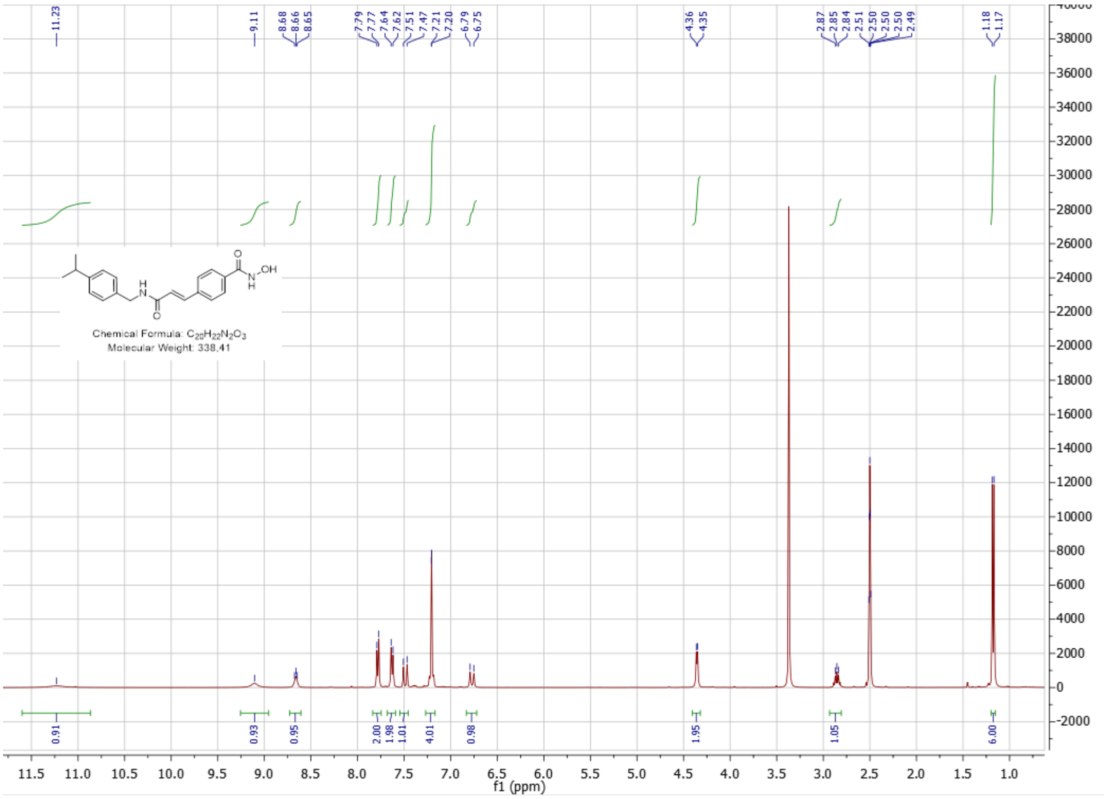

^{13}C NMR spectrum of compound **35b**

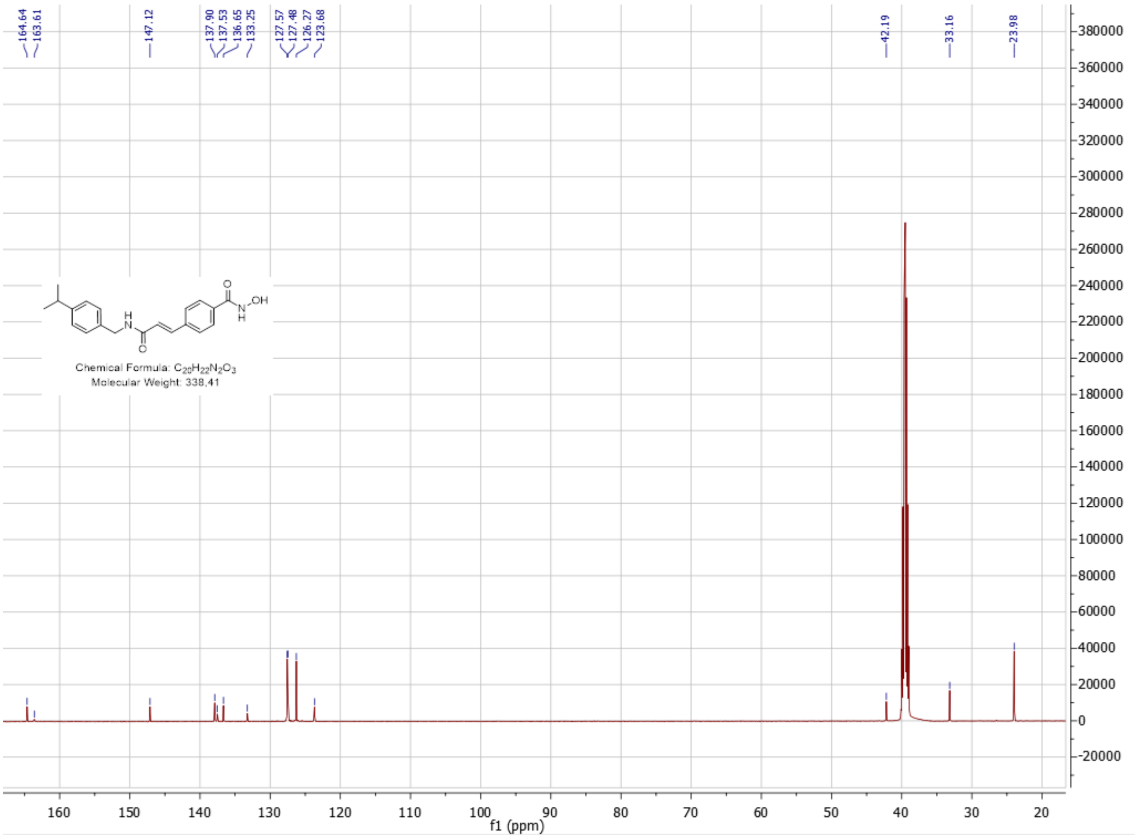

^{1}H NMR spectrum of compound **35c**

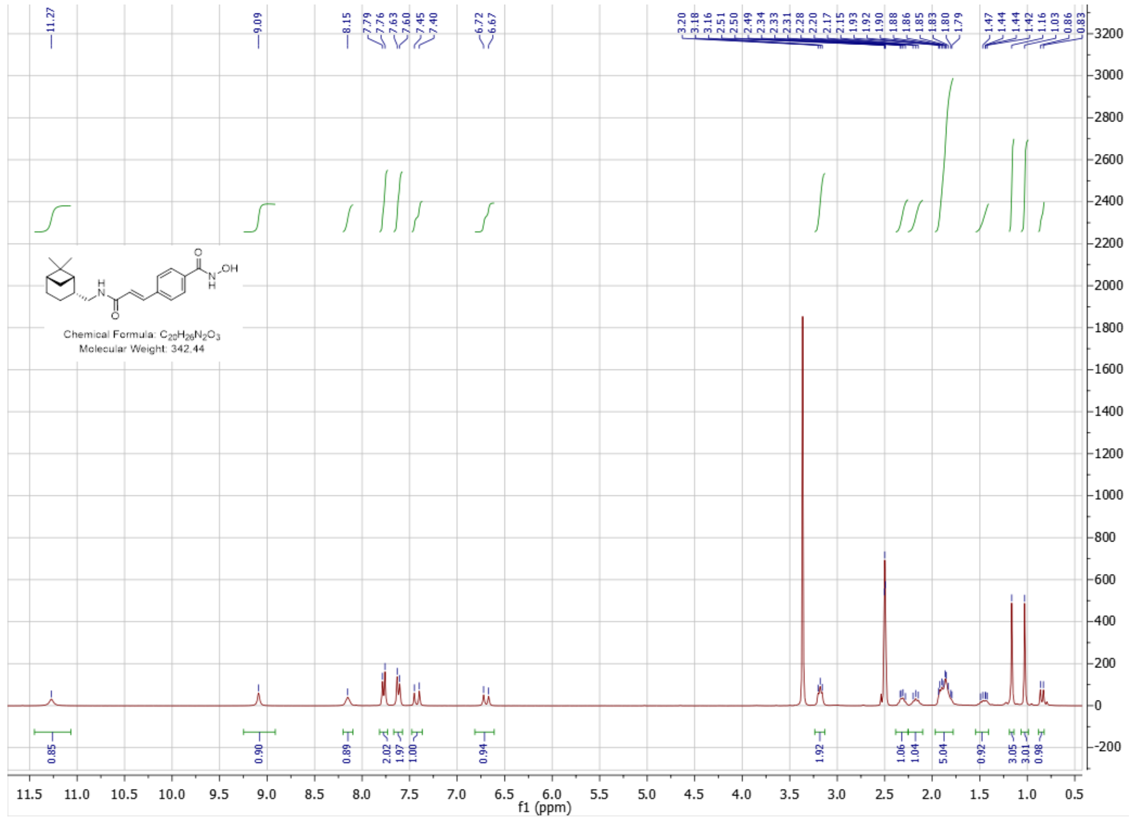

^{13}C NMR spectrum of compound **35c**

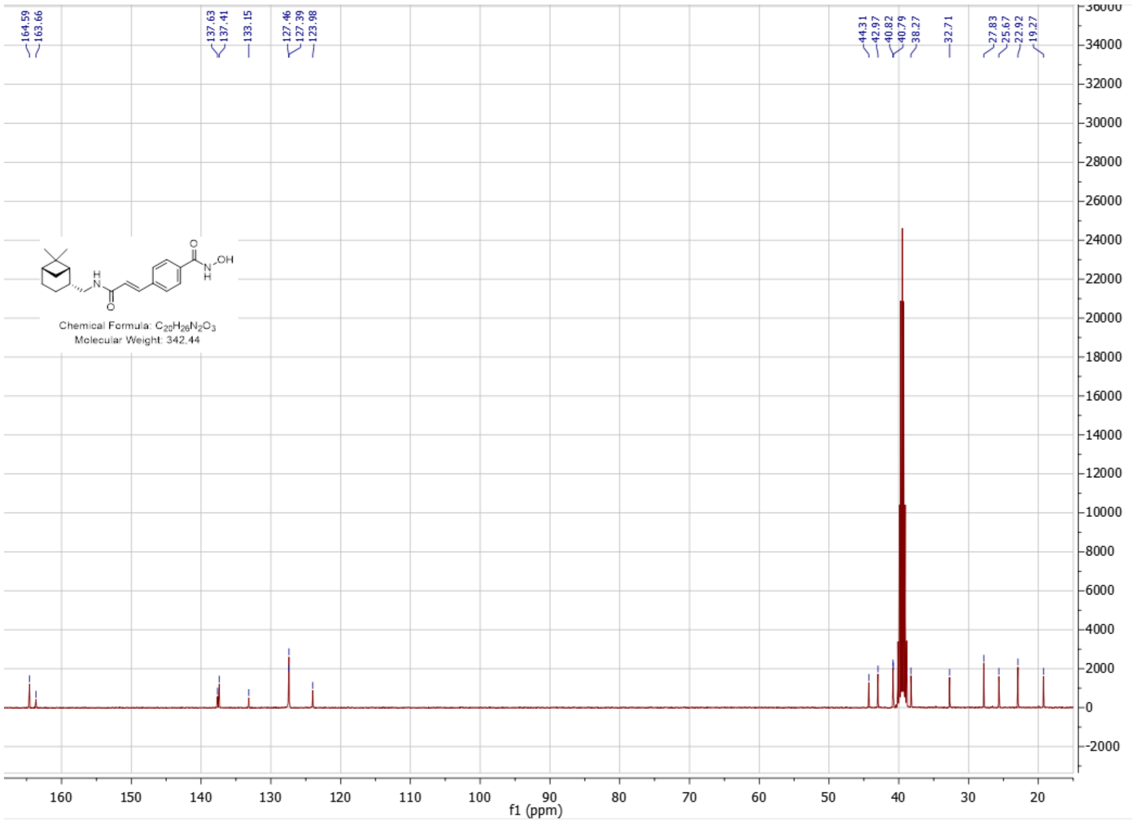

^{1}H NMR spectrum of compound **37a**

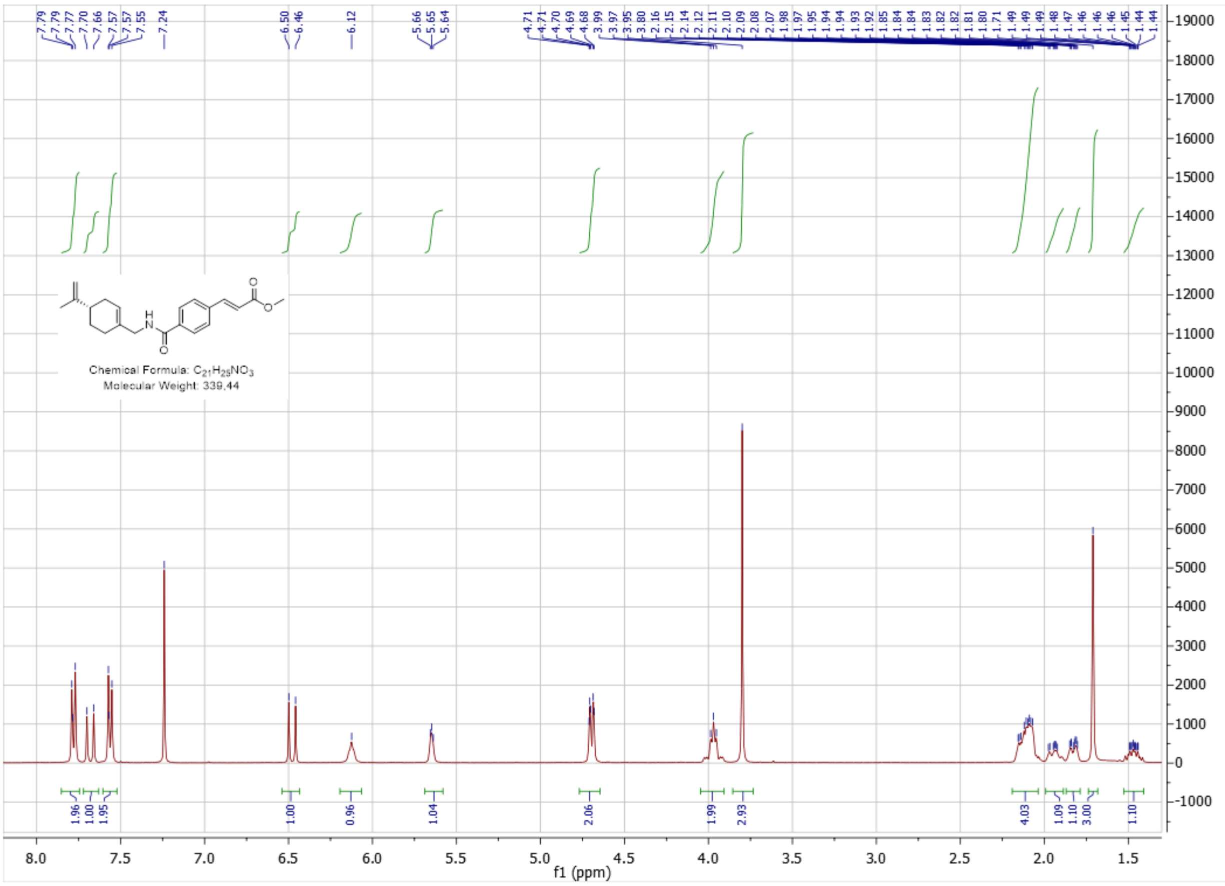

^{13}C NMR spectrum of compound **37a**

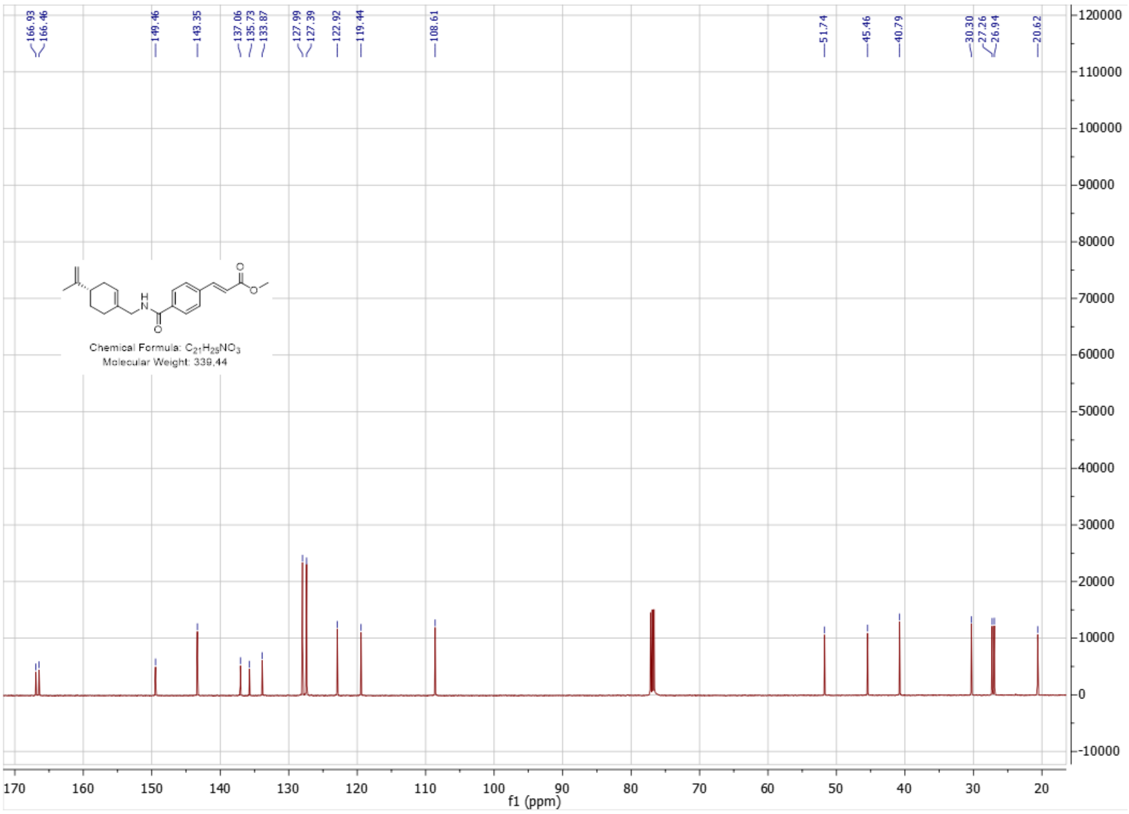

Mass-spectrum of compound **37a**

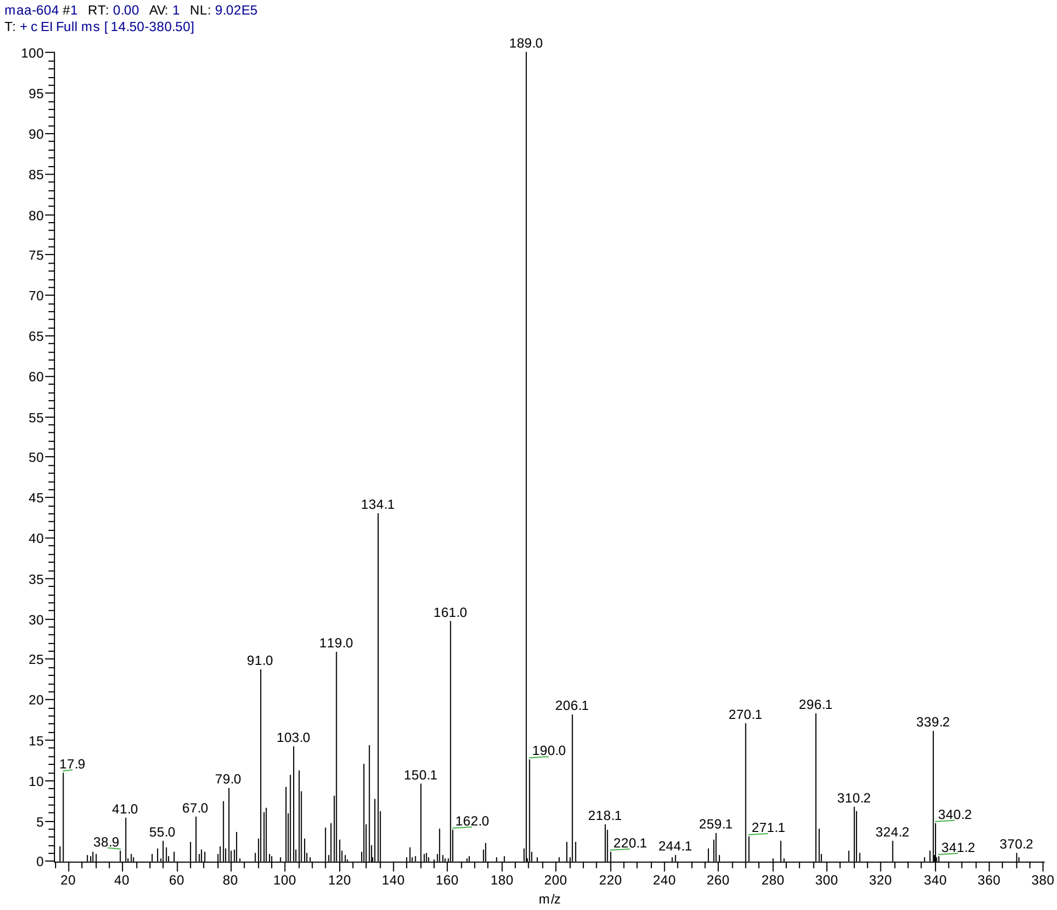

^{1}H NMR spectrum of compound **37b**

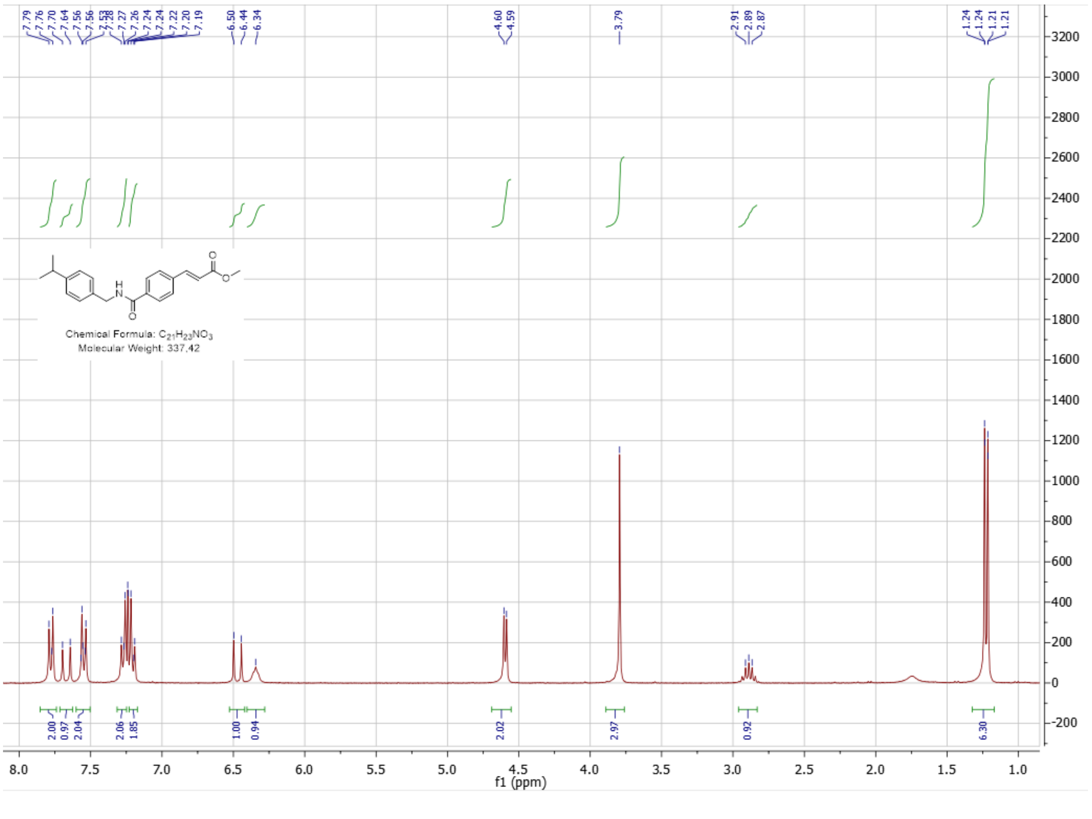

^{13}C NMR spectrum of compound **37b**

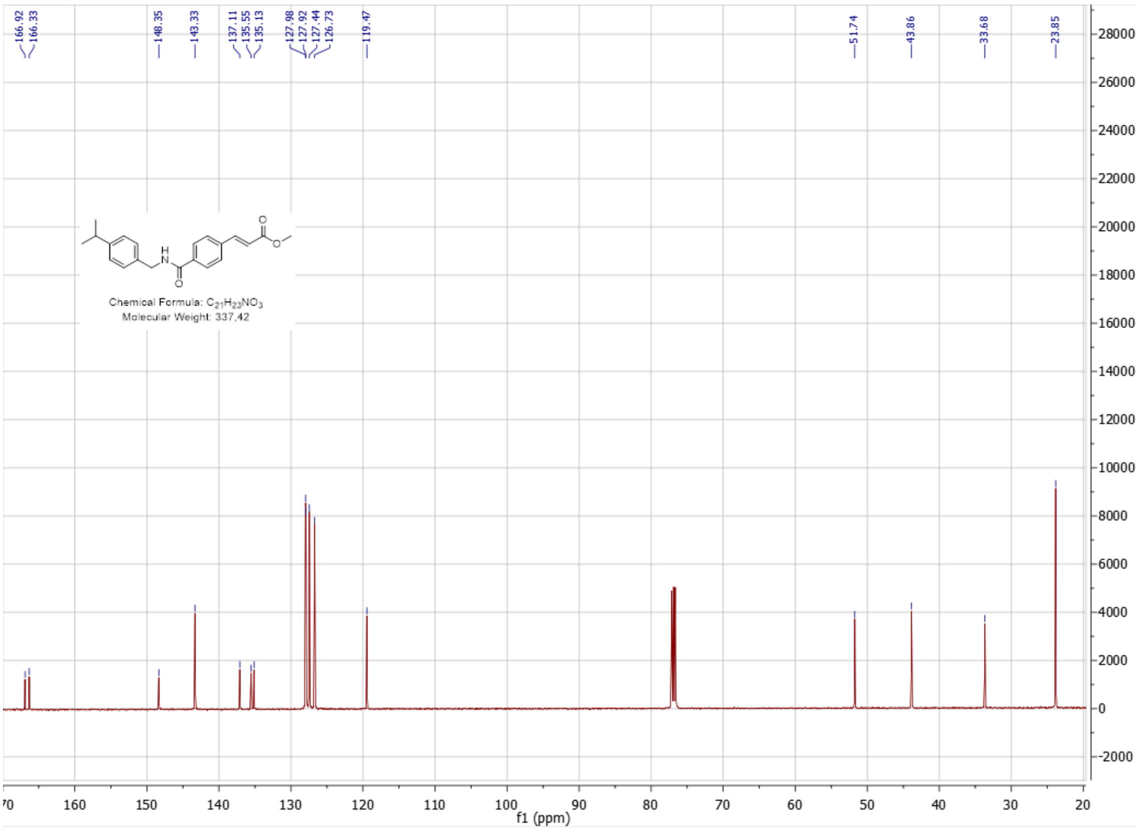

Mass-spectrum of compound **37b**

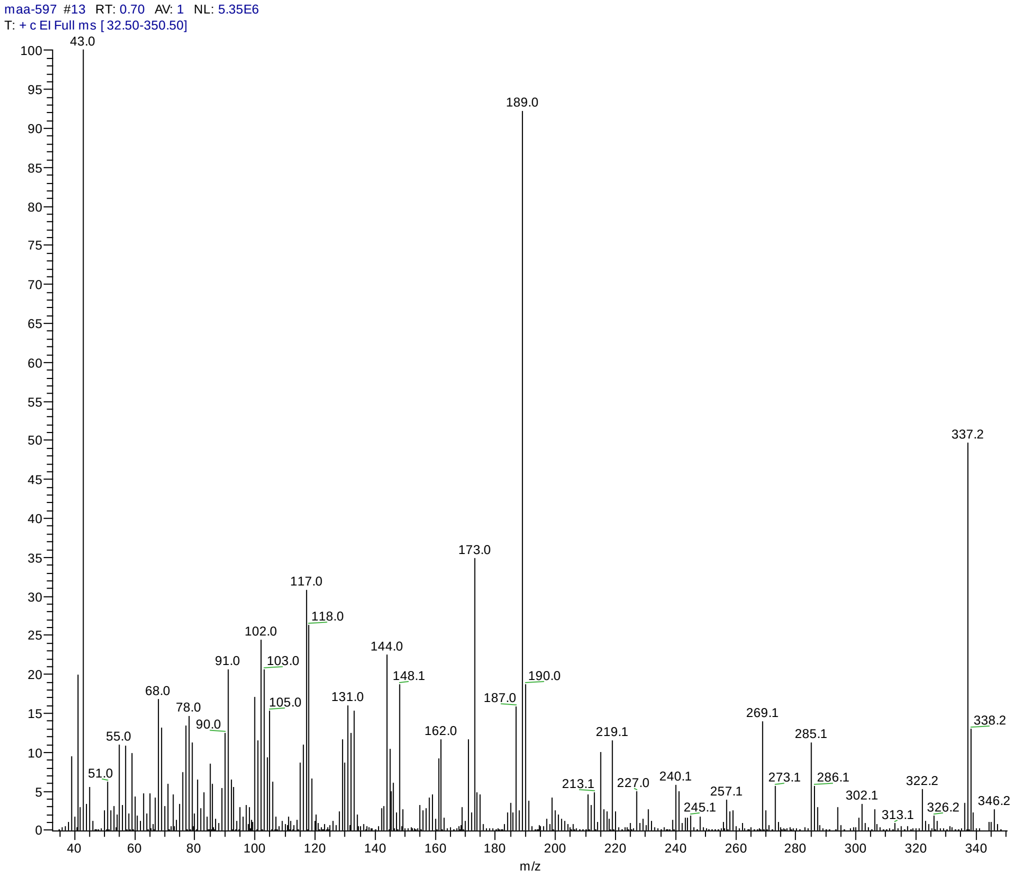

^{1}H NMR spectrum of compound **37c**

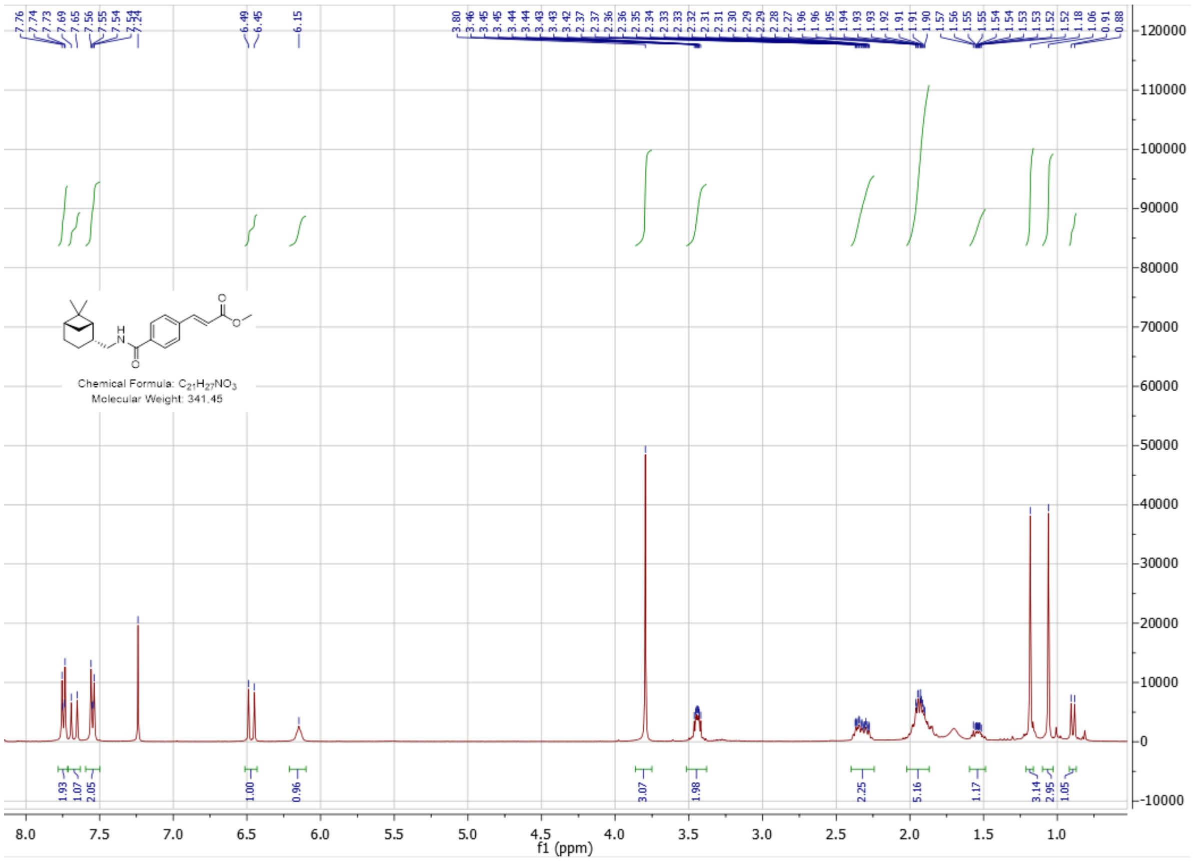

^{13}C NMR spectrum of compound **37c**

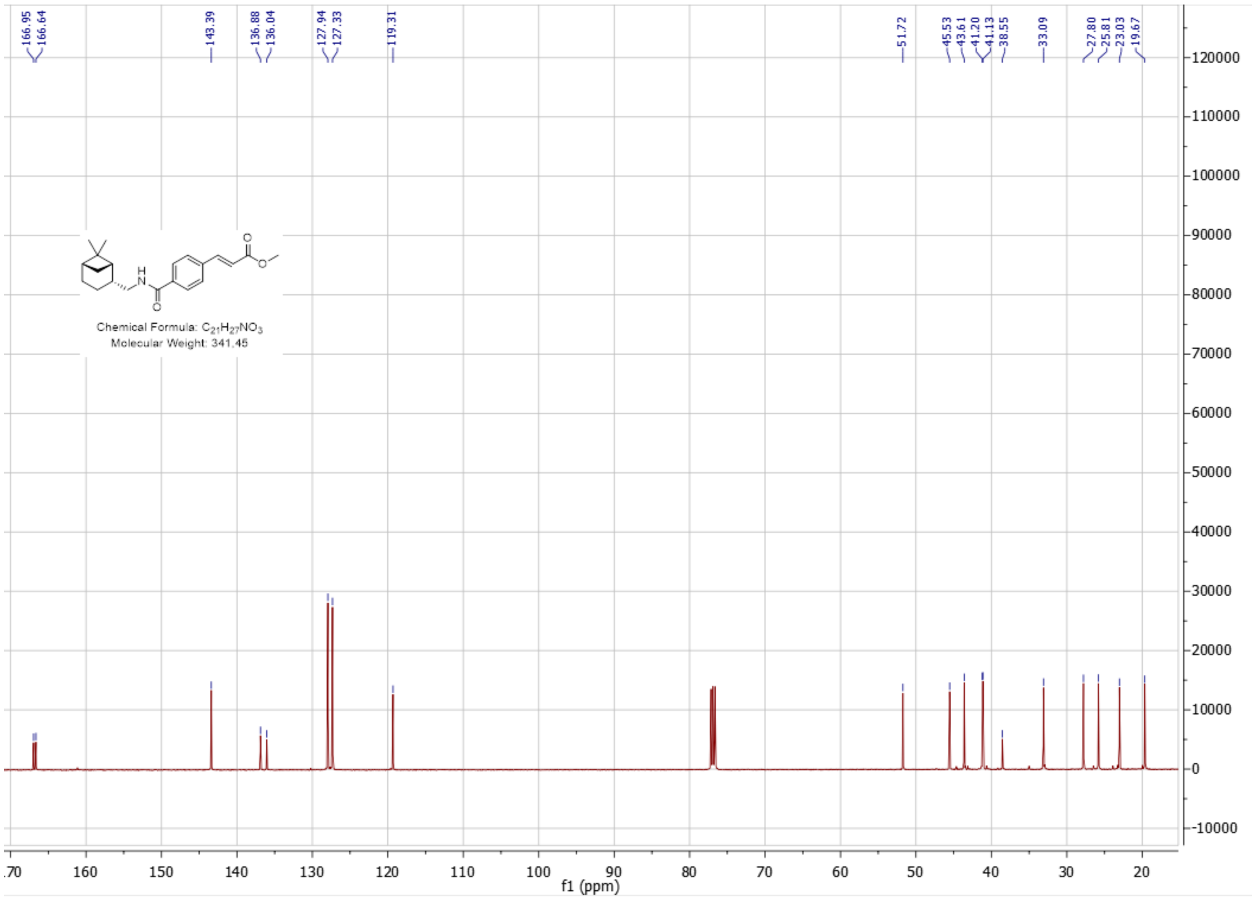

Mass-spectrum of compound **37c**

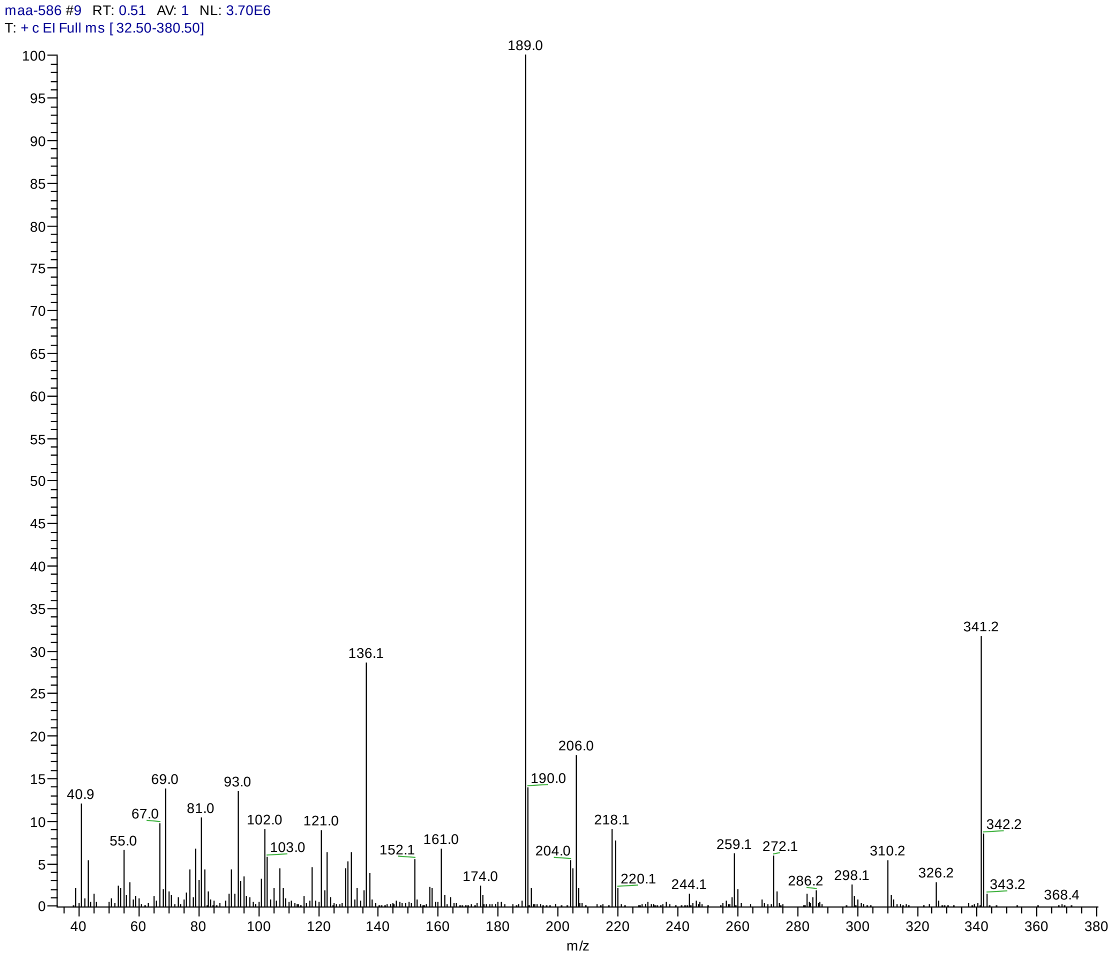

^{1}H NMR spectrum of compound **38a**

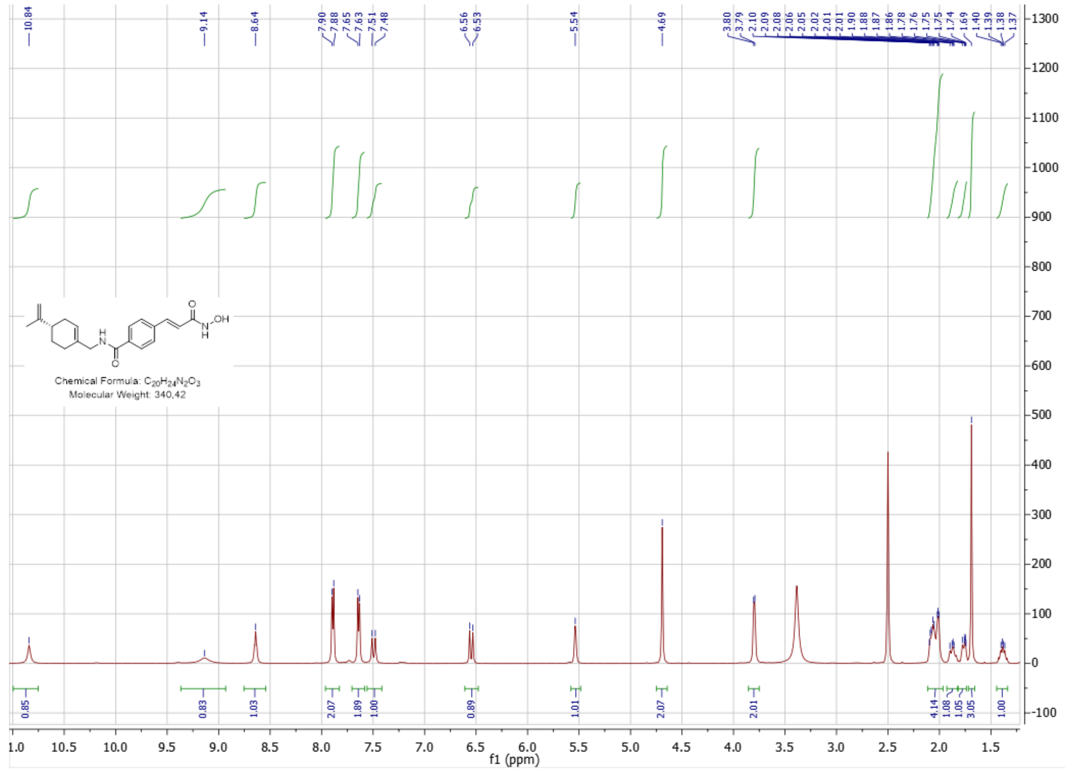

^{13}C NMR spectrum of compound **38a**

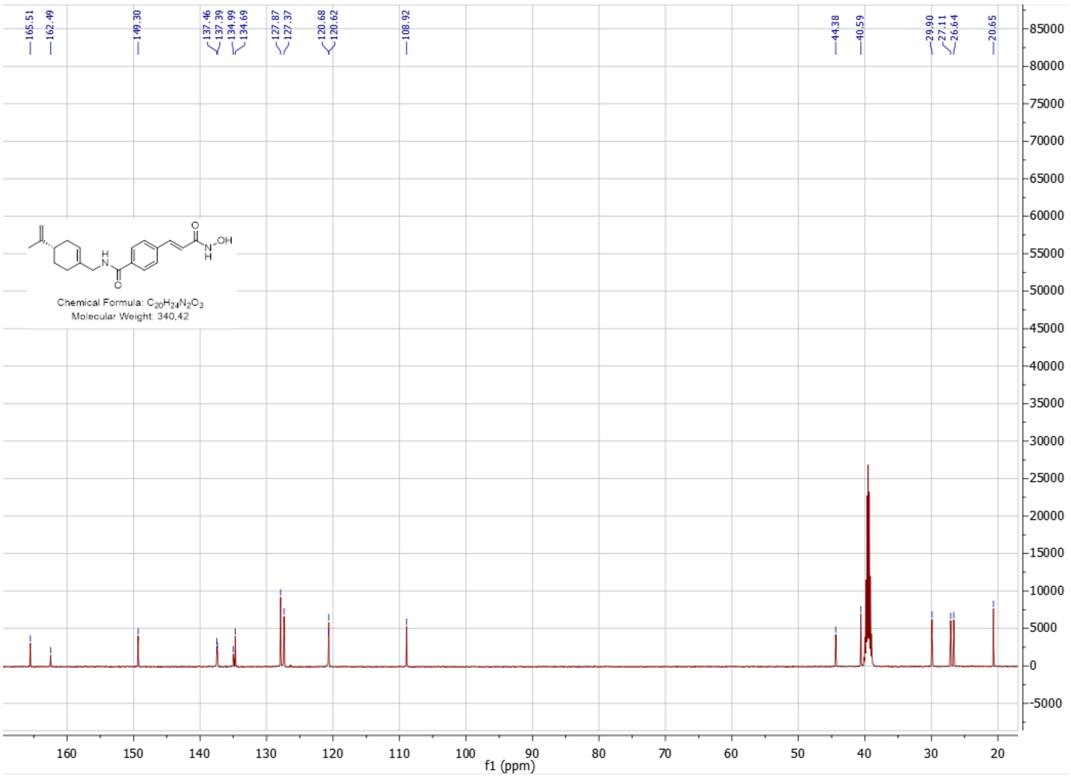

Mass-spectrum of compound **38a**

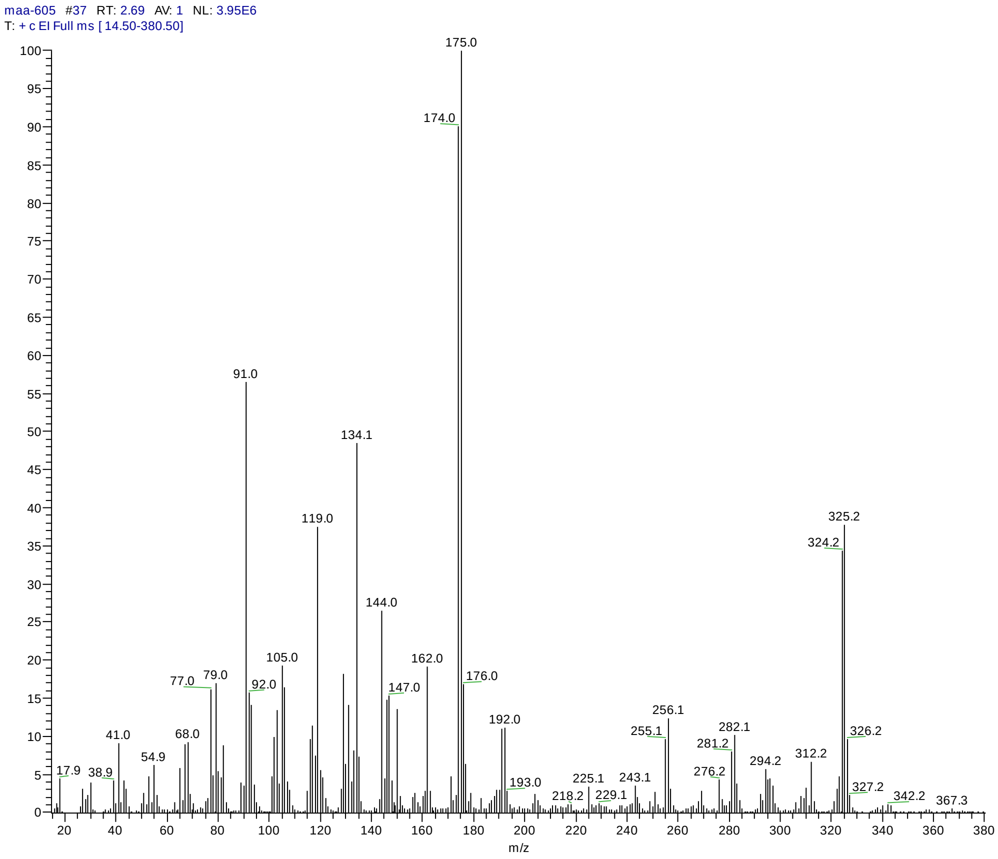

^{1}H NMR spectrum of compound **38b**

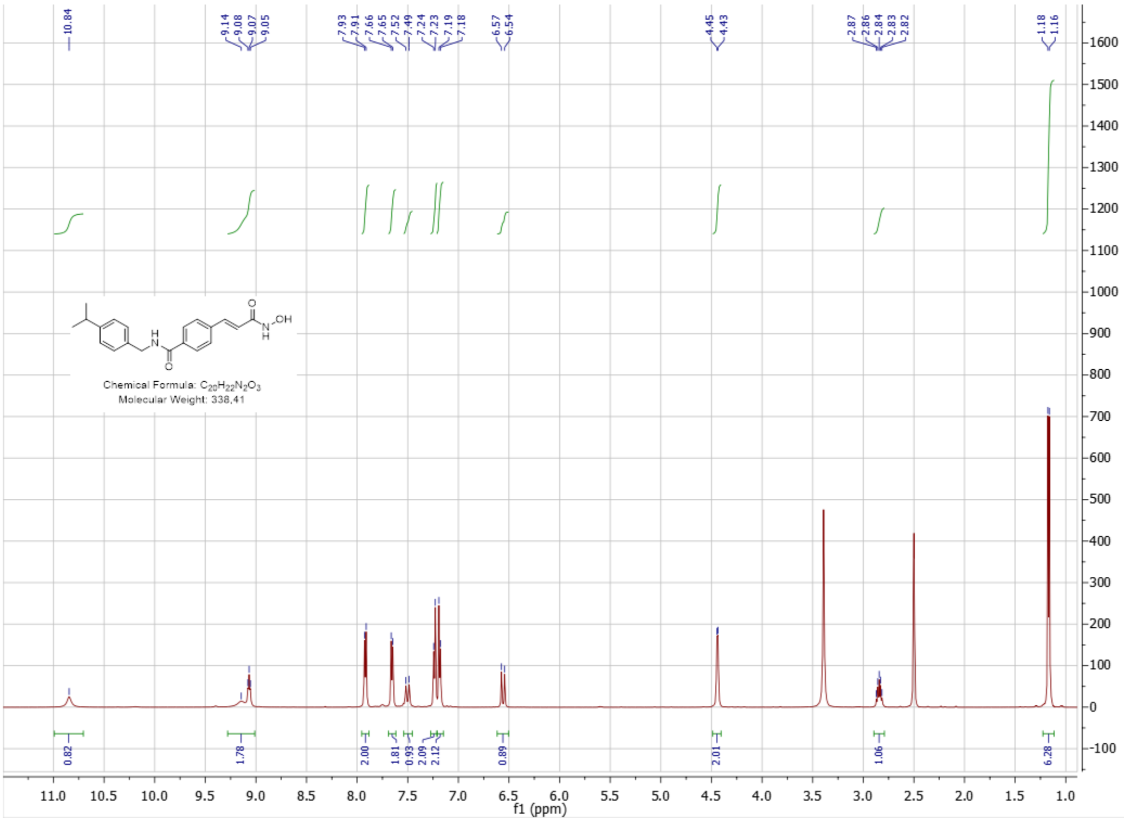

^{13}C NMR spectrum of compound **38b**

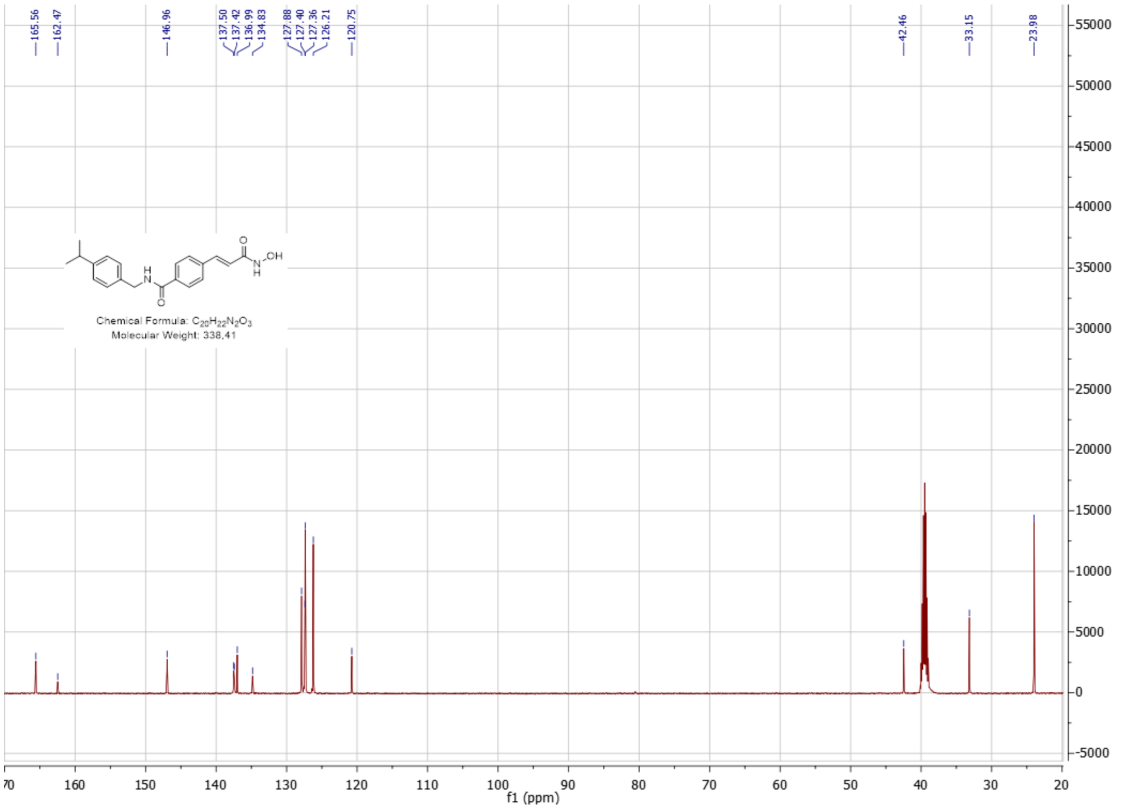

Mass-spectrum of compound **38b**

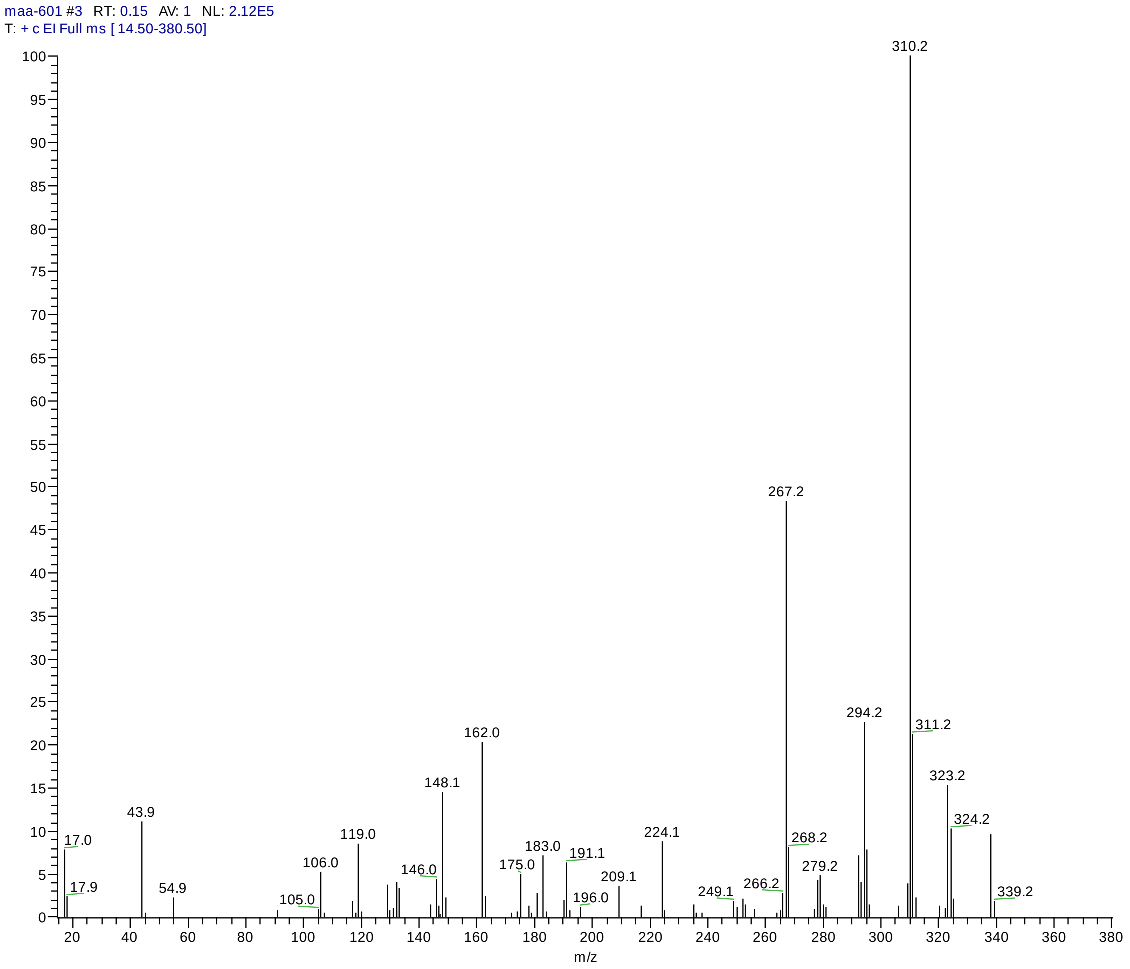

^{1}H NMR spectrum of compound **38c**

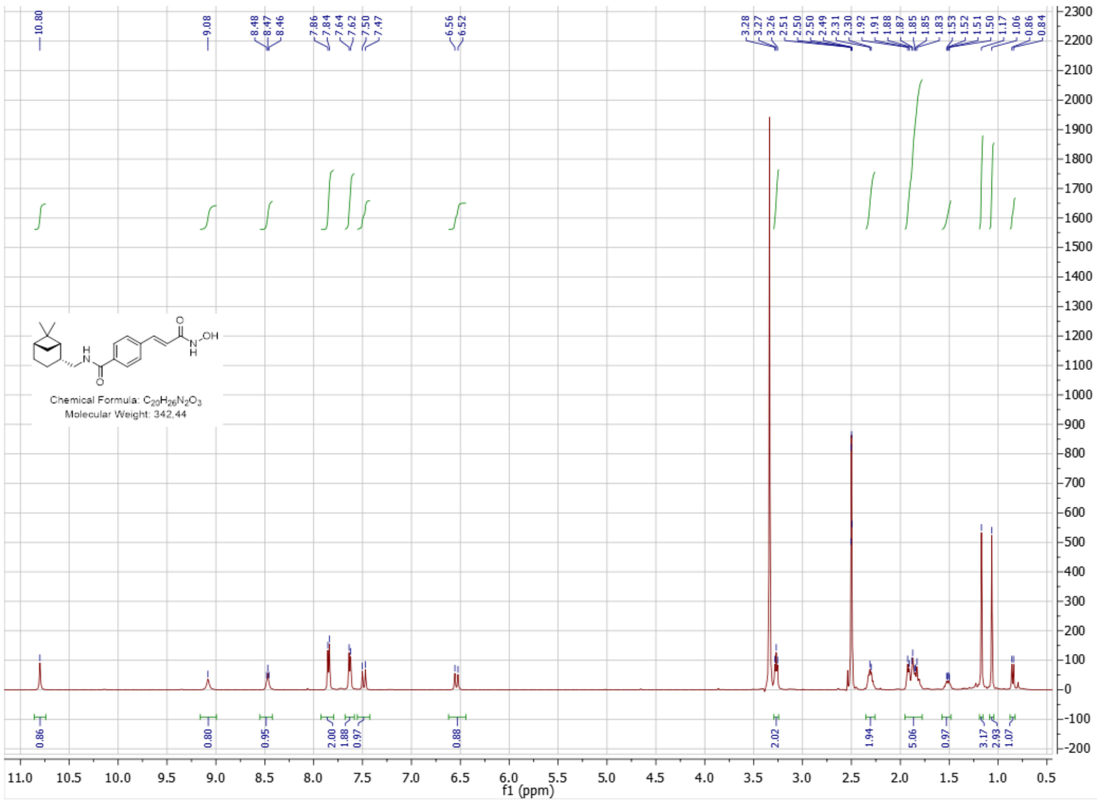

^{13}C NMR spectrum of compound **38c**

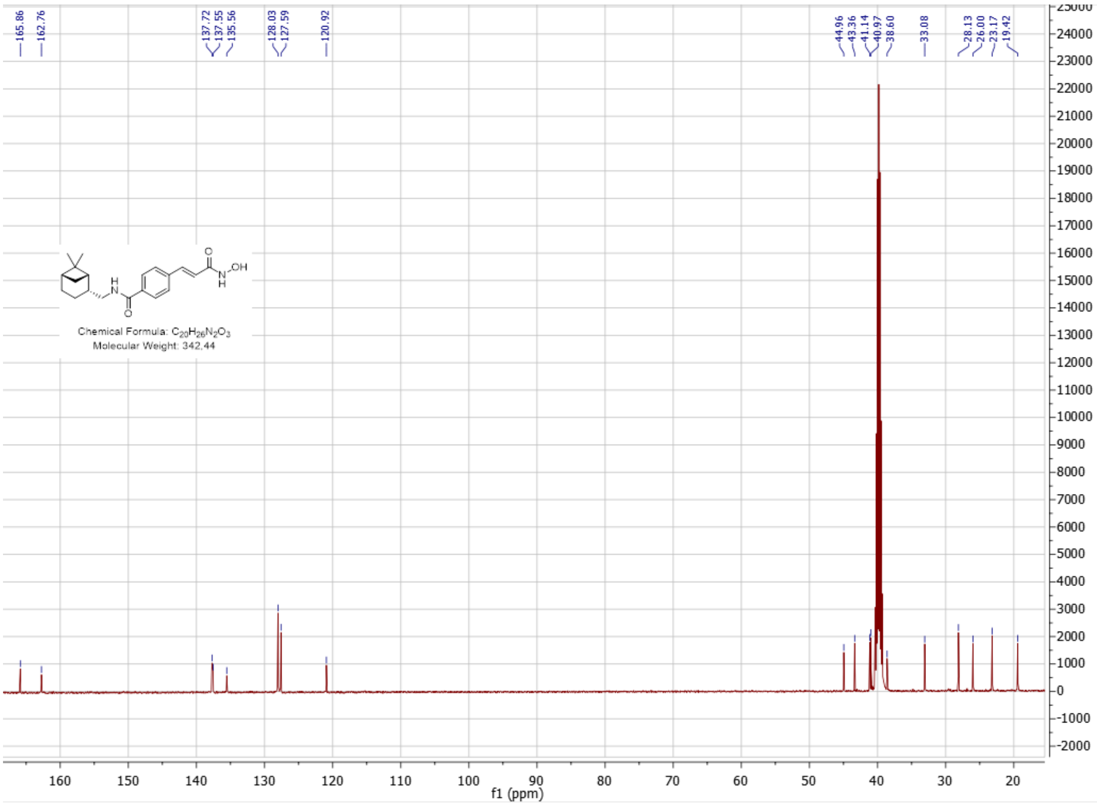

Mass-spectrum of compound **38c**

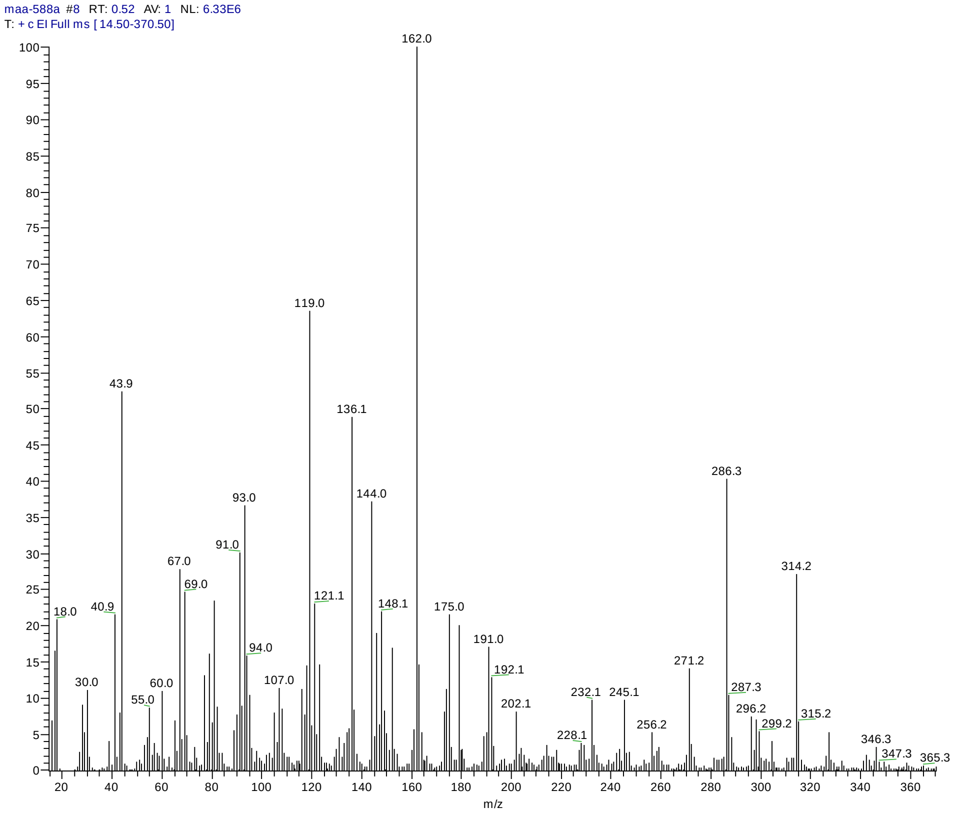

Table S1. Molecular docking of hydroxamic acids against HDAC6 (PDB ID: 5EDU) Hydrogen bonds are shown in the "H-bonds" column. Other interaction between peptide and ligand are shown in the "Other int-s".

Compound	H-bonds	Other interactions	Docking Score	IC_{50}, μM
**Trichostatin A**	HIS610	π-π stacking: HIE500	-8.798	
**31**	SER568 HIS610 TYR782	---	-9.547	0.66
**30**	SER568 GLY619 TYR782	---	-8.662	0.61
**35a**	HIE500 SER568 TYR782	π-π stacking: PHE620 HIE651	-8.120	3.87
**38b**	SER568	π-π stacking: PHE680	-7.125	4.81
**38a**	GLY619	π-π stacking: PHE680	-6.886	7.74
**35b**	HIE500 SER568 TYR782	π-π stacking: HIE500 PHE680	-6.689	8.23
**35c**	SER568 GLY619	π-π stacking: PHE620 HIE651 PHE680	-6.445	6.90
**38c**	TYR782	---	-6.210	8.03

Table S2. Results of docking procedures of molecules to the β-amyloid peptide (PDB ID: 1IYT). "C-term" corresponds to the C-terminus of the peptide (amino acids from 3 to 12, "N-term" corresponds to the fold in the vicinity of the N-terminus (amino acids from 22 to 30). Preferred position indicates the binding pocket with lower ΔG MM-GBSA. ΔG MM-GBSA is measured in kcal/mol. Hydrogen bonds are shown in the "H-bonds" columns. Other interaction between the β-amyloid and ligands are shown in the "Other int-s" columns.

Compound	Preferred position	ΔG $bind_{pref. pos}$ (MM-GBSA)	H-bonds (C-term)	Other int-s (C-term)	H-bonds (N-term)	Other int-s (N-term)	ΔG C-term (MM-GBSA)	ΔG N-term (MM-GBSA)	Statistical distribution C-term:N-term (T = 298 K)
**35b**	C-term	-45.66	HIE6 GLU11	π-π stacking: TYR10 PHE4	ALA21 GLU22 SER26 LYS28	--	-45.66	-42.01	480:1
**38a**	C-term	-44.7	GLU3 ASP7 GLU 11	π-π stacking: TYR10	ALA21 GLU22 SER26	--	-44.7	-42.13	77:1

**35c**	N-term	-43.57	GLU3<br>ASP7<br>GLU 11	π-π stacking: TYR10	GLU22<br>GLY25<br>SER26	--	-38.89	-43.57	1:2700
**38b**	N-term	-41.05	GLU 11	π-π stacking: TYR10	ALA21<br>GLU22<br>LYS28	--	-37.84	-41.05	1:230
**38c**	C-term	-40.19	ASP7<br>GLU11	π-π stacking: TYR10	GLU22<br>LYS28	--	-40.19	-32.74	290000:1
**35a**	C-term	-38.14	GLU11<br>GIE14	π-π stacking: TYR10	ALA21<br>GLU22<br>LYS28	--	-38.14	-36.54	15:1
